# Validity of a Cochrane Systematic Review and meta-analysis for determining the safety of vitamin E

**DOI:** 10.1186/s12906-017-1906-x

**Published:** 2017-08-16

**Authors:** Christopher J. Oliver, Stephen P. Myers

**Affiliations:** 1 Blackmores Institute, Warriewood, NSW 2102 Australia; 2 School of Health and Human Sciences, Southern Cross University, Lismore, NSW 2480 Australia; 3 NatMed-Research Unit, Division of Research, Southern Cross University, Lismore, NSW 2480 Australia

## Abstract

The public safety of α-tocopherol has been called in question by several meta-analyses which have raised concern among regulatory authorities. The objective of this study was to evaluate the Cochrane Database Systematic Review 2012 **(**CD007176) which concludes that α-tocopherol forms of vitamin E have a statistically significant effect on mortality, by assessing the trials and datasets used and determining their effect upon the primary outcome.

The Alpha-Tocopherol, Beta-Carotene Cancer Prevention Study (ATBC), a factorial design study of Finnish smokers was a pivotal paper in the Cochrane Review owing to the high mortality rate observed which resulted in a substantial weighting (42.6%) in the meta-analysis. The Cochrane meta-analysis used a 3 cell analytical method comparing all vitamin E cells (vitamin E alone plus vitamin E + β-carotene) to the placebo only cell. This had the unfortunate effect of incorrectly inflating the mortality risk attributed to vitamin E by not balancing the contribution to mortality of the β-carotene intervention. Re-analysis of the ATBC trial using data derived from the more generally accepted ‘inside the table’ (2 cell – vitamin E versus placebo) or ‘at the margins’ (4 cell – all vitamin E versus all non-vitamin E) analytical methods demonstrates a statistically non-significant result.

The data from the ATBC study has been given in 5 datasets (the trial alone and four extended post-trial follow-up time periods). Using the 3 cell analysis method only the 6 and 8-year (used in the meta-analysis) follow-up periods were statistically significant. Using the 2 or 4 cell method the outcome remains non-significant over all time periods.

The impartiality of excluding trials with zero mortality is also examined and questioned.

This paper raises concerns overall as to the appropriateness of datasets chosen, the validity of methods and generalisability of results when using meta-analysis as a tool to determine safety. Issues raised in this paper are not unique to the Cochrane study in question. Until we have new tools, there may be a need to rely on conventional narrative systematic literature synthesis in the assessment of safety or contain our results to specific sub-populations where more conclusive results can be determined.

**Electronic supplementary material:**

The online version of this article (doi:10.1186/s12906-017-1906-x) contains supplementary material, which is available to authorized users.

## Background

Over the last decade there have been several meta-analyses examining the effect of vitamin E on all-cause mortality [1–4] which have drawn the conclusion of a positive association between vitamin E supplement use and mortality. It is important to recognise that vitamin E in the context of these meta-analyses refers only to alpha-tocopherol which is only one of the eight isoforms of the vitamin (alpha, beta, gamma and delta tocopherols; and the alpha, beta, gamma, and delta tocotrienols). To remain consistent to the terminology used in the Cochrane review which is the focus of this paper we continue to use the term ‘vitamin E’ in the acknowledgement that our use pertains only to the synthetic and natural forms of alpha-tocopherol used in the various trials considered. While there has been some criticism of these meta-analyses [5, 6] they have not only received considerable media attention but also frequent citation in scientific papers; the papers by Bjelakovic et al. [1–3] have been cited over seventeen hundred times (Scopus 31st December 2016).

We set out to evaluate the validity of the 2012 systematic review on vitamin E published by the Cochrane Collaboration [3] by examining the included trials and the data used and the conclusions drawn. This study was chosen because the earlier 2008 meta-analysis on vitamin E published by the Cochrane Collaboration [2] was the main publication cited by the Australian drug regulator (Therapeutic Goods Administration) whom had requested industry consultation on its intention to change the regulatory status of vitamin E (2008). In addition, this meta-analysis provides the most transparent methods and comprehensive datasets due to the Cochrane Collaboration’s guidelines for publication.

## Methods

### Data source

The 2012 Cochrane meta-analysis [3] was retrieved from the Cochrane website and data from the table ‘*Analysis 01.11. Comparison 01 Antioxidants versus placebo/no intervention, Outcome 11 Mortality in vitamin E trials with a low or high risk of bias’* (page 250) were used. For ease of description this data table will be abbreviated as A11. Trials were assigned to either low or high risk of bias by Bjelakovic et al. from an assessment of the methodology reported for each trial in the following six domains: allocation sequence generation, allocation concealment, blinding, complete outcome data reporting, selective outcome reporting, and other apparent biases. Data analysis in the results section of our paper pertains to the low risk of bias subgroup only and not the total analysis (which includes both high and low bias sub-groups) which replicates the primary findings in the Bjelakovic et al. paper.

### ATBC study

Examination of A11 showed that one paper, the ATBC 2003Low (Alpha-Tocopherol, Beta-Carotene Cancer Prevention Study, dataset from 2003 publication, low bias study) was given a weighting of 42.6% in the analysis. The next closest study (HPS 2002) has a weighting of 12.7%. Given the important contribution of the ATBC study to the meta-analysis outcome pertinent publications concerning the ATBC trial were retrieved [7–9].

### Meta-analyses

Meta-analysis calculations were carried out using the Review Manager (RevMan) software (Version 5.2. Copenhagen: The Nordic Cochrane Centre, The Cochrane Collaboration, 2012). In any analysis the risk ratio and 95% confidence intervals were calculated using the Mantel-Haenszel method for dichotomous data in a random effects model to replicate the methods used in the Bjelakovic meta-analysis [3].

### Data analysis

To commence, the data from A11 was copied into RevMan and checked against the original table for accuracy.

To determine the contribution of the ATBC 2003 study a sensitivity analysis was undertaken by removing this study from the A11 low risk of bias subgroup and re-running the analysis.

To determine the sensitivity of the meta-analysis to trial data that explicitly would show a high level of safety, a large fictitious trial was added to A11 in the low risk of bias sub-group (with ATBC 2003 study included) and the analysis re-run. We postulated a dummy trial with a vitamin E dosage of 500 IU daily over a period of 10 years in a cohort with a low rate of underlying mortality (1%) in which no difference in mortality was observed between the vitamin E and the placebo groups. The reason chosen for these dummy variables was to simulate a trial that would indicate independently that vitamin E at a commonly available commercial dose (500 IU) taken daily over an extensive period (10 years) in large population was demonstrably safe. The cohort size chosen was equal to the total numbers in the vitamin E (*n* = 97,523) and placebo (*n* = 73,721) arms given in the low bias section of A11.

The ATBC study was conducted as a factorial study and data was available for five time periods - the study period, follow up at 3, 6, 8, and 18 years [7, 8]. The 8 year period was used in the meta-analysis published in 2012; the 18 year post-trial data was published in 2014 [9]. A new series of meta-analyses were run substituting the dataset from each of these time periods using both ‘inside the table’ and ‘at the margins’ data into the original A11 dataset. Simple factorial trials have four arms Active X and Active Y (A); placebo X and active Y (B); active X and placebo Y (C); placebo X and placebo Y (D) and the analysis is classically presented in a 2 × 2 table. In assessing the effect of a single substance the data can be analysed in two ways, 1) “inside the table” analysis looks at the effect of one substance alone versus placebo (A compared to D or B compared to D); and 2) “at the margins” analysis looks at the total use of specific substance versus the total non-use of that substance (A + B compared to C + D or A + C compared to B + D) [10]. See Table 1.

**Table 1:**
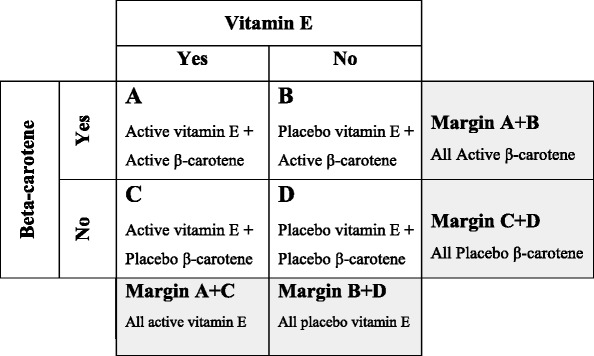
“At the margins” (4 cell) and “inside the cell” (2 cell) factorial design analysis

Legend: A simple factorial design (2 × 2) has 4 separate treatment arms represented by the 4 cells inside the Table (A, B, C and D). In the ATBC study [7] the four treatments were: cell A - active vitamin E with active β-carotene; cell B - active β-carotene with placebo vitamin E; cell C - active vitamin E with placebo β-carotene; and cell D - placebo vitamin E with placebo β-carotene. An ‘inside the table’ analysis uses two cells and compares active vitamin E alone (cell C) to all placebo (cell D) or compares active vitamin E alone (cell C) to active β-carotene alone (cell B); an ‘at the margins’ analysis uses 4 cells and compares all active vitamin E against and all placebo vitamin E (margin cell A + C vs margin cell B + D). The analysis used in the Cochrane review [3] uses 3 cells and compares all active vitamin E (margin cell A + C) to placebo (cell D)

## Results

The result of the replicated A11 analysis is given in Table 2 and this became the starting basis for all further meta-analyses. These results are an exact replica of the Cochrane meta-analysis [3] and demonstrate a risk ratio of 1.03 (95%CI 1.00 to 1.05; p˂0.04) for vitamin E supplementation in the low risk of bias subgroup. The associated forest plot can be found in Additional file 1: Fig. S1.

**Table 2:** Sensitivity analyses of Risk Ratio (RR) low risk of bias data subset by a) removing ATBC study, and b) adding fictitious dataset to original low risk of bias trials

Analysis	Vitamin E n/N^a^	Control n/N	ATBC Weighting	Low bias Subgroup RR	95% CIs	*P* value	Figure
Per Bjelakovic A11	11,689/97523	7561/73721	42.6	1.03	1.00–1.05	0.04	S1
Per Bjelakovic A11 minus ATBC 2003Low	6256/82959	4956/66434	0.0	1.01	0.98–1.05	0.44	S2
Per Bjelakovic A11 plus Fictitious Trial	12,664/195046	8298/147442	40.0	1.03	1.00–1.05	0.04	S3

^a^n/N – number of deaths/total number of participants

The sensitivity analysis with the ABTC study removed from A11 shows the risk ratio in the low risk of bias subgroup changing from statistically significant to becoming statistically non-significant with a risk ratio 1.01 (95%CI 0.98–1.05; *p* = 0.44). See Table 2. The associated forest plot can be found in Additional file 1: Fig. S2.

Further sensitivity analysis was done with the addition to A11 of a large fictitious low risk trial which doubles the overall population sample size in the low risk of bias subgroup. The fictitious trial gets a weighting of 6.1% but the overall risk ratio of mortality in the low risk of bias subgroup was unchanged – 1.03 (95%CI 1.00–1.05, *p* = 0.04). See Table 2. The associated forest plot can be found in Additional file 1: Fig. S3.

In the original A11 analysis the ATBC study uses the 8 year post-trial follow-up dataset. The meta-analysis was rerun in the low risk of bias subgroup using 5 time periods for the ATBC study (the study period itself and 4 post-trial follow up periods of 3,6, 8 and 18 years) utilising both ‘inside the table’ (2 cell) and ‘at the margins’ (4 cell) analysis. None of these analyses were of statistical significance. The data used in these analyses and the resultant risk ratios are given in Table 3. All the forest plots for this series are given in Additional file 1: Figs. S4-S13.

**Table 3:** Risk ratio (RR) according to ATBC study period analysed using three methods - ‘inside the table’ (2 cell), ‘at the margins’ (4 cell) and Bjelakovic (3 cell) - utilising the Bjelakovic low risk of bias dataset (A11)

Trial Period	Active n/N^a^	Control n/N	ATBC Weighting	Low risk Bias Subset RR	95% CIs	*P* value	Figure
At the Margins Analysis (4 cell analysis)							
	All Vit E	All non-Vit E					
April 1985–April 1993^b^	1800/14564	1770/14569	21.5	1.01	0.98–1.05	0.34	S4
April 1985–April 1996	2993/14564	3019/14569	33.8	1.01	0.98–1.03	0.70	S5
April 1985–April 1999	4453/14564	4415/14569	46.2	1.01	0.99–1.04	0.37	S6
April 1985–April 2001	5433/14564	5398/14569	53.7	1.01	0.99–1.03	0.40	S7
April 1985–April 2011	10,182/14564	10,074/14569	81.7	1.01	1.00–1.03	0.11	S8
Inside the Table Analysis (2 cell analysis)							
	Vit E Alone	Placebo					
April 1985–April 1993^a^	868/7286	851/7287	11.6	1.01	0.98–1.05	0.38	S9
April 1985–April 1996	1434/7286	1449/7287	19.5	1.01	0.98–1.04	0.59	S10
April 1985–April 1999	2167/7286	2117/7287	29.0	1.02	0.99–1.05	0.24	S11
April 1985–April 2001	2671/7286	2605/7287	35.7	1.02	0.99–1.05	0.18	S12
April 1985–April 2011	5065/7286	5022/7287	68.8	1.01	0.99–1.03	0.28	S13
Bjelakovic (3 cell analysis)							
	All Vit E	Placebo					
April 1985–April 1993^a^	1800/14564	851/7287	15.0	1.02	0.99–1.06	0.19	S14
April 1985–April 1996	2993/14564	1449/7287	24.7	1.02	0.99–1.05	0.20	S15
April 1985–April 1999	4453/14564	2117/7287	35.4	1.03	1.00–1.06	0.04	S16
April 1985–April 2001^c^	5433/14564	2605/7287	42.6	1.03	1.00–1.05	0.04	S1
April 1985–April 2011	10,182/14564	5022/7287	74.6	1.01	1.00–1.03	0.09	S17

^a^n/N – number of deaths/total number of participants, ^b^Trial period, ^c^Dataset used by Bjelakovic et al. [3] analysis

In constructing the ‘inside the table’ and ‘at the margins’ datasets it became apparent that the data used for the ATBC study in A11 was neither ‘inside the table’ (2 cell) nor ‘at the margins’ (4 cell) but was a 3 cell analysis (vitamin E alone and vitamin E plus beta-carotene versus the placebo only alone arm). Analysis was therefore repeated for the other ATBC study periods using this 3 cell method in the low risk of bias subgroup. A statistically significant risk ratio occurred only for the 6 year (1985 to 1999) post-trial period in addition to the 8 year (1985 to 2001) post-trial period used in the Cochrane meta-analysis [3]. The datasets used in these analyses and the resultant risk ratios are given in Table 3. All the forest plots for this series are given in Additional file 1: Figs. S14-S17.

## Discussion

Examination of the 2012 Cochrane review by Bjelakovic et al. raises several major issues which have broader implications for all similar meta-analyses; these are sensitivity, internal validity and external validity.

A cursory examination of A11 shows the overwhelming impact of the ATBC study on the meta-analysis outcome due to its high weighting (42.6%). The removal of the ATBC study from A11 renders the low bias subgroup analysis statistically non-significant. It could be argued that this is because of a diminution in study power; however, even the addition of a fictitious low-event trial doubling the baseline sample size had little impact on the overall risk ratio. The reason for this is that the weighting of a clinical trial’s contribution to a meta-analysis is based on the inverse of the variance observed and variance is determined by the number of events e.g. deaths. This means that studies with low numbers of events, i.e. low mortality rates, have little or no impact on the analysis as our fictitious study demonstrated. Yet these studies logically contribute to an understanding about the safety of the preparation under review. We hypothesised that a study the size of our fictitious study should provide evidence of safety at the dosage used for the specified duration in the study population. In the Cochrane meta-analysis [3] it was stated that 481 antioxidant trials (approximately 42,000 persons) were found that had zero mortality in both the experimental and control groups. These trials were excluded from the meta-analyses on the grounds that exploratory analyses by adding an imagined trial with 1 death and 21,000 participants in each intervention group had no impact on the outcome (this would be a mortality rate of 0.0047%). It was concluded that “the influence of zero events trials on the our final result was not noticeable” [3]. However, exclusion of low mortality data that implicitly illustrate safety is a major failing that risks substantial bias and undermines the conclusions of the current meta-analysis. Current meta-analysis models are designed to assess efficacy where zero outcome in a clinical trial generally equals a poor response, while in the assessment of safety a zero outcome is the preferred response. One commentator noted about the issue of weighting that “when dealing with efficacy, that is not a problem, as everyone has an outcome, and the question is what the level or the direction of the effect is, but it is [a problem] when you’re looking at safety: zero outcomes – no heart attacks for instance – is important information” [11]. While studies that have high underlying mortality rates may *individually* make important contributions to our understanding of the effect of an agent on mortality in a specific clinical situation at a specific dose for a specific duration; their inclusion into mathematical models along with studies that have no or low mortality gives them a high weighting potentially distorting their relative contribution to external validity. Unfortunately, current meta-analysis methods do not allow for studies with low (or no) mortality to be incorporated in a meaningful way.

The choice of datasets from any included studies is obviously important for internal validity. The ATBC study was a 2 × 2 factorial clinical trial of approximately 30,000 Finnish male smokers at high risk of cancer assigned to one of four arms: a low dose of synthetic vitamin E (50 mg), a moderate dose of synthetic β-carotene (20 mg), a combined arm of vitamin E and β-carotene or a placebo [7]. The most appropriate type of analysis would be to compare the vitamin E arm alone to the placebo alone arm i.e. ‘inside the table’ (2 cell) or by comparing all vitamin E to all non-vitamin E i.e. ‘at the margins’ (4 cells). The 3 cell method used in the Cochrane meta-analysis (1) in A11 compared both vitamin E arms (alone and with β-carotene) to placebo only. This analysis is confounded by the mortality associated with β-carotene and cannot be used to determine mortality associated with vitamin E alone. Utilising data from the 2 cell ‘in-table’ or 4 cell ‘at the margins’ is the only correct method for determining mortality associated with alpha-tocopherol and doing so in this case renders the risk ratio statistically non-significant.

Another important question relating to data selection is the use of post-trial follow-up data. The use of extended post-trial data can have a significant impact on the weighting of a study to the overall meta-analysis because the longer the study the more deaths and the greater the weighting, even if there were no changes in the risk ratio between the active and placebo group. The weighting for the ATBC study using the trial period only (1985–1993: ‘at the margins’) was 21.5% while use of the ATBC study 18 year post-trial data (1985–2011: ‘at the margins’) increased the weighting to 81.7% (see Table 3). Of note is that in A11, 28 of the 46 included studies in the low bias group and 13 of the 18 included studies in the high bias group have a weighting of less than 0.1%.

This example of disproportionate weighting raises the important aspect of evaluating the external validity of a meta-analysis as one would with a clinical trial. The question of external validity should be a critical concern and is central to the intent of any meta-analysis. An investigator needs to determine if the available datasets have the clinical and methodological homogeneity required for a pooled estimate. In this case the vitamin E studies used in the Cochrane 2012 meta-analysis have considerable clinical and study design heterogeneity. There are pooled studies on: 1) healthy individuals and individuals with chronic diseases (e.g. cancer, Alzheimer’s disease, Parkinson’s disease, renal disease); 2) different gender mixes (males only, females only, mixed); 3) smokers only, non-smokers or mixed (where there is likely to be differences in vitamin E pharmacokinetics [12, 13]; 4) different forms of vitamin E (natural and synthetic – known to have different bioavailability and pharmacokinetics [14]; 5) different doses from as little as 33 IU daily to 5000 IU daily; 6) different intervention time periods ranging from 6 weeks to 5 years; and 7) the use of vitamin E alone or vitamin E together with different concomitant nutrients and/or pharmaceutical drugs. The 2012 Bjelakovic meta-analysis concluded, incorrectly in our opinion, that there was an increased risk of mortality with vitamin E supplementation – i.e. 1.03 (95%CI 1.00–1.05, *p* = 0.04). A simple question would be, to whom would this increase in risk apply to? With beta-carotene, data from the ATBC [8] and CARET [15] trials made it clear that male smokers and persons exposed to asbestos were at higher risk. In the case of vitamin E with the existing meta-analysis it would be difficult to define a specific at-risk group given the considerable heterogeneity of the pooled studies and the disproportionate weighting of a trial of male smokers. Unfortunately, the conclusions reached of increased mortality for vitamin E supplementation in the Cochrane 2012 meta-analysis lack any of the precision required in toxicology and leave open the interpretation that any form of vitamin E, taken at any dose, for any duration, by anybody, at any age is potentially fatal.

Bailar notes that a rigorous technical review of a meta-analysis requires the reviewer to identify, re-abstract, and interpret a fair sample of the original papers [16]. He considers very few editors and reviewers will do this and suggests that this may be one reason why there are so many poor quality meta-analyses in the literature. We suggest that journals require authors of factorial trials to publish results of all treatment arms separately so that both ‘inside the table’ and ‘at the margins’ analysis be performed if deemed necessary. In addition, it is not readily evident from reading the methodological sections of some papers that they are part of a broader factorial study as some factorial trials are very complex in their design. In some published meta-analyses it is unclear what datasets are actually used because the number of events and totals are not specified on the forest plots. Perhaps there needs to be an adjustment to the PRISMA guidelines for meta-analysis, not just on the inclusion and exclusion of the trials, but inclusion and exclusion of the trial datasets per se used in the meta-analysis and mandatory reporting of the actual event and non-event datasets in forest plots or tables.

## Conclusions

We believe there are several methodological issues in the meta-analysis of vitamin E safety in the 2012 Cochrane Review that negate the original findings. These include the technique used to analyse data in a key factorial paper, disproportionate weighting in the same trial with use of extended post-trial data, the inability of meta-analysis to incorporate low or no mortality datasets and overall poor external validity given the disparate nature of the trials included. We would strongly argue that new models need to be developed that appropriately weight studies with low to no mortality outcomes as failure to account for these datasets obscures potentially important data. Until then we may need to rely on conventional narrative systematic literature synthesis in the assessment of safety or contain our results to specific sub-populations where more conclusive results can be determined.

Currently it is not always possible for readers to query the selection of a study clinical trial dataset used within a meta-analysis and we call on changes to be made to the PRISMA guidelines to ensure appropriate data transparency in any meta-analysis.

## Additional file

Additional file 1
**Fig. S1**. A11 - ATBC 85–01 as per Bjelakovic 2012. **Fig. S2**. A11 - Minus ATBC 85–01. **Fig. S3**. A11 - Plus Fictitious Trial. **Fig. S4**. A11 - ATBC 85–93 - At the Margins (4 cell) Analysis. **Fig. S5**. A11 - ATBC 85–96 - At the Margins (4 cell) Analysis. **Fig. S6**. A11 - ATBC 85–99 - At the Margins (4 cell) Analysis. **Fig. S7**. A11 - ATBC 85–01 - At the Margins (4 cell) Analysis. **Fig. S8**. A11 - ATBC 85–11 - At the Margins (4 cell) Analysis. **Fig. S9.** A11 - ATBC 85–93 - Inside the Table (2 cell) Analysis. **Fig. S10**. A11 - ATBC 85–96 - Inside the Table (2 cell) Analysis. **Fig. S11**. A11 - ATBC 85–99 - Inside the Table (2 cell) Analysis. **Fig. S12**. A11 - ATBC 85–01 - Inside the Table (2 cell) Analysis. **Fig. S13**. A11 - ATBC 85–11 - Inside the Table (2 cell) Analysis. **Fig. S14**. A11 - ATBC 85–93 - Bjelakovic (3 cell) Analysis. **Fig. S15**. A11 - ATBC 85–96 - Bjelakovic (3 cell) Analysis. **Fig. S16**. A11 - ATBC 85–99 - Bjelakovic (3 cell) Analysis. **Fig. S17**. A11 - ATBC 85–11 - Bjelakovic (3 cell) Analysis. (PDF 12335 kb)
