# Supplementary material for: Validity of a Cochrane Systematic Review and meta-analysis for determining the safety of vitamin E
Source: BMC Complement Altern Med. 2017 Aug 16;17:408. doi: 10.1186/s12906-017-1906-x (PMC5565069; doi:10.1186/s12906-017-1906-x)

Figure S1 - A11 - ATBC 85-01 as per Bjelakovic 2012 (3 cell)

Study or Subgroup	Vitamin E Events	Vitamin E Total	Control Events	Control Total	Weight	Risk Ratio M-H, Random, 95% CI
**1.1.1 Low Bias Risk**						
Allsup 2004Low	4	81	4	83	0.0%	1.02 [0.27, 3.96]
ALSRT 2001Low	34	144	35	144	0.4%	0.97 [0.64, 1.47]
AMDS 1996Low	2	39	2	32	0.0%	0.82 [0.12, 5.50]
AREDS 2001Low	251	2370	240	2387	2.1%	1.05 [0.89, 1.25]
ASAP 2003Low	4	260	1	130	0.0%	2.00 [0.23, 17.71]
ATBC EB 85-01 03Low	5433	14564	2605	7287	42.6%	1.04 [1.01, 1.08]
CHAOS 1996Low	68	1035	52	967	0.5%	1.22 [0.86, 1.73]
Collins 2002Low	1	26	1	26	0.0%	1.00 [0.07, 15.15]
DATATOP 2005Low	154	399	142	401	1.8%	1.09 [0.91, 1.31]
DATOR 2004 Low	1	12	0	12	0.0%	3.00 [0.13, 67.06]
Garbagnatti 2009Low	1	34	3	38	0.0%	0.37 [0.04, 3.41]
Graat 2002Low	3	499	5	153	0.0%	0.18 [0.04, 0.76]
Graf 2005Low	31	83	28	77	0.4%	1.03 [0.68, 1.54]
Grieger 2009Low	3	58	4	57	0.0%	0.74 [0.17, 3.15]
HATS 2001Low	1	84	1	76	0.0%	0.90 [0.06, 14.22]
HOPE_TOO 2005Low	799	4761	801	4780	7.4%	1.00 [0.92, 1.10]
HPS 2002Low	1446	10269	1389	10267	12.7%	1.04 [0.97, 1.11]
ICARE 2008Low	11	726	12	708	0.1%	0.89 [0.40, 2.01]
Jacobson 2000Low	0	57	1	55	0.0%	0.32 [0.01, 7.74]
LAST 2004Low	0	30	2	31	0.0%	0.21 [0.01, 4.13]
Liu 2007Low	96	379	97	384	1.0%	1.00 [0.79, 1.28]
MAVET 2006Low	9	205	17	204	0.1%	0.53 [0.24, 1.15]
MAVIS 2005Low	8	456	4	454	0.0%	1.99 [0.60, 6.57]
Meydani 2004Low	39	311	44	306	0.4%	0.87 [0.58, 1.30]
Mezey 2004Low	4	25	5	26	0.0%	0.83 [0.25, 2.75]
MINVITAOX 1999Low	100	361	51	182	0.7%	0.99 [0.74, 1.32]
Mooney 2005Low	1	142	0	142	0.0%	3.00 [0.12, 73.03]
NIT2 1993Low	157	1657	167	1661	1.4%	0.94 [0.77, 1.16]
PHS 2008Low	857	7329	405	3653	4.8%	1.05 [0.94, 1.18]
Pike 1995Low	1	24	0	23	0.0%	2.88 [0.12, 67.29]
Plummer 2007Low	16	990	11	990	0.1%	1.45 [0.68, 3.12]
PPS 1994Low	15	433	14	214	0.1%	0.53 [0.26, 1.08]
Prince 2003Low	1	29	0	32	0.0%	3.30 [0.14, 77.95]
REACT 2002Low	9	149	3	148	0.0%	2.98 [0.82, 10.79]
SELECT 2009Low	717	17767	382	8856	4.0%	0.94 [0.83, 1.06]
SPACE 2000Low	31	97	29	99	0.3%	1.09 [0.72, 1.66]
SUVIMAX 2010Low	156	6481	178	6536	1.3%	0.88 [0.71, 1.09]
Tam 2005Low	1	20	1	19	0.0%	0.95 [0.06, 14.13]
VEAPS 2002Low	2	177	1	176	0.0%	1.99 [0.18, 21.73]
VECAT 2004Low	20	595	11	598	0.1%	1.83 [0.88, 3.78]
WACS 2007Low	502	4083	124	1022	1.7%	1.01 [0.84, 1.22]
WAVE 2002Low	16	212	6	211	0.1%	2.65 [1.06, 6.65]
White 2002Low	1	50	1	50	0.0%	1.00 [0.06, 15.55]
WHS 2005Low	681	19937	681	19939	5.4%	1.00 [0.90, 1.11]
Witte 2005Low	1	16	1	16	0.0%	1.00 [0.07, 14.64]
Wluka 2002Low	1	67	0	69	0.0%	3.09 [0.13, 74.50]
**Subtotal (95% CI)**		**97523**		**73721**	**89.9%**	**1.03 [1.00, 1.05]**
Total events	11689		7561			
Heterogeneity: Tau² = 0.00; Chi² = 37.62, df = 45 (P = 0.77); I² = 0%						
Test for overall effect: Z = 2.11 (P = 0.04)						
**1.1.2 High Bias Risk**						
H ADCS 1 1997	12	85	12	84	0.1%	0.99 [0.47, 2.07]
H ADCS 2 2005	5	257	5	259	0.0%	1.01 [0.30, 3.44]
H Bonelli 1998	1	147	0	157	0.0%	3.20 [0.13, 78.00]
H Chandra 1992	0	48	2	48	0.0%	0.20 [0.01, 4.06]
H CTNS 2008	77	510	81	510	0.7%	0.95 [0.71, 1.27]
H de la Maza 1995	5	37	4	37	0.0%	1.25 [0.36, 4.29]
H de Waart 2001	0	109	1	109	0.0%	0.33 [0.01, 8.09]
H Gillilan 1977	2	26	2	26	0.0%	1.00 [0.15, 6.57]
H Girodon 1997	12	41	7	20	0.1%	0.84 [0.39, 1.79]
H GISSI 1999	488	5660	529	5664	4.3%	0.92 [0.82, 1.04]
H McKeown-Eys 1988	4	96	3	89	0.0%	1.24 [0.28, 5.37]
H NIT1 1993	1018	14792	280	3698	3.7%	0.91 [0.80, 1.03]
H Penn 1991	1	15	0	15	0.0%	3.00 [0.13, 68.26]
H PPP 2001	72	2231	68	2264	0.6%	1.07 [0.78, 1.49]
H SIT 2001	38	1706	43	1705	0.3%	0.88 [0.57, 1.36]
H Stevic 2001	3	16	6	12	0.0%	0.38 [0.12, 1.20]
H Takagi 2003	10	51	16	42	0.1%	0.51 [0.26, 1.01]
H Takmatsu 1995	1	74	0	73	0.0%	2.96 [0.12, 71.50]
**Subtotal (95% CI)**		**25901**		**14812**	**10.1%**	**0.92 [0.85, 0.99]**
Total events	1749		1059			
Heterogeneity: Tau² = 0.00; Chi² = 9.65, df = 17 (P = 0.92); I² = 0%						
Test for overall effect: Z = 2.18 (P = 0.03)						
**Total (95% CI)**		**123424**		**88533**	**100.0%**	**1.02 [0.99, 1.04]**
Total events	13438		8620			
Heterogeneity: Tau² = 0.00; Chi² = 54.80, df = 63 (P = 0.76); I² = 0%						
Test for overall effect: Z = 1.30 (P = 0.19)						
Test for subgroup differences: Chi² = 7.49, df = 1 (P = 0.006), I² = 86.6%						

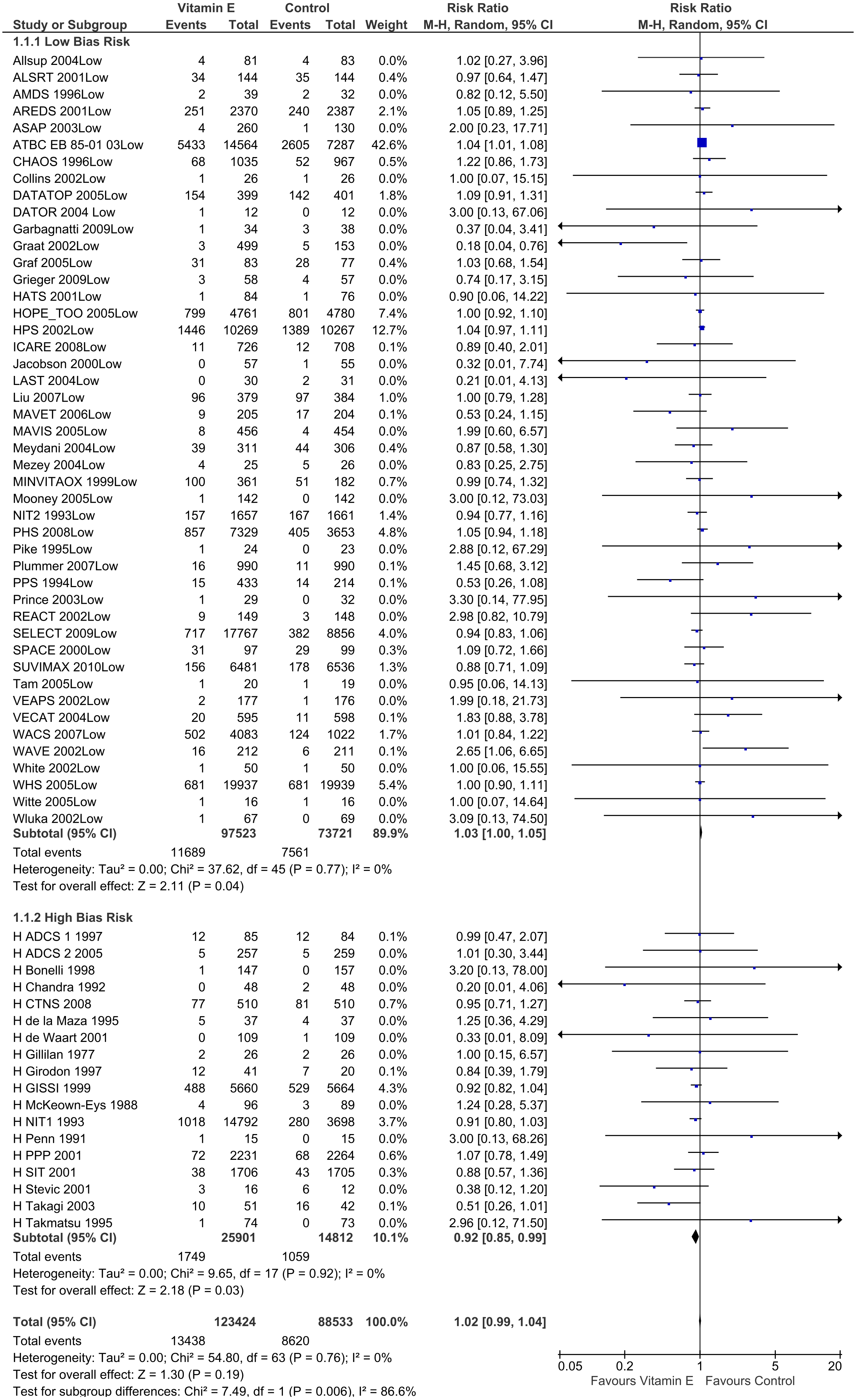

Figure S2 - A11 - Minus ATBC 85-01

Study or Subgroup	Vitamin E Events	Vitamin E Total	Control Events	Control Total	Weight	Risk Ratio M-H, Random, 95% CI
**1.2.1 Low Bias Risk**						
Allsup 2004Low	4	81	4	83	0.1%	1.02 [0.27, 3.96]
ALSRT 2001Low	34	144	35	144	0.6%	0.97 [0.64, 1.47]
AMDS 1996Low	2	39	2	32	0.0%	0.82 [0.12, 5.50]
AREDS 2001Low	251	2370	240	2387	3.7%	1.05 [0.89, 1.25]
ASAP 2003Low	4	260	1	130	0.0%	2.00 [0.23, 17.71]
CHAOS 1996Low	68	1035	52	967	0.8%	1.22 [0.86, 1.73]
Collins 2002Low	1	26	1	26	0.0%	1.00 [0.07, 15.15]
DATATOP 2005Low	154	399	142	401	3.2%	1.09 [0.91, 1.31]
DATOR 2004 Low	1	12	0	12	0.0%	3.00 [0.13, 67.06]
Garbagnatti 2009Low	1	34	3	38	0.0%	0.37 [0.04, 3.41]
Graat 2002Low	3	499	5	153	0.1%	0.18 [0.04, 0.76]
Graf 2005Low	31	83	28	77	0.6%	1.03 [0.68, 1.54]
Grieger 2009Low	3	58	4	57	0.0%	0.74 [0.17, 3.15]
HATS 2001Low	1	84	1	76	0.0%	0.90 [0.06, 14.22]
HOPE_TOO 2005Low	799	4761	801	4780	12.9%	1.00 [0.92, 1.10]
HPS 2002Low	1446	10269	1389	10267	22.1%	1.04 [0.97, 1.11]
ICARE 2008Low	11	726	12	708	0.2%	0.89 [0.40, 2.01]
Jacobson 2000Low	0	57	1	55	0.0%	0.32 [0.01, 7.74]
LAST 2004Low	0	30	2	31	0.0%	0.21 [0.01, 4.13]
Liu 2007Low	96	379	97	384	1.7%	1.00 [0.79, 1.28]
MAVET 2006Low	9	205	17	204	0.2%	0.53 [0.24, 1.15]
MAVIS 2005Low	8	456	4	454	0.1%	1.99 [0.60, 6.57]
Meydani 2004Low	39	311	44	306	0.6%	0.87 [0.58, 1.30]
Mezey 2004Low	4	25	5	26	0.1%	0.83 [0.25, 2.75]
MINVITAOX 1999Low	100	361	51	182	1.3%	0.99 [0.74, 1.32]
Mooney 2005Low	1	142	0	142	0.0%	3.00 [0.12, 73.03]
NIT2 1993Low	157	1657	167	1661	2.4%	0.94 [0.77, 1.16]
PHS 2008Low	857	7329	405	3653	8.3%	1.05 [0.94, 1.18]
Pike 1995Low	1	24	0	23	0.0%	2.88 [0.12, 67.29]
Plummer 2007Low	16	990	11	990	0.2%	1.45 [0.68, 3.12]
PPS 1994Low	15	433	14	214	0.2%	0.53 [0.26, 1.08]
Prince 2003Low	1	29	0	32	0.0%	3.30 [0.14, 77.95]
REACT 2002Low	9	149	3	148	0.1%	2.98 [0.82, 10.79]
SELECT 2009Low	717	17767	382	8856	7.0%	0.94 [0.83, 1.06]
SPACE 2000Low	31	97	29	99	0.6%	1.09 [0.72, 1.66]
SUVIMAX 2010Low	156	6481	178	6536	2.3%	0.88 [0.71, 1.09]
Tam 2005Low	1	20	1	19	0.0%	0.95 [0.06, 14.13]
VEAPS 2002Low	2	177	1	176	0.0%	1.99 [0.18, 21.73]
VECAT 2004Low	20	595	11	598	0.2%	1.83 [0.88, 3.78]
WACS 2007Low	502	4083	124	1022	3.0%	1.01 [0.84, 1.22]
WAVE 2002Low	16	212	6	211	0.1%	2.65 [1.06, 6.65]
White 2002Low	1	50	1	50	0.0%	1.00 [0.06, 15.55]
WHS 2005Low	681	19937	681	19939	9.5%	1.00 [0.90, 1.11]
Witte 2005Low	1	16	1	16	0.0%	1.00 [0.07, 14.64]
Wluka 2002Low	1	67	0	69	0.0%	3.09 [0.13, 74.50]
**Subtotal (95% CI)**		**82959**		**66434**	**82.4%**	**1.01 [0.98, 1.05]**
Total events	6256		4956			
Heterogeneity: Tau² = 0.00; Chi² = 36.39, df = 44 (P = 0.79); I² = 0%						
Test for overall effect: Z = 0.78 (P = 0.44)						
**1.2.2 High Bias Risk**						
H ADCS 1 1997	12	85	12	84	0.2%	0.99 [0.47, 2.07]
H ADCS 2 2005	5	257	5	259	0.1%	1.01 [0.30, 3.44]
H Bonelli 1998	1	147	0	157	0.0%	3.20 [0.13, 78.00]
H Chandra 1992	0	48	2	48	0.0%	0.20 [0.01, 4.06]
H CTNS 2008	77	510	81	510	1.3%	0.95 [0.71, 1.27]
H de la Maza 1995	5	37	4	37	0.1%	1.25 [0.36, 4.29]
H de Waart 2001	0	109	1	109	0.0%	0.33 [0.01, 8.09]
H Gillilan 1977	2	26	2	26	0.0%	1.00 [0.15, 6.57]
H Girodon 1997	12	41	7	20	0.2%	0.84 [0.39, 1.79]
H GISSI 1999	488	5660	529	5664	7.5%	0.92 [0.82, 1.04]
H McKeown-Eys 1988	4	96	3	89	0.0%	1.24 [0.28, 5.37]
H NIT1 1993	1018	14792	280	3698	6.4%	0.91 [0.80, 1.03]
H Penn 1991	1	15	0	15	0.0%	3.00 [0.13, 68.26]
H PPP 2001	72	2231	68	2264	1.0%	1.07 [0.78, 1.49]
H SIT 2001	38	1706	43	1705	0.6%	0.88 [0.57, 1.36]
H Stevic 2001	3	16	6	12	0.1%	0.38 [0.12, 1.20]
H Takagi 2003	10	51	16	42	0.2%	0.51 [0.26, 1.01]
H Takmatsu 1995	1	74	0	73	0.0%	2.96 [0.12, 71.50]
**Subtotal (95% CI)**		**25901**		**14812**	**17.6%**	**0.92 [0.85, 0.99]**
Total events	1749		1059			
Heterogeneity: Tau² = 0.00; Chi² = 9.65, df = 17 (P = 0.92); I² = 0%						
Test for overall effect: Z = 2.18 (P = 0.03)						
**Total (95% CI)**		**108860**		**81246**	**100.0%**	**1.00 [0.97, 1.03]**
Total events	8005		6015			
Heterogeneity: Tau² = 0.00; Chi² = 51.35, df = 62 (P = 0.83); I² = 0%						
Test for overall effect: Z = 0.21 (P = 0.84)						
Test for subgroup differences: Chi² = 5.32, df = 1 (P = 0.02), I² = 81.2%						

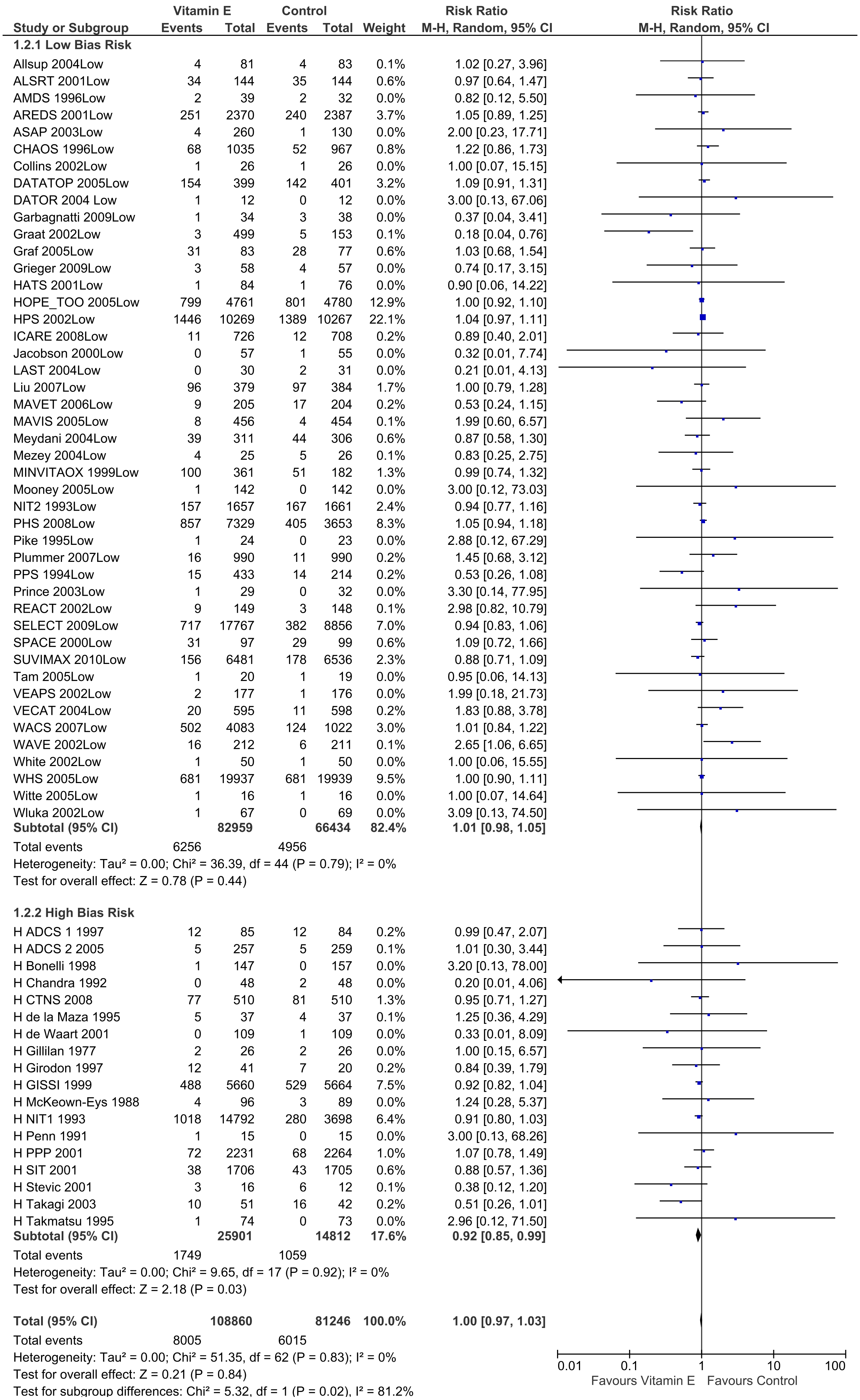

Figure S3 - A11 - Plus Fictitious Trial

Study or Subgroup	Vitamin E Events	Vitamin E Total	Control Events	Control Total	Weight	Risk Ratio M-H, Random, 95% CI
**1.3.1 Low Bias Risk**						
*Fictitious	975	97523	737	73721	6.1%	1.00 [0.91, 1.10]
Allsup 2004Low	4	81	4	83	0.0%	1.02 [0.27, 3.96]
ALSRT 2001Low	34	144	35	144	0.3%	0.97 [0.64, 1.47]
AMDS 1996Low	2	39	2	32	0.0%	0.82 [0.12, 5.50]
AREDS 2001Low	251	2370	240	2387	2.0%	1.05 [0.89, 1.25]
ASAP 2003Low	4	260	1	130	0.0%	2.00 [0.23, 17.71]
ATBC EB 85-01 03Low	5433	14564	2605	7287	40.0%	1.04 [1.01, 1.08]
CHAOS 1996Low	68	1035	52	967	0.5%	1.22 [0.86, 1.73]
Collins 2002Low	1	26	1	26	0.0%	1.00 [0.07, 15.15]
DATATOP 2005Low	154	399	142	401	1.7%	1.09 [0.91, 1.31]
DATOR 2004 Low	1	12	0	12	0.0%	3.00 [0.13, 67.06]
Garbagnatti 2009Low	1	34	3	38	0.0%	0.37 [0.04, 3.41]
Graat 2002Low	3	499	5	153	0.0%	0.18 [0.04, 0.76]
Graf 2005Low	31	83	28	77	0.3%	1.03 [0.68, 1.54]
Grieger 2009Low	3	58	4	57	0.0%	0.74 [0.17, 3.15]
HATS 2001Low	1	84	1	76	0.0%	0.90 [0.06, 14.22]
HOPE_TOO 2005Low	799	4761	801	4780	7.0%	1.00 [0.92, 1.10]
HPS 2002Low	1446	10269	1389	10267	11.9%	1.04 [0.97, 1.11]
ICARE 2008Low	11	726	12	708	0.1%	0.89 [0.40, 2.01]
Jacobson 2000Low	0	57	1	55	0.0%	0.32 [0.01, 7.74]
LAST 2004Low	0	30	2	31	0.0%	0.21 [0.01, 4.13]
Liu 2007Low	96	379	97	384	0.9%	1.00 [0.79, 1.28]
MAVET 2006Low	9	205	17	204	0.1%	0.53 [0.24, 1.15]
MAVIS 2005Low	8	456	4	454	0.0%	1.99 [0.60, 6.57]
Meydani 2004Low	39	311	44	306	0.3%	0.87 [0.58, 1.30]
Mezey 2004Low	4	25	5	26	0.0%	0.83 [0.25, 2.75]
MINVITAOX 1999Low	100	361	51	182	0.7%	0.99 [0.74, 1.32]
Mooney 2005Low	1	142	0	142	0.0%	3.00 [0.12, 73.03]
NIT2 1993Low	157	1657	167	1661	1.3%	0.94 [0.77, 1.16]
PHS 2008Low	857	7329	405	3653	4.5%	1.05 [0.94, 1.18]
Pike 1995Low	1	24	0	23	0.0%	2.88 [0.12, 67.29]
Plummer 2007Low	16	990	11	990	0.1%	1.45 [0.68, 3.12]
PPS 1994Low	15	433	14	214	0.1%	0.53 [0.26, 1.08]
Prince 2003Low	1	29	0	32	0.0%	3.30 [0.14, 77.95]
REACT 2002Low	9	149	3	148	0.0%	2.98 [0.82, 10.79]
SELECT 2009Low	717	17767	382	8856	3.8%	0.94 [0.83, 1.06]
SPACE 2000Low	31	97	29	99	0.3%	1.09 [0.72, 1.66]
SUVIMAX 2010Low	156	6481	178	6536	1.2%	0.88 [0.71, 1.09]
Tam 2005Low	1	20	1	19	0.0%	0.95 [0.06, 14.13]
VEAPS 2002Low	2	177	1	176	0.0%	1.99 [0.18, 21.73]
VECAT 2004Low	20	595	11	598	0.1%	1.83 [0.88, 3.78]
WACS 2007Low	502	4083	124	1022	1.6%	1.01 [0.84, 1.22]
WAVE 2002Low	16	212	6	211	0.1%	2.65 [1.06, 6.65]
White 2002Low	1	50	1	50	0.0%	1.00 [0.06, 15.55]
WHS 2005Low	681	19937	681	19939	5.1%	1.00 [0.90, 1.11]
Witte 2005Low	1	16	1	16	0.0%	1.00 [0.07, 14.64]
Wluka 2002Low	1	67	0	69	0.0%	3.09 [0.13, 74.50]
**Subtotal (95% CI)**		**195046**		**147442**	**90.5%**	**1.03 [1.00, 1.05]**
Total events	12664		8298			
Heterogeneity: Tau² = 0.00; Chi² = 37.93, df = 46 (P = 0.80); I² = 0%						
Test for overall effect: Z = 2.03 (P = 0.04)						
**1.3.2 High Bias Risk**						
H ADCS 1 1997	12	85	12	84	0.1%	0.99 [0.47, 2.07]
H ADCS 2 2005	5	257	5	259	0.0%	1.01 [0.30, 3.44]
H Bonelli 1998	1	147	0	157	0.0%	3.20 [0.13, 78.00]
H Chandra 1992	0	48	2	48	0.0%	0.20 [0.01, 4.06]
H CTNS 2008	77	510	81	510	0.7%	0.95 [0.71, 1.27]
H de la Maza 1995	5	37	4	37	0.0%	1.25 [0.36, 4.29]
H de Waart 2001	0	109	1	109	0.0%	0.33 [0.01, 8.09]
H Gillilan 1977	2	26	2	26	0.0%	1.00 [0.15, 6.57]
H Girodon 1997	12	41	7	20	0.1%	0.84 [0.39, 1.79]
H GISSI 1999	488	5660	529	5664	4.0%	0.92 [0.82, 1.04]
H McKeown-Eys 1988	4	96	3	89	0.0%	1.24 [0.28, 5.37]
H NIT1 1993	1018	14792	280	3698	3.4%	0.91 [0.80, 1.03]
H Penn 1991	1	15	0	15	0.0%	3.00 [0.13, 68.26]
H PPP 2001	72	2231	68	2264	0.5%	1.07 [0.78, 1.49]
H SIT 2001	38	1706	43	1705	0.3%	0.88 [0.57, 1.36]
H Stevic 2001	3	16	6	12	0.0%	0.38 [0.12, 1.20]
H Takagi 2003	10	51	16	42	0.1%	0.51 [0.26, 1.01]
H Takmatsu 1995	1	74	0	73	0.0%	2.96 [0.12, 71.50]
**Subtotal (95% CI)**		**25901**		**14812**	**9.5%**	**0.92 [0.85, 0.99]**
Total events	1749		1059			
Heterogeneity: Tau² = 0.00; Chi² = 9.65, df = 17 (P = 0.92); I² = 0%						
Test for overall effect: Z = 2.18 (P = 0.03)						
**Total (95% CI)**		**220947**		**162254**	**100.0%**	**1.02 [0.99, 1.04]**
Total events	14413		9357			
Heterogeneity: Tau² = 0.00; Chi² = 54.91, df = 64 (P = 0.78); I² = 0%						
Test for overall effect: Z = 1.26 (P = 0.21)						
Test for subgroup differences: Chi² = 7.29, df = 1 (P = 0.007), I² = 86.3%						

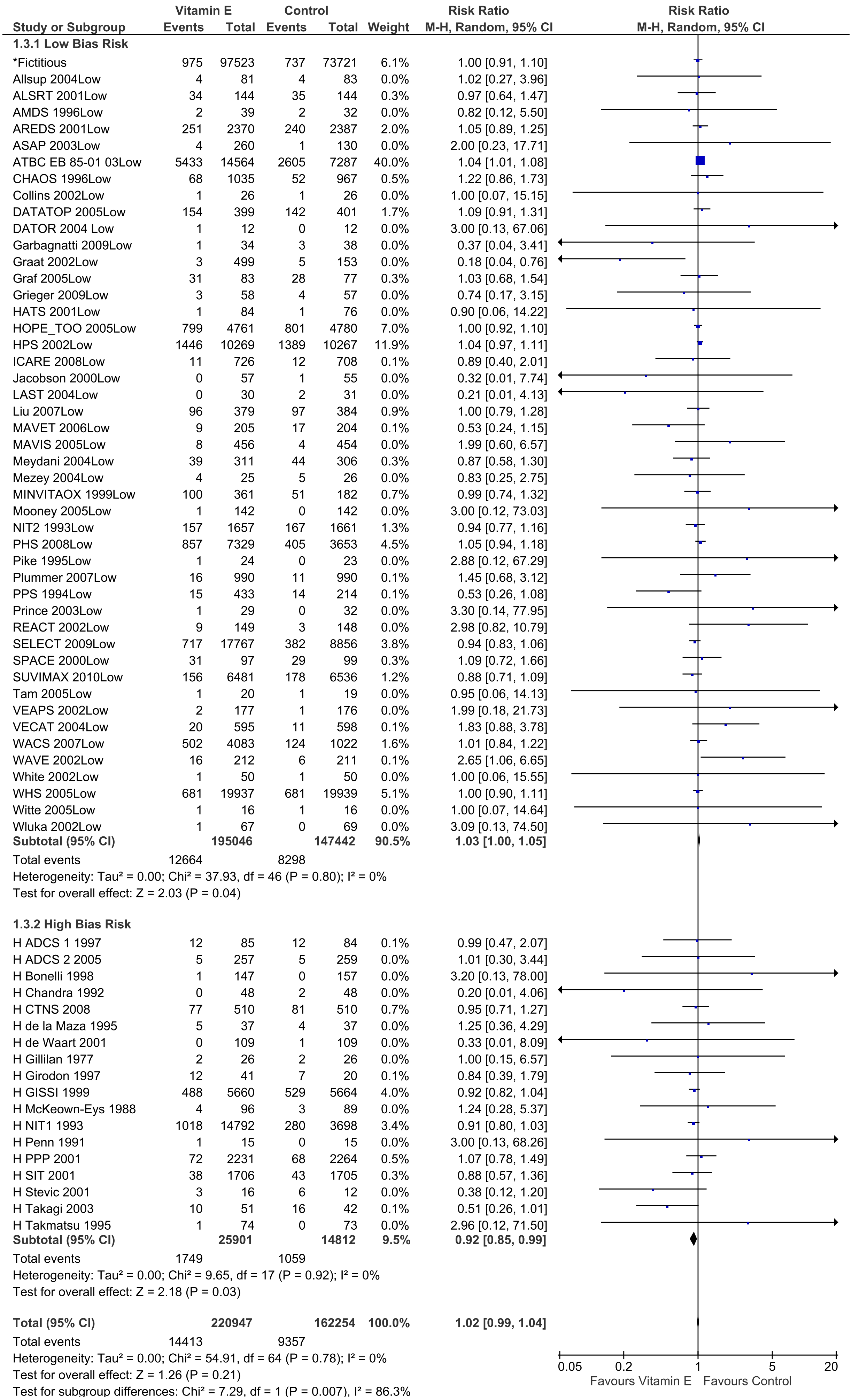

Figure S4 - A11 - ATBC 85-93 "At the Margins" (4 cell)

Study or Subgroup	Vitamin E Events	Vitamin E Total	Control Events	Control Total	Weight	Risk Ratio M-H, Random, 95% CI
**2.1.1 Low Bias Risk**						
Allsup 2004Low	4	81	4	83	0.0%	1.02 [0.27, 3.96]
ALSRT 2001Low	34	144	35	144	0.5%	0.97 [0.64, 1.47]
AMDS 1996Low	2	39	2	32	0.0%	0.82 [0.12, 5.50]
AREDS 2001Low	251	2370	240	2387	2.9%	1.05 [0.89, 1.25]
ASAP 2003Low	4	260	1	130	0.0%	2.00 [0.23, 17.71]
ATBC EB 85-93 03Low AM	1800	14564	1770	14569	21.5%	1.02 [0.96, 1.08]
CHAOS 1996Low	68	1035	52	967	0.7%	1.22 [0.86, 1.73]
Collins 2002Low	1	26	1	26	0.0%	1.00 [0.07, 15.15]
DATATOP 2005Low	154	399	142	401	2.5%	1.09 [0.91, 1.31]
DATOR 2004 Low	1	12	0	12	0.0%	3.00 [0.13, 67.06]
Garbagnatti 2009Low	1	34	3	38	0.0%	0.37 [0.04, 3.41]
Graat 2002Low	3	499	5	153	0.0%	0.18 [0.04, 0.76]
Graf 2005Low	31	83	28	77	0.5%	1.03 [0.68, 1.54]
Grieger 2009Low	3	58	4	57	0.0%	0.74 [0.17, 3.15]
HATS 2001Low	1	84	1	76	0.0%	0.90 [0.06, 14.22]
HOPE_TOO 2005Low	799	4761	801	4780	10.2%	1.00 [0.92, 1.10]
HPS 2002Low	1446	10269	1389	10267	17.4%	1.04 [0.97, 1.11]
ICARE 2008Low	11	726	12	708	0.1%	0.89 [0.40, 2.01]
Jacobson 2000Low	0	57	1	55	0.0%	0.32 [0.01, 7.74]
LAST 2004Low	0	30	2	31	0.0%	0.21 [0.01, 4.13]
Liu 2007Low	96	379	97	384	1.4%	1.00 [0.79, 1.28]
MAVET 2006Low	9	205	17	204	0.1%	0.53 [0.24, 1.15]
MAVIS 2005Low	8	456	4	454	0.1%	1.99 [0.60, 6.57]
Meydani 2004Low	39	311	44	306	0.5%	0.87 [0.58, 1.30]
Mezey 2004Low	4	25	5	26	0.1%	0.83 [0.25, 2.75]
MINVITAOX 1999Low	100	361	51	182	1.0%	0.99 [0.74, 1.32]
Mooney 2005Low	1	142	0	142	0.0%	3.00 [0.12, 73.03]
NIT2 1993Low	157	1657	167	1661	1.9%	0.94 [0.77, 1.16]
PHS 2008Low	857	7329	405	3653	6.5%	1.05 [0.94, 1.18]
Pike 1995Low	1	24	0	23	0.0%	2.88 [0.12, 67.29]
Plummer 2007Low	16	990	11	990	0.1%	1.45 [0.68, 3.12]
PPS 1994Low	15	433	14	214	0.2%	0.53 [0.26, 1.08]
Prince 2003Low	1	29	0	32	0.0%	3.30 [0.14, 77.95]
REACT 2002Low	9	149	3	148	0.0%	2.98 [0.82, 10.79]
SELECT 2009Low	717	17767	382	8856	5.5%	0.94 [0.83, 1.06]
SPACE 2000Low	31	97	29	99	0.5%	1.09 [0.72, 1.66]
SUVIMAX 2010Low	156	6481	178	6536	1.8%	0.88 [0.71, 1.09]
Tam 2005Low	1	20	1	19	0.0%	0.95 [0.06, 14.13]
VEAPS 2002Low	2	177	1	176	0.0%	1.99 [0.18, 21.73]
VECAT 2004Low	20	595	11	598	0.2%	1.83 [0.88, 3.78]
WACS 2007Low	502	4083	124	1022	2.4%	1.01 [0.84, 1.22]
WAVE 2002Low	16	212	6	211	0.1%	2.65 [1.06, 6.65]
White 2002Low	1	50	1	50	0.0%	1.00 [0.06, 15.55]
WHS 2005Low	681	19937	681	19939	7.4%	1.00 [0.90, 1.11]
Witte 2005Low	1	16	1	16	0.0%	1.00 [0.07, 14.64]
Wluka 2002Low	1	67	0	69	0.0%	3.09 [0.13, 74.50]
**Subtotal (95% CI)**		**97523**		**81003**	**86.2%**	**1.01 [0.98, 1.05]**
Total events	8056		6726			
Heterogeneity: Tau² = 0.00; Chi² = 36.40, df = 45 (P = 0.82); I² = 0%						
Test for overall effect: Z = 0.95 (P = 0.34)						
**2.1.2 High Bias Risk**						
H ADCS 1 1997	12	85	12	84	0.1%	0.99 [0.47, 2.07]
H ADCS 2 2005	5	257	5	259	0.1%	1.01 [0.30, 3.44]
H Bonelli 1998	1	147	0	157	0.0%	3.20 [0.13, 78.00]
H Chandra 1992	0	48	2	48	0.0%	0.20 [0.01, 4.06]
H CTNS 2008	77	510	81	510	1.0%	0.95 [0.71, 1.27]
H de la Maza 1995	5	37	4	37	0.1%	1.25 [0.36, 4.29]
H de Waart 2001	0	109	1	109	0.0%	0.33 [0.01, 8.09]
H Gillilan 1977	2	26	2	26	0.0%	1.00 [0.15, 6.57]
H Girodon 1997	12	41	7	20	0.1%	0.84 [0.39, 1.79]
H GISSI 1999	488	5660	529	5664	5.9%	0.92 [0.82, 1.04]
H McKeown-Eys 1988	4	96	3	89	0.0%	1.24 [0.28, 5.37]
H NIT1 1993	1018	14792	280	3698	5.0%	0.91 [0.80, 1.03]
H Penn 1991	1	15	0	15	0.0%	3.00 [0.13, 68.26]
H PPP 2001	72	2231	68	2264	0.8%	1.07 [0.78, 1.49]
H SIT 2001	38	1706	43	1705	0.4%	0.88 [0.57, 1.36]
H Stevic 2001	3	16	6	12	0.1%	0.38 [0.12, 1.20]
H Takagi 2003	10	51	16	42	0.2%	0.51 [0.26, 1.01]
H Takmatsu 1995	1	74	0	73	0.0%	2.96 [0.12, 71.50]
**Subtotal (95% CI)**		**25901**		**14812**	**13.8%**	**0.92 [0.85, 0.99]**
Total events	1749		1059			
Heterogeneity: Tau² = 0.00; Chi² = 9.65, df = 17 (P = 0.92); I² = 0%						
Test for overall effect: Z = 2.18 (P = 0.03)						
**Total (95% CI)**		**123424**		**95815**	**100.0%**	**1.00 [0.97, 1.03]**
Total events	9805		7785			
Heterogeneity: Tau² = 0.00; Chi² = 51.69, df = 63 (P = 0.84); I² = 0%						
Test for overall effect: Z = 0.07 (P = 0.94)						
Test for subgroup differences: Chi² = 5.65, df = 1 (P = 0.02), I² = 82.3%						

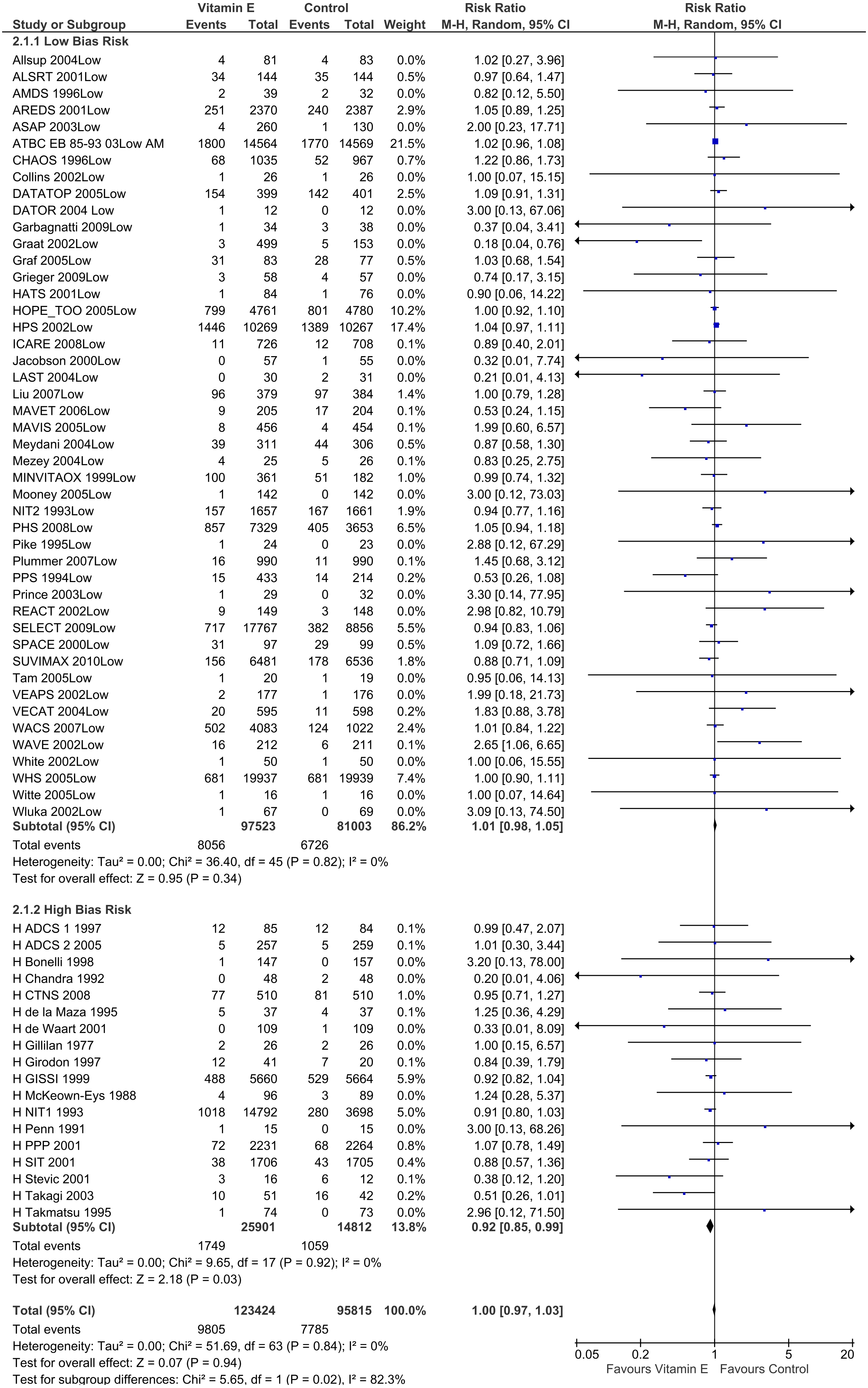

Figure S5 - A11 - ATBC 85-96 "At the Margins" (4 cell)

Study or Subgroup	Vitamin E Events	Vitamin E Total	Control Events	Control Total	Weight	Risk Ratio M-H, Random, 95% CI
**2.2.1 Low Bias Risk**						
Allsup 2004Low	4	81	4	83	0.0%	1.02 [0.27, 3.96]
ALSRT 2001Low	34	144	35	144	0.4%	0.97 [0.64, 1.47]
AMDS 1996Low	2	39	2	32	0.0%	0.82 [0.12, 5.50]
AREDS 2001Low	251	2370	240	2387	2.4%	1.05 [0.89, 1.25]
ASAP 2003Low	4	260	1	130	0.0%	2.00 [0.23, 17.71]
ATBC EB 85-96 03Low AM	2993	14564	3019	14569	33.8%	0.99 [0.95, 1.04]
CHAOS 1996Low	68	1035	52	967	0.6%	1.22 [0.86, 1.73]
Collins 2002Low	1	26	1	26	0.0%	1.00 [0.07, 15.15]
DATATOP 2005Low	154	399	142	401	2.1%	1.09 [0.91, 1.31]
DATOR 2004 Low	1	12	0	12	0.0%	3.00 [0.13, 67.06]
Garbagnatti 2009Low	1	34	3	38	0.0%	0.37 [0.04, 3.41]
Graat 2002Low	3	499	5	153	0.0%	0.18 [0.04, 0.76]
Graf 2005Low	31	83	28	77	0.4%	1.03 [0.68, 1.54]
Grieger 2009Low	3	58	4	57	0.0%	0.74 [0.17, 3.15]
HATS 2001Low	1	84	1	76	0.0%	0.90 [0.06, 14.22]
HOPE_TOO 2005Low	799	4761	801	4780	8.6%	1.00 [0.92, 1.10]
HPS 2002Low	1446	10269	1389	10267	14.7%	1.04 [0.97, 1.11]
ICARE 2008Low	11	726	12	708	0.1%	0.89 [0.40, 2.01]
Jacobson 2000Low	0	57	1	55	0.0%	0.32 [0.01, 7.74]
LAST 2004Low	0	30	2	31	0.0%	0.21 [0.01, 4.13]
Liu 2007Low	96	379	97	384	1.2%	1.00 [0.79, 1.28]
MAVET 2006Low	9	205	17	204	0.1%	0.53 [0.24, 1.15]
MAVIS 2005Low	8	456	4	454	0.0%	1.99 [0.60, 6.57]
Meydani 2004Low	39	311	44	306	0.4%	0.87 [0.58, 1.30]
Mezey 2004Low	4	25	5	26	0.0%	0.83 [0.25, 2.75]
MINVITAOX 1999Low	100	361	51	182	0.8%	0.99 [0.74, 1.32]
Mooney 2005Low	1	142	0	142	0.0%	3.00 [0.12, 73.03]
NIT2 1993Low	157	1657	167	1661	1.6%	0.94 [0.77, 1.16]
PHS 2008Low	857	7329	405	3653	5.5%	1.05 [0.94, 1.18]
Pike 1995Low	1	24	0	23	0.0%	2.88 [0.12, 67.29]
Plummer 2007Low	16	990	11	990	0.1%	1.45 [0.68, 3.12]
PPS 1994Low	15	433	14	214	0.1%	0.53 [0.26, 1.08]
Prince 2003Low	1	29	0	32	0.0%	3.30 [0.14, 77.95]
REACT 2002Low	9	149	3	148	0.0%	2.98 [0.82, 10.79]
SELECT 2009Low	717	17767	382	8856	4.6%	0.94 [0.83, 1.06]
SPACE 2000Low	31	97	29	99	0.4%	1.09 [0.72, 1.66]
SUVIMAX 2010Low	156	6481	178	6536	1.5%	0.88 [0.71, 1.09]
Tam 2005Low	1	20	1	19	0.0%	0.95 [0.06, 14.13]
VEAPS 2002Low	2	177	1	176	0.0%	1.99 [0.18, 21.73]
VECAT 2004Low	20	595	11	598	0.1%	1.83 [0.88, 3.78]
WACS 2007Low	502	4083	124	1022	2.0%	1.01 [0.84, 1.22]
WAVE 2002Low	16	212	6	211	0.1%	2.65 [1.06, 6.65]
White 2002Low	1	50	1	50	0.0%	1.00 [0.06, 15.55]
WHS 2005Low	681	19937	681	19939	6.3%	1.00 [0.90, 1.11]
Witte 2005Low	1	16	1	16	0.0%	1.00 [0.07, 14.64]
Wluka 2002Low	1	67	0	69	0.0%	3.09 [0.13, 74.50]
**Subtotal (95% CI)**		**97523**		**81003**	**88.3%**	**1.01 [0.98, 1.03]**
Total events	9249		7975			
Heterogeneity: Tau² = 0.00; Chi² = 36.98, df = 45 (P = 0.80); I² = 0%						
Test for overall effect: Z = 0.39 (P = 0.70)						
**2.2.2 High Bias Risk**						
H ADCS 1 1997	12	85	12	84	0.1%	0.99 [0.47, 2.07]
H ADCS 2 2005	5	257	5	259	0.0%	1.01 [0.30, 3.44]
H Bonelli 1998	1	147	0	157	0.0%	3.20 [0.13, 78.00]
H Chandra 1992	0	48	2	48	0.0%	0.20 [0.01, 4.06]
H CTNS 2008	77	510	81	510	0.8%	0.95 [0.71, 1.27]
H de la Maza 1995	5	37	4	37	0.0%	1.25 [0.36, 4.29]
H de Waart 2001	0	109	1	109	0.0%	0.33 [0.01, 8.09]
H Gillilan 1977	2	26	2	26	0.0%	1.00 [0.15, 6.57]
H Girodon 1997	12	41	7	20	0.1%	0.84 [0.39, 1.79]
H GISSI 1999	488	5660	529	5664	5.0%	0.92 [0.82, 1.04]
H McKeown-Eys 1988	4	96	3	89	0.0%	1.24 [0.28, 5.37]
H NIT1 1993	1018	14792	280	3698	4.2%	0.91 [0.80, 1.03]
H Penn 1991	1	15	0	15	0.0%	3.00 [0.13, 68.26]
H PPP 2001	72	2231	68	2264	0.6%	1.07 [0.78, 1.49]
H SIT 2001	38	1706	43	1705	0.4%	0.88 [0.57, 1.36]
H Stevic 2001	3	16	6	12	0.1%	0.38 [0.12, 1.20]
H Takagi 2003	10	51	16	42	0.1%	0.51 [0.26, 1.01]
H Takmatsu 1995	1	74	0	73	0.0%	2.96 [0.12, 71.50]
**Subtotal (95% CI)**		**25901**		**14812**	**11.7%**	**0.92 [0.85, 0.99]**
Total events	1749		1059			
Heterogeneity: Tau² = 0.00; Chi² = 9.65, df = 17 (P = 0.92); I² = 0%						
Test for overall effect: Z = 2.18 (P = 0.03)						
**Total (95% CI)**		**123424**		**95815**	**100.0%**	**0.99 [0.97, 1.02]**
Total events	10998		9034			
Heterogeneity: Tau² = 0.00; Chi² = 51.38, df = 63 (P = 0.85); I² = 0%						
Test for overall effect: Z = 0.38 (P = 0.70)						
Test for subgroup differences: Chi² = 4.76, df = 1 (P = 0.03), I² = 79.0%						

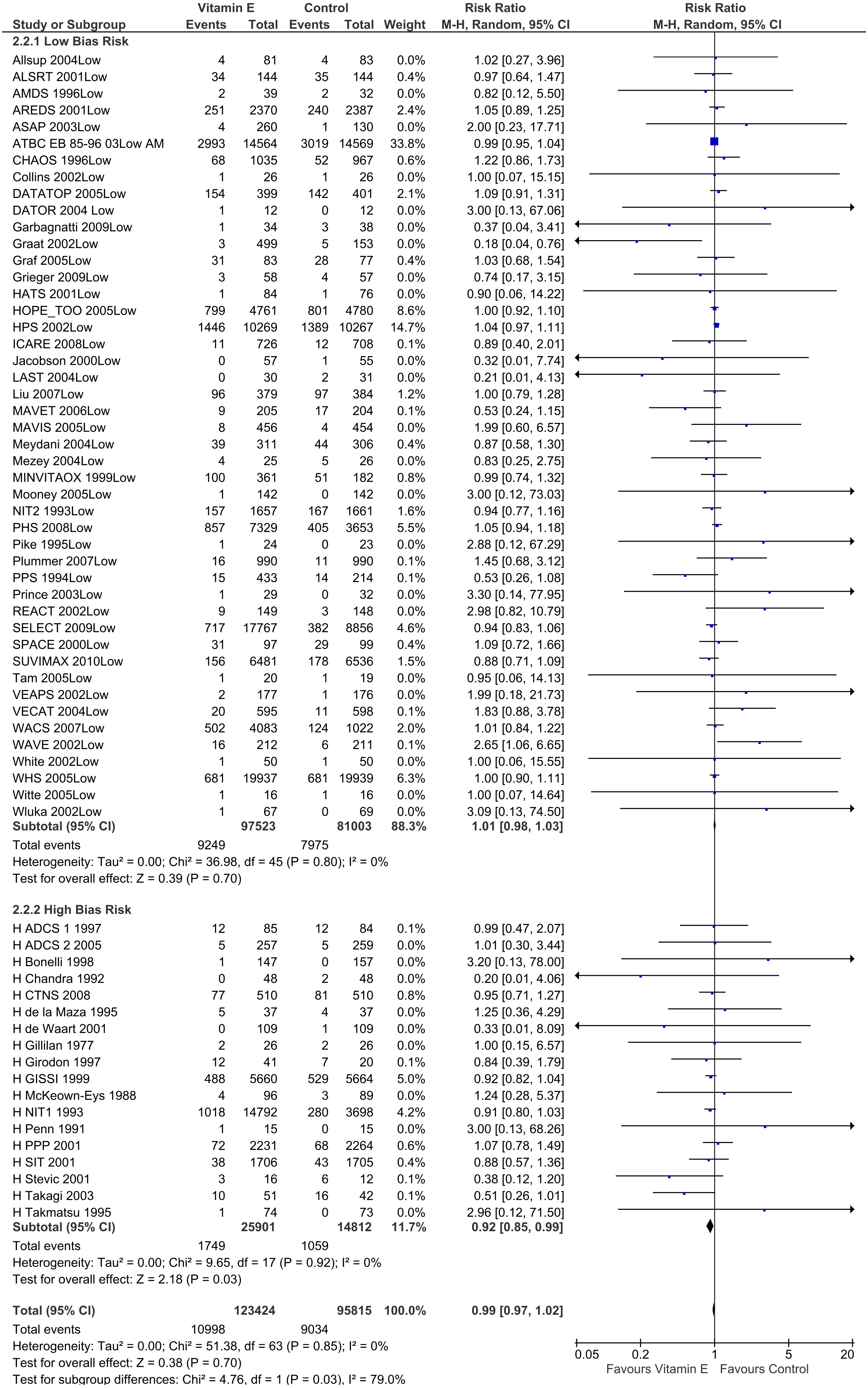

Figure S6 - A11 - ATBC 85-99 "At the Margins" (4 cell)

Study or Subgroup	Vitamin E Events	Vitamin E Total	Control Events	Control Total	Weight	Risk Ratio M-H, Random, 95% CI
**2.3.1 Low Bias Risk**						
Allsup 2004Low	4	81	4	83	0.0%	1.02 [0.27, 3.96]
ALSRT 2001Low	34	144	35	144	0.3%	0.97 [0.64, 1.47]
AMDS 1996Low	2	39	2	32	0.0%	0.82 [0.12, 5.50]
AREDS 2001Low	251	2370	240	2387	2.0%	1.05 [0.89, 1.25]
ASAP 2003Low	4	260	1	130	0.0%	2.00 [0.23, 17.71]
ATBC EB 85-99 03Low AM	4453	14564	4415	14569	46.2%	1.01 [0.97, 1.04]
CHAOS 1996Low	68	1035	52	967	0.5%	1.22 [0.86, 1.73]
Collins 2002Low	1	26	1	26	0.0%	1.00 [0.07, 15.15]
DATATOP 2005Low	154	399	142	401	1.7%	1.09 [0.91, 1.31]
DATOR 2004 Low	1	12	0	12	0.0%	3.00 [0.13, 67.06]
Garbagnatti 2009Low	1	34	3	38	0.0%	0.37 [0.04, 3.41]
Graat 2002Low	3	499	5	153	0.0%	0.18 [0.04, 0.76]
Graf 2005Low	31	83	28	77	0.3%	1.03 [0.68, 1.54]
Grieger 2009Low	3	58	4	57	0.0%	0.74 [0.17, 3.15]
HATS 2001Low	1	84	1	76	0.0%	0.90 [0.06, 14.22]
HOPE_TOO 2005Low	799	4761	801	4780	7.0%	1.00 [0.92, 1.10]
HPS 2002Low	1446	10269	1389	10267	11.9%	1.04 [0.97, 1.11]
ICARE 2008Low	11	726	12	708	0.1%	0.89 [0.40, 2.01]
Jacobson 2000Low	0	57	1	55	0.0%	0.32 [0.01, 7.74]
LAST 2004Low	0	30	2	31	0.0%	0.21 [0.01, 4.13]
Liu 2007Low	96	379	97	384	0.9%	1.00 [0.79, 1.28]
MAVET 2006Low	9	205	17	204	0.1%	0.53 [0.24, 1.15]
MAVIS 2005Low	8	456	4	454	0.0%	1.99 [0.60, 6.57]
Meydani 2004Low	39	311	44	306	0.3%	0.87 [0.58, 1.30]
Mezey 2004Low	4	25	5	26	0.0%	0.83 [0.25, 2.75]
MINVITAOX 1999Low	100	361	51	182	0.7%	0.99 [0.74, 1.32]
Mooney 2005Low	1	142	0	142	0.0%	3.00 [0.12, 73.03]
NIT2 1993Low	157	1657	167	1661	1.3%	0.94 [0.77, 1.16]
PHS 2008Low	857	7329	405	3653	4.5%	1.05 [0.94, 1.18]
Pike 1995Low	1	24	0	23	0.0%	2.88 [0.12, 67.29]
Plummer 2007Low	16	990	11	990	0.1%	1.45 [0.68, 3.12]
PPS 1994Low	15	433	14	214	0.1%	0.53 [0.26, 1.08]
Prince 2003Low	1	29	0	32	0.0%	3.30 [0.14, 77.95]
REACT 2002Low	9	149	3	148	0.0%	2.98 [0.82, 10.79]
SELECT 2009Low	717	17767	382	8856	3.8%	0.94 [0.83, 1.06]
SPACE 2000Low	31	97	29	99	0.3%	1.09 [0.72, 1.66]
SUVIMAX 2010Low	156	6481	178	6536	1.2%	0.88 [0.71, 1.09]
Tam 2005Low	1	20	1	19	0.0%	0.95 [0.06, 14.13]
VEAPS 2002Low	2	177	1	176	0.0%	1.99 [0.18, 21.73]
VECAT 2004Low	20	595	11	598	0.1%	1.83 [0.88, 3.78]
WACS 2007Low	502	4083	124	1022	1.6%	1.01 [0.84, 1.22]
WAVE 2002Low	16	212	6	211	0.1%	2.65 [1.06, 6.65]
White 2002Low	1	50	1	50	0.0%	1.00 [0.06, 15.55]
WHS 2005Low	681	19937	681	19939	5.1%	1.00 [0.90, 1.11]
Witte 2005Low	1	16	1	16	0.0%	1.00 [0.07, 14.64]
Wluka 2002Low	1	67	0	69	0.0%	3.09 [0.13, 74.50]
**Subtotal (95% CI)**		**97523**		**81003**	**90.5%**	**1.01 [0.99, 1.04]**
Total events	10709		9371			
Heterogeneity: Tau² = 0.00; Chi² = 36.43, df = 45 (P = 0.81); I² = 0%						
Test for overall effect: Z = 0.90 (P = 0.37)						
**2.3.2 High Bias Risk**						
H ADCS 1 1997	12	85	12	84	0.1%	0.99 [0.47, 2.07]
H ADCS 2 2005	5	257	5	259	0.0%	1.01 [0.30, 3.44]
H Bonelli 1998	1	147	0	157	0.0%	3.20 [0.13, 78.00]
H Chandra 1992	0	48	2	48	0.0%	0.20 [0.01, 4.06]
H CTNS 2008	77	510	81	510	0.7%	0.95 [0.71, 1.27]
H de la Maza 1995	5	37	4	37	0.0%	1.25 [0.36, 4.29]
H de Waart 2001	0	109	1	109	0.0%	0.33 [0.01, 8.09]
H Gillilan 1977	2	26	2	26	0.0%	1.00 [0.15, 6.57]
H Girodon 1997	12	41	7	20	0.1%	0.84 [0.39, 1.79]
H GISSI 1999	488	5660	529	5664	4.0%	0.92 [0.82, 1.04]
H McKeown-Eys 1988	4	96	3	89	0.0%	1.24 [0.28, 5.37]
H NIT1 1993	1018	14792	280	3698	3.4%	0.91 [0.80, 1.03]
H Penn 1991	1	15	0	15	0.0%	3.00 [0.13, 68.26]
H PPP 2001	72	2231	68	2264	0.5%	1.07 [0.78, 1.49]
H SIT 2001	38	1706	43	1705	0.3%	0.88 [0.57, 1.36]
H Stevic 2001	3	16	6	12	0.0%	0.38 [0.12, 1.20]
H Takagi 2003	10	51	16	42	0.1%	0.51 [0.26, 1.01]
H Takmatsu 1995	1	74	0	73	0.0%	2.96 [0.12, 71.50]
**Subtotal (95% CI)**		**25901**		**14812**	**9.5%**	**0.92 [0.85, 0.99]**
Total events	1749		1059			
Heterogeneity: Tau² = 0.00; Chi² = 9.65, df = 17 (P = 0.92); I² = 0%						
Test for overall effect: Z = 2.18 (P = 0.03)						
**Total (95% CI)**		**123424**		**95815**	**100.0%**	**1.00 [0.98, 1.03]**
Total events	12458		10430			
Heterogeneity: Tau² = 0.00; Chi² = 51.62, df = 63 (P = 0.85); I² = 0%						
Test for overall effect: Z = 0.19 (P = 0.85)						
Test for subgroup differences: Chi² = 5.53, df = 1 (P = 0.02), I² = 81.9%						

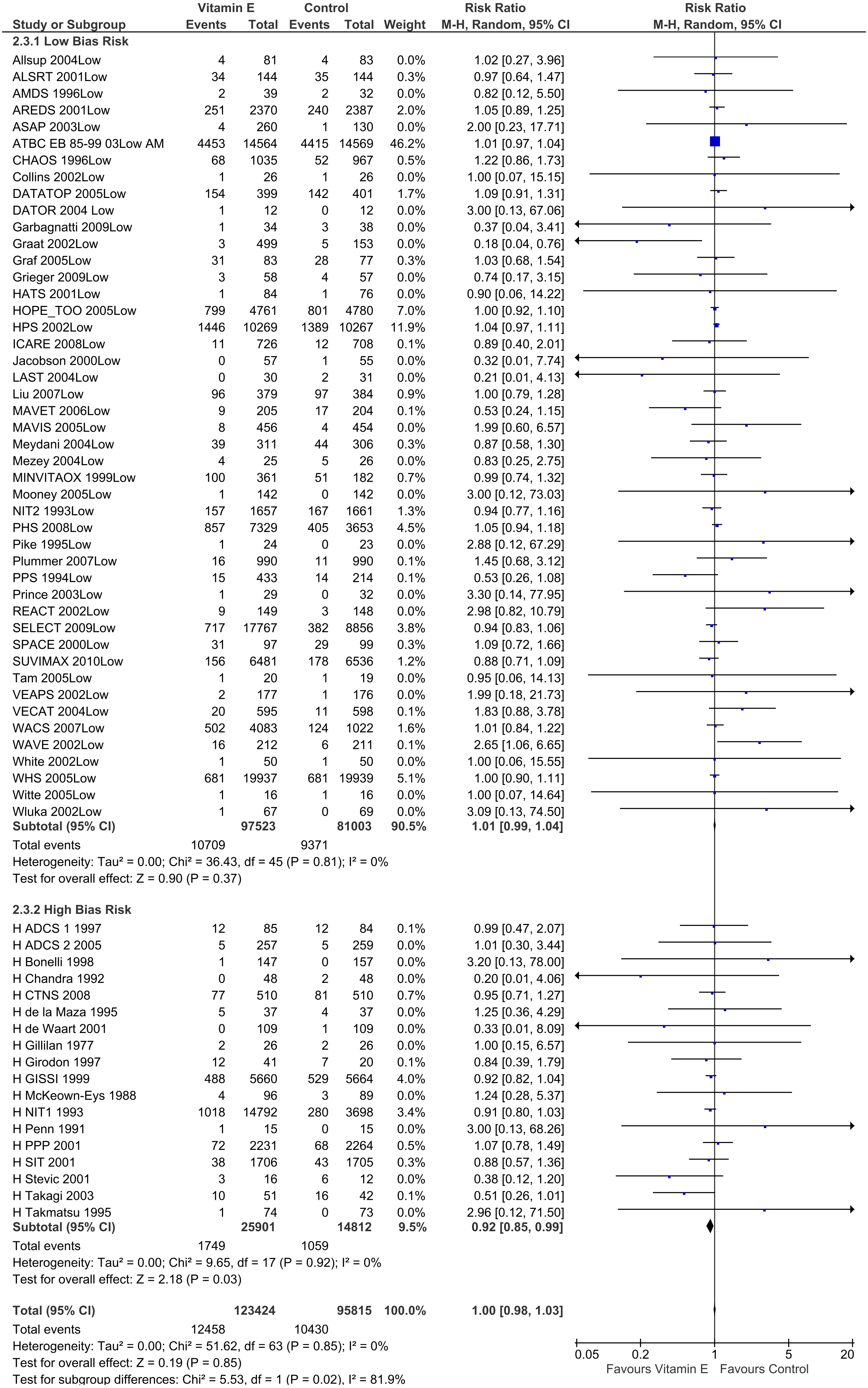

Figure S7 - A11 - ATBC 85-01 "At the Margins" (4 cell)

Study or Subgroup	Vitamin E Events	Vitamin E Total	Control Events	Control Total	Weight	Risk Ratio M-H, Random, 95% CI
**2.4.1 Low Bias Risk**						
Allsup 2004Low	4	81	4	83	0.0%	1.02 [0.27, 3.96]
ALSRT 2001Low	34	144	35	144	0.3%	0.97 [0.64, 1.47]
AMDS 1996Low	2	39	2	32	0.0%	0.82 [0.12, 5.50]
AREDS 2001Low	251	2370	240	2387	1.7%	1.05 [0.89, 1.25]
ASAP 2003Low	4	260	1	130	0.0%	2.00 [0.23, 17.71]
ATBC EB 85-01 03Low AM	5433	14564	5398	14569	53.7%	1.01 [0.98, 1.04]
CHAOS 1996Low	68	1035	52	967	0.4%	1.22 [0.86, 1.73]
Collins 2002Low	1	26	1	26	0.0%	1.00 [0.07, 15.15]
DATATOP 2005Low	154	399	142	401	1.5%	1.09 [0.91, 1.31]
DATOR 2004 Low	1	12	0	12	0.0%	3.00 [0.13, 67.06]
Garbagnatti 2009Low	1	34	3	38	0.0%	0.37 [0.04, 3.41]
Graat 2002Low	3	499	5	153	0.0%	0.18 [0.04, 0.76]
Graf 2005Low	31	83	28	77	0.3%	1.03 [0.68, 1.54]
Grieger 2009Low	3	58	4	57	0.0%	0.74 [0.17, 3.15]
HATS 2001Low	1	84	1	76	0.0%	0.90 [0.06, 14.22]
HOPE_TOO 2005Low	799	4761	801	4780	6.0%	1.00 [0.92, 1.10]
HPS 2002Low	1446	10269	1389	10267	10.2%	1.04 [0.97, 1.11]
ICARE 2008Low	11	726	12	708	0.1%	0.89 [0.40, 2.01]
Jacobson 2000Low	0	57	1	55	0.0%	0.32 [0.01, 7.74]
LAST 2004Low	0	30	2	31	0.0%	0.21 [0.01, 4.13]
Liu 2007Low	96	379	97	384	0.8%	1.00 [0.79, 1.28]
MAVET 2006Low	9	205	17	204	0.1%	0.53 [0.24, 1.15]
MAVIS 2005Low	8	456	4	454	0.0%	1.99 [0.60, 6.57]
Meydani 2004Low	39	311	44	306	0.3%	0.87 [0.58, 1.30]
Mezey 2004Low	4	25	5	26	0.0%	0.83 [0.25, 2.75]
MINVITAOX 1999Low	100	361	51	182	0.6%	0.99 [0.74, 1.32]
Mooney 2005Low	1	142	0	142	0.0%	3.00 [0.12, 73.03]
NIT2 1993Low	157	1657	167	1661	1.1%	0.94 [0.77, 1.16]
PHS 2008Low	857	7329	405	3653	3.9%	1.05 [0.94, 1.18]
Pike 1995Low	1	24	0	23	0.0%	2.88 [0.12, 67.29]
Plummer 2007Low	16	990	11	990	0.1%	1.45 [0.68, 3.12]
PPS 1994Low	15	433	14	214	0.1%	0.53 [0.26, 1.08]
Prince 2003Low	1	29	0	32	0.0%	3.30 [0.14, 77.95]
REACT 2002Low	9	149	3	148	0.0%	2.98 [0.82, 10.79]
SELECT 2009Low	717	17767	382	8856	3.2%	0.94 [0.83, 1.06]
SPACE 2000Low	31	97	29	99	0.3%	1.09 [0.72, 1.66]
SUVIMAX 2010Low	156	6481	178	6536	1.1%	0.88 [0.71, 1.09]
Tam 2005Low	1	20	1	19	0.0%	0.95 [0.06, 14.13]
VEAPS 2002Low	2	177	1	176	0.0%	1.99 [0.18, 21.73]
VECAT 2004Low	20	595	11	598	0.1%	1.83 [0.88, 3.78]
WACS 2007Low	502	4083	124	1022	1.4%	1.01 [0.84, 1.22]
WAVE 2002Low	16	212	6	211	0.1%	2.65 [1.06, 6.65]
White 2002Low	1	50	1	50	0.0%	1.00 [0.06, 15.55]
WHS 2005Low	681	19937	681	19939	4.4%	1.00 [0.90, 1.11]
Witte 2005Low	1	16	1	16	0.0%	1.00 [0.07, 14.64]
Wluka 2002Low	1	67	0	69	0.0%	3.09 [0.13, 74.50]
**Subtotal (95% CI)**		**97523**		**81003**	**91.8%**	**1.01 [0.99, 1.03]**
Total events	11689		10354			
Heterogeneity: Tau² = 0.00; Chi² = 36.49, df = 45 (P = 0.81); I² = 0%						
Test for overall effect: Z = 0.84 (P = 0.40)						
**2.4.2 High Bias Risk**						
H ADCS 1 1997	12	85	12	84	0.1%	0.99 [0.47, 2.07]
H ADCS 2 2005	5	257	5	259	0.0%	1.01 [0.30, 3.44]
H Bonelli 1998	1	147	0	157	0.0%	3.20 [0.13, 78.00]
H Chandra 1992	0	48	2	48	0.0%	0.20 [0.01, 4.06]
H CTNS 2008	77	510	81	510	0.6%	0.95 [0.71, 1.27]
H de la Maza 1995	5	37	4	37	0.0%	1.25 [0.36, 4.29]
H de Waart 2001	0	109	1	109	0.0%	0.33 [0.01, 8.09]
H Gillilan 1977	2	26	2	26	0.0%	1.00 [0.15, 6.57]
H Girodon 1997	12	41	7	20	0.1%	0.84 [0.39, 1.79]
H GISSI 1999	488	5660	529	5664	3.5%	0.92 [0.82, 1.04]
H McKeown-Eys 1988	4	96	3	89	0.0%	1.24 [0.28, 5.37]
H NIT1 1993	1018	14792	280	3698	3.0%	0.91 [0.80, 1.03]
H Penn 1991	1	15	0	15	0.0%	3.00 [0.13, 68.26]
H PPP 2001	72	2231	68	2264	0.4%	1.07 [0.78, 1.49]
H SIT 2001	38	1706	43	1705	0.3%	0.88 [0.57, 1.36]
H Stevic 2001	3	16	6	12	0.0%	0.38 [0.12, 1.20]
H Takagi 2003	10	51	16	42	0.1%	0.51 [0.26, 1.01]
H Takmatsu 1995	1	74	0	73	0.0%	2.96 [0.12, 71.50]
**Subtotal (95% CI)**		**25901**		**14812**	**8.2%**	**0.92 [0.85, 0.99]**
Total events	1749		1059			
Heterogeneity: Tau² = 0.00; Chi² = 9.65, df = 17 (P = 0.92); I² = 0%						
Test for overall effect: Z = 2.18 (P = 0.03)						
**Total (95% CI)**		**123424**		**95815**	**100.0%**	**1.00 [0.98, 1.02]**
Total events	13438		11413			
Heterogeneity: Tau² = 0.00; Chi² = 51.57, df = 63 (P = 0.85); I² = 0%						
Test for overall effect: Z = 0.19 (P = 0.85)						
Test for subgroup differences: Chi² = 5.43, df = 1 (P = 0.02), I² = 81.6%						

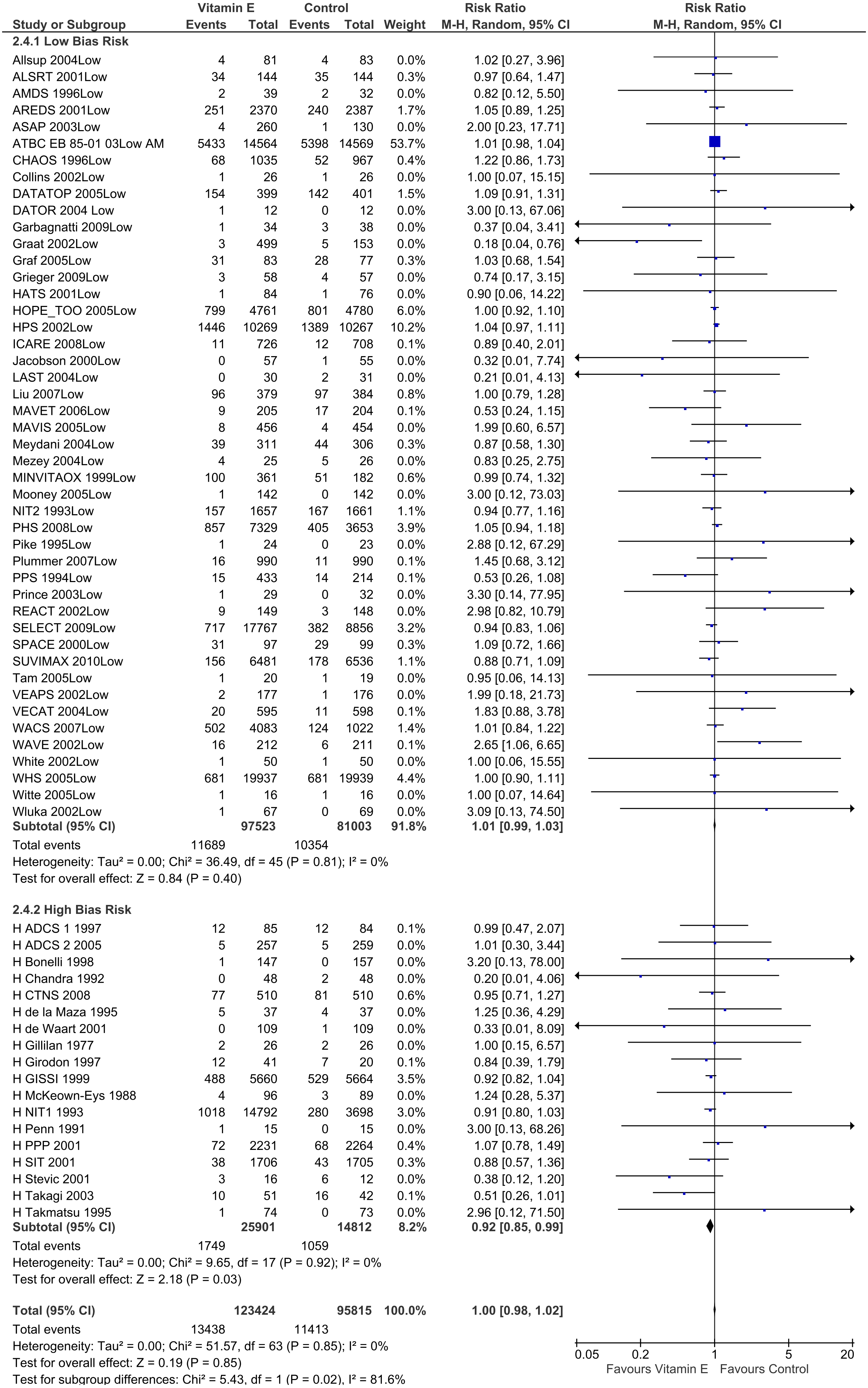

Figure S8 - A11 - ATBC 85-11 "At the Margins" (4 cell)

Study or Subgroup	Vitamin E Events	Vitamin E Total	Control Events	Control Total	Weight	Risk Ratio M-H, Random, 95% CI
**2.6.1 Low Bias Risk**						
Allsup 2004Low	4	81	4	83	0.0%	1.02 [0.27, 3.96]
ALSRT 2001Low	34	144	35	144	0.1%	0.97 [0.64, 1.47]
AMDS 1996Low	2	39	2	32	0.0%	0.82 [0.12, 5.50]
AREDS 2001Low	251	2370	240	2387	0.7%	1.05 [0.89, 1.25]
ASAP 2003Low	4	260	1	130	0.0%	2.00 [0.23, 17.71]
ATBC EB 85-11 AM	10182	14564	10074	14569	81.7%	1.01 [1.00, 1.03]
CHAOS 1996Low	68	1035	52	967	0.2%	1.22 [0.86, 1.73]
Collins 2002Low	1	26	1	26	0.0%	1.00 [0.07, 15.15]
DATATOP 2005Low	154	399	142	401	0.6%	1.09 [0.91, 1.31]
DATOR 2004 Low	1	12	0	12	0.0%	3.00 [0.13, 67.06]
Garbagnatti 2009Low	1	34	3	38	0.0%	0.37 [0.04, 3.41]
Graat 2002Low	3	499	5	153	0.0%	0.18 [0.04, 0.76]
Graf 2005Low	31	83	28	77	0.1%	1.03 [0.68, 1.54]
Grieger 2009Low	3	58	4	57	0.0%	0.74 [0.17, 3.15]
HATS 2001Low	1	84	1	76	0.0%	0.90 [0.06, 14.22]
HOPE_TOO 2005Low	799	4761	801	4780	2.4%	1.00 [0.92, 1.10]
HPS 2002Low	1446	10269	1389	10267	4.0%	1.04 [0.97, 1.11]
ICARE 2008Low	11	726	12	708	0.0%	0.89 [0.40, 2.01]
Jacobson 2000Low	0	57	1	55	0.0%	0.32 [0.01, 7.74]
LAST 2004Low	0	30	2	31	0.0%	0.21 [0.01, 4.13]
Liu 2007Low	96	379	97	384	0.3%	1.00 [0.79, 1.28]
MAVET 2006Low	9	205	17	204	0.0%	0.53 [0.24, 1.15]
MAVIS 2005Low	8	456	4	454	0.0%	1.99 [0.60, 6.57]
Meydani 2004Low	39	311	44	306	0.1%	0.87 [0.58, 1.30]
Mezey 2004Low	4	25	5	26	0.0%	0.83 [0.25, 2.75]
MINVITAOX 1999Low	100	361	51	182	0.2%	0.99 [0.74, 1.32]
Mooney 2005Low	1	142	0	142	0.0%	3.00 [0.12, 73.03]
NIT2 1993Low	157	1657	167	1661	0.4%	0.94 [0.77, 1.16]
PHS 2008Low	857	7329	405	3653	1.5%	1.05 [0.94, 1.18]
Pike 1995Low	1	24	0	23	0.0%	2.88 [0.12, 67.29]
Plummer 2007Low	16	990	11	990	0.0%	1.45 [0.68, 3.12]
PPS 1994Low	15	433	14	214	0.0%	0.53 [0.26, 1.08]
Prince 2003Low	1	29	0	32	0.0%	3.30 [0.14, 77.95]
REACT 2002Low	9	149	3	148	0.0%	2.98 [0.82, 10.79]
SELECT 2009Low	717	17767	382	8856	1.3%	0.94 [0.83, 1.06]
SPACE 2000Low	31	97	29	99	0.1%	1.09 [0.72, 1.66]
SUVIMAX 2010Low	156	6481	178	6536	0.4%	0.88 [0.71, 1.09]
Tam 2005Low	1	20	1	19	0.0%	0.95 [0.06, 14.13]
VEAPS 2002Low	2	177	1	176	0.0%	1.99 [0.18, 21.73]
VECAT 2004Low	20	595	11	598	0.0%	1.83 [0.88, 3.78]
WACS 2007Low	502	4083	124	1022	0.6%	1.01 [0.84, 1.22]
WAVE 2002Low	16	212	6	211	0.0%	2.65 [1.06, 6.65]
White 2002Low	1	50	1	50	0.0%	1.00 [0.06, 15.55]
WHS 2005Low	681	19937	681	19939	1.7%	1.00 [0.90, 1.11]
Witte 2005Low	1	16	1	16	0.0%	1.00 [0.07, 14.64]
Wluka 2002Low	1	67	0	69	0.0%	3.09 [0.13, 74.50]
**Subtotal (95% CI)**		**97523**		**81003**	**96.8%**	**1.01 [1.00, 1.03]**
Total events	16438		15030			
Heterogeneity: Tau² = 0.00; Chi² = 36.42, df = 45 (P = 0.82); I² = 0%						
Test for overall effect: Z = 1.61 (P = 0.11)						
**2.6.2 High Bias Risk**						
H ADCS 1 1997	12	85	12	84	0.0%	0.99 [0.47, 2.07]
H ADCS 2 2005	5	257	5	259	0.0%	1.01 [0.30, 3.44]
H Bonelli 1998	1	147	0	157	0.0%	3.20 [0.13, 78.00]
H Chandra 1992	0	48	2	48	0.0%	0.20 [0.01, 4.06]
H CTNS 2008	77	510	81	510	0.2%	0.95 [0.71, 1.27]
H de la Maza 1995	5	37	4	37	0.0%	1.25 [0.36, 4.29]
H de Waart 2001	0	109	1	109	0.0%	0.33 [0.01, 8.09]
H Gillilan 1977	2	26	2	26	0.0%	1.00 [0.15, 6.57]
H Girodon 1997	12	41	7	20	0.0%	0.84 [0.39, 1.79]
H GISSI 1999	488	5660	529	5664	1.4%	0.92 [0.82, 1.04]
H McKeown-Eys 1988	4	96	3	89	0.0%	1.24 [0.28, 5.37]
H NIT1 1993	1018	14792	280	3698	1.2%	0.91 [0.80, 1.03]
H Penn 1991	1	15	0	15	0.0%	3.00 [0.13, 68.26]
H PPP 2001	72	2231	68	2264	0.2%	1.07 [0.78, 1.49]
H SIT 2001	38	1706	43	1705	0.1%	0.88 [0.57, 1.36]
H Stevic 2001	3	16	6	12	0.0%	0.38 [0.12, 1.20]
H Takagi 2003	10	51	16	42	0.0%	0.51 [0.26, 1.01]
H Takmatsu 1995	1	74	0	73	0.0%	2.96 [0.12, 71.50]
**Subtotal (95% CI)**		**25901**		**14812**	**3.2%**	**0.92 [0.85, 0.99]**
Total events	1749		1059			
Heterogeneity: Tau² = 0.00; Chi² = 9.65, df = 17 (P = 0.92); I² = 0%						
Test for overall effect: Z = 2.18 (P = 0.03)						
**Total (95% CI)**		**123424**		**95815**	**100.0%**	**1.01 [0.99, 1.02]**
Total events	18187		16089			
Heterogeneity: Tau² = 0.00; Chi² = 52.21, df = 63 (P = 0.83); I² = 0%						
Test for overall effect: Z = 1.19 (P = 0.23)						
Test for subgroup differences: Chi² = 5.92, df = 1 (P = 0.01), I² = 83.1%						

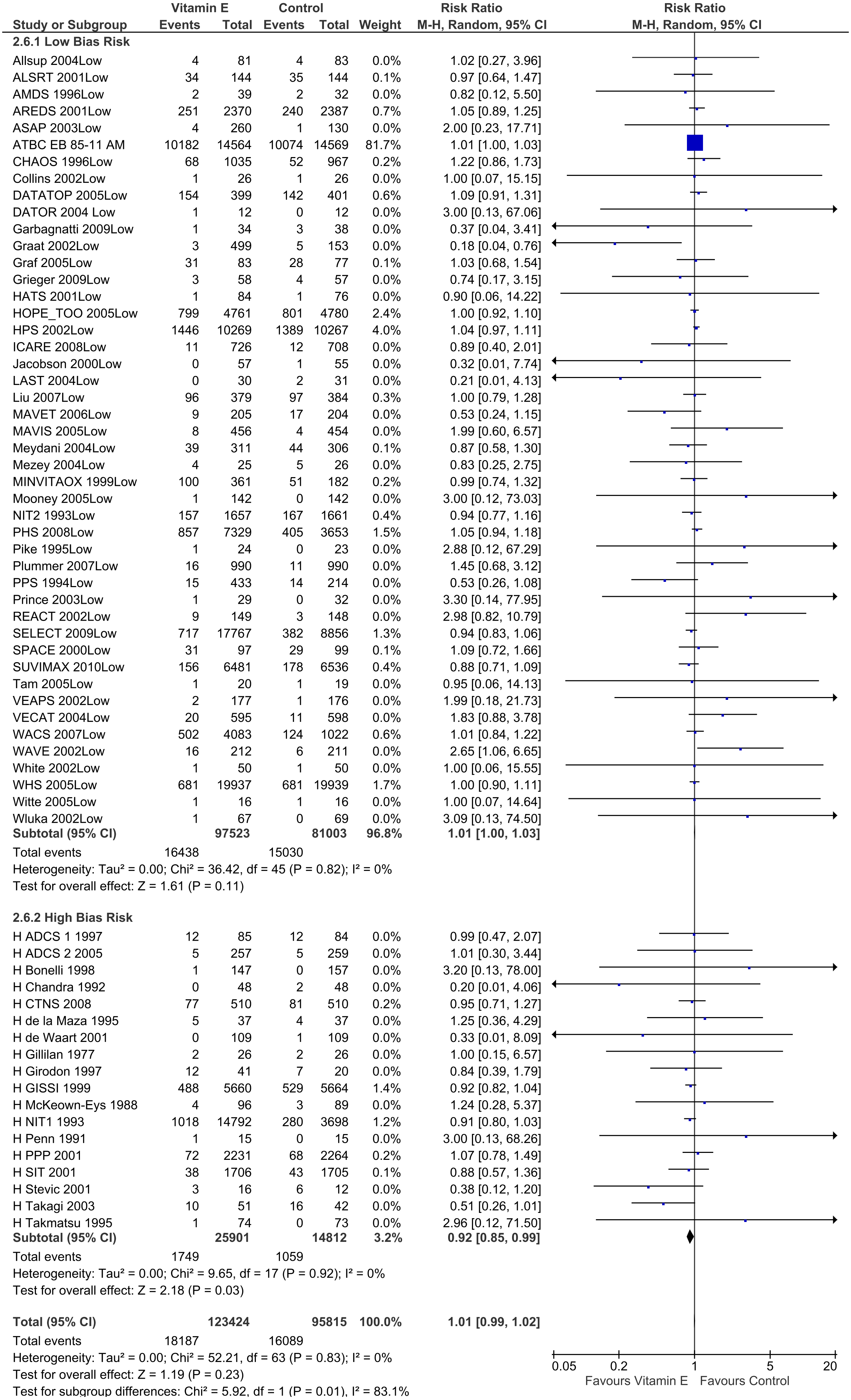

Figure S9 - A11 - ATBC 85-93 "Inside the Table" (2 cell)

Study or Subgroup	Vitamin E Events	Vitamin E Total	Control Events	Control Total	Weight	Risk Ratio M-H, Random, 95% CI
**3.1.1 Low Bias Risk**						
Allsup 2004Low	4	81	4	83	0.1%	1.02 [0.27, 3.96]
ALSRT 2001Low	34	144	35	144	0.5%	0.97 [0.64, 1.47]
AMDS 1996Low	2	39	2	32	0.0%	0.82 [0.12, 5.50]
AREDS 2001Low	251	2370	240	2387	3.3%	1.05 [0.89, 1.25]
ASAP 2003Low	4	260	1	130	0.0%	2.00 [0.23, 17.71]
ATBC E 85-93 03Low	868	7286	851	7287	11.6%	1.02 [0.93, 1.11]
CHAOS 1996Low	68	1035	52	967	0.7%	1.22 [0.86, 1.73]
Collins 2002Low	1	26	1	26	0.0%	1.00 [0.07, 15.15]
DATATOP 2005Low	154	399	142	401	2.8%	1.09 [0.91, 1.31]
DATOR 2004 Low	1	12	0	12	0.0%	3.00 [0.13, 67.06]
Garbagnatti 2009Low	1	34	3	38	0.0%	0.37 [0.04, 3.41]
Graat 2002Low	3	499	5	153	0.0%	0.18 [0.04, 0.76]
Graf 2005Low	31	83	28	77	0.6%	1.03 [0.68, 1.54]
Grieger 2009Low	3	58	4	57	0.0%	0.74 [0.17, 3.15]
HATS 2001Low	1	84	1	76	0.0%	0.90 [0.06, 14.22]
HOPE_TOO 2005Low	799	4761	801	4780	11.4%	1.00 [0.92, 1.10]
HPS 2002Low	1446	10269	1389	10267	19.6%	1.04 [0.97, 1.11]
ICARE 2008Low	11	726	12	708	0.1%	0.89 [0.40, 2.01]
Jacobson 2000Low	0	57	1	55	0.0%	0.32 [0.01, 7.74]
LAST 2004Low	0	30	2	31	0.0%	0.21 [0.01, 4.13]
Liu 2007Low	96	379	97	384	1.5%	1.00 [0.79, 1.28]
MAVET 2006Low	9	205	17	204	0.1%	0.53 [0.24, 1.15]
MAVIS 2005Low	8	456	4	454	0.1%	1.99 [0.60, 6.57]
Meydani 2004Low	39	311	44	306	0.6%	0.87 [0.58, 1.30]
Mezey 2004Low	4	25	5	26	0.1%	0.83 [0.25, 2.75]
MINVITAOX 1999Low	100	361	51	182	1.1%	0.99 [0.74, 1.32]
Mooney 2005Low	1	142	0	142	0.0%	3.00 [0.12, 73.03]
NIT2 1993Low	157	1657	167	1661	2.1%	0.94 [0.77, 1.16]
PHS 2008Low	857	7329	405	3653	7.4%	1.05 [0.94, 1.18]
Pike 1995Low	1	24	0	23	0.0%	2.88 [0.12, 67.29]
Plummer 2007Low	16	990	11	990	0.2%	1.45 [0.68, 3.12]
PPS 1994Low	15	433	14	214	0.2%	0.53 [0.26, 1.08]
Prince 2003Low	1	29	0	32	0.0%	3.30 [0.14, 77.95]
REACT 2002Low	9	149	3	148	0.1%	2.98 [0.82, 10.79]
SELECT 2009Low	717	17767	382	8856	6.2%	0.94 [0.83, 1.06]
SPACE 2000Low	31	97	29	99	0.5%	1.09 [0.72, 1.66]
SUVIMAX 2010Low	156	6481	178	6536	2.0%	0.88 [0.71, 1.09]
Tam 2005Low	1	20	1	19	0.0%	0.95 [0.06, 14.13]
VEAPS 2002Low	2	177	1	176	0.0%	1.99 [0.18, 21.73]
VECAT 2004Low	20	595	11	598	0.2%	1.83 [0.88, 3.78]
WACS 2007Low	502	4083	124	1022	2.7%	1.01 [0.84, 1.22]
WAVE 2002Low	16	212	6	211	0.1%	2.65 [1.06, 6.65]
White 2002Low	1	50	1	50	0.0%	1.00 [0.06, 15.55]
WHS 2005Low	681	19937	681	19939	8.4%	1.00 [0.90, 1.11]
Witte 2005Low	1	16	1	16	0.0%	1.00 [0.07, 14.64]
Wluka 2002Low	1	67	0	69	0.0%	3.09 [0.13, 74.50]
**Subtotal (95% CI)**		**90245**		**73721**	**84.4%**	**1.01 [0.98, 1.05]**
Total events	7124		5807			
Heterogeneity: Tau² = 0.00; Chi² = 36.41, df = 45 (P = 0.82); I² = 0%						
Test for overall effect: Z = 0.89 (P = 0.38)						
**3.1.2 High Bias Risk**						
H ADCS 1 1997	12	85	12	84	0.2%	0.99 [0.47, 2.07]
H ADCS 2 2005	5	257	5	259	0.1%	1.01 [0.30, 3.44]
H Bonelli 1998	1	147	0	157	0.0%	3.20 [0.13, 78.00]
H Chandra 1992	0	48	2	48	0.0%	0.20 [0.01, 4.06]
H CTNS 2008	77	510	81	510	1.1%	0.95 [0.71, 1.27]
H de la Maza 1995	5	37	4	37	0.1%	1.25 [0.36, 4.29]
H de Waart 2001	0	109	1	109	0.0%	0.33 [0.01, 8.09]
H Gillilan 1977	2	26	2	26	0.0%	1.00 [0.15, 6.57]
H Girodon 1997	12	41	7	20	0.2%	0.84 [0.39, 1.79]
H GISSI 1999	488	5660	529	5664	6.6%	0.92 [0.82, 1.04]
H McKeown-Eys 1988	4	96	3	89	0.0%	1.24 [0.28, 5.37]
H NIT1 1993	1018	14792	280	3698	5.6%	0.91 [0.80, 1.03]
H Penn 1991	1	15	0	15	0.0%	3.00 [0.13, 68.26]
H PPP 2001	72	2231	68	2264	0.9%	1.07 [0.78, 1.49]
H SIT 2001	38	1706	43	1705	0.5%	0.88 [0.57, 1.36]
H Stevic 2001	3	16	6	12	0.1%	0.38 [0.12, 1.20]
H Takagi 2003	10	51	16	42	0.2%	0.51 [0.26, 1.01]
H Takmatsu 1995	1	74	0	73	0.0%	2.96 [0.12, 71.50]
**Subtotal (95% CI)**		**25901**		**14812**	**15.6%**	**0.92 [0.85, 0.99]**
Total events	1749		1059			
Heterogeneity: Tau² = 0.00; Chi² = 9.65, df = 17 (P = 0.92); I² = 0%						
Test for overall effect: Z = 2.18 (P = 0.03)						
**Total (95% CI)**		**116146**		**88533**	**100.0%**	**1.00 [0.97, 1.03]**
Total events	8873		6866			
Heterogeneity: Tau² = 0.00; Chi² = 51.59, df = 63 (P = 0.85); I² = 0%						
Test for overall effect: Z = 0.05 (P = 0.96)						
Test for subgroup differences: Chi² = 5.53, df = 1 (P = 0.02), I² = 81.9%						

Risk Ratio
M-H, Random, 95% CI

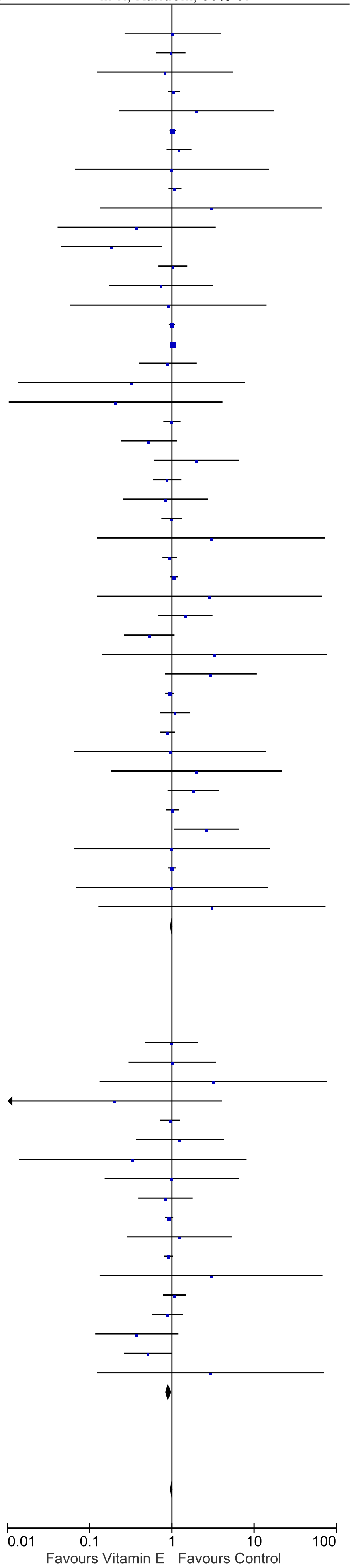

Figure S10 - A11 - ATBC 85-96 "Inside the Table" (2 cell)

Study or Subgroup	Vitamin E Events	Vitamin E Total	Control Events	Control Total	Weight	Risk Ratio M-H, Random, 95% CI
**3.2.1 Low Bias Risk**						
Allsup 2004Low	4	81	4	83	0.0%	1.02 [0.27, 3.96]
ALSRT 2001Low	34	144	35	144	0.5%	0.97 [0.64, 1.47]
AMDS 1996Low	2	39	2	32	0.0%	0.82 [0.12, 5.50]
AREDS 2001Low	251	2370	240	2387	3.0%	1.05 [0.89, 1.25]
ASAP 2003Low	4	260	1	130	0.0%	2.00 [0.23, 17.71]
ATBC E 85-96 03Low	1434	7286	1449	7287	19.5%	0.99 [0.93, 1.06]
CHAOS 1996Low	68	1035	52	967	0.7%	1.22 [0.86, 1.73]
Collins 2002Low	1	26	1	26	0.0%	1.00 [0.07, 15.15]
DATATOP 2005Low	154	399	142	401	2.5%	1.09 [0.91, 1.31]
DATOR 2004 Low	1	12	0	12	0.0%	3.00 [0.13, 67.06]
Garbagnatti 2009Low	1	34	3	38	0.0%	0.37 [0.04, 3.41]
Graat 2002Low	3	499	5	153	0.0%	0.18 [0.04, 0.76]
Graf 2005Low	31	83	28	77	0.5%	1.03 [0.68, 1.54]
Grieger 2009Low	3	58	4	57	0.0%	0.74 [0.17, 3.15]
HATS 2001Low	1	84	1	76	0.0%	0.90 [0.06, 14.22]
HOPE_TOO 2005Low	799	4761	801	4780	10.4%	1.00 [0.92, 1.10]
HPS 2002Low	1446	10269	1389	10267	17.8%	1.04 [0.97, 1.11]
ICARE 2008Low	11	726	12	708	0.1%	0.89 [0.40, 2.01]
Jacobson 2000Low	0	57	1	55	0.0%	0.32 [0.01, 7.74]
LAST 2004Low	0	30	2	31	0.0%	0.21 [0.01, 4.13]
Liu 2007Low	96	379	97	384	1.4%	1.00 [0.79, 1.28]
MAVET 2006Low	9	205	17	204	0.1%	0.53 [0.24, 1.15]
MAVIS 2005Low	8	456	4	454	0.1%	1.99 [0.60, 6.57]
Meydani 2004Low	39	311	44	306	0.5%	0.87 [0.58, 1.30]
Mezey 2004Low	4	25	5	26	0.1%	0.83 [0.25, 2.75]
MINVITAOX 1999Low	100	361	51	182	1.0%	0.99 [0.74, 1.32]
Mooney 2005Low	1	142	0	142	0.0%	3.00 [0.12, 73.03]
NIT2 1993Low	157	1657	167	1661	1.9%	0.94 [0.77, 1.16]
PHS 2008Low	857	7329	405	3653	6.7%	1.05 [0.94, 1.18]
Pike 1995Low	1	24	0	23	0.0%	2.88 [0.12, 67.29]
Plummer 2007Low	16	990	11	990	0.1%	1.45 [0.68, 3.12]
PPS 1994Low	15	433	14	214	0.2%	0.53 [0.26, 1.08]
Prince 2003Low	1	29	0	32	0.0%	3.30 [0.14, 77.95]
REACT 2002Low	9	149	3	148	0.1%	2.98 [0.82, 10.79]
SELECT 2009Low	717	17767	382	8856	5.6%	0.94 [0.83, 1.06]
SPACE 2000Low	31	97	29	99	0.5%	1.09 [0.72, 1.66]
SUVIMAX 2010Low	156	6481	178	6536	1.8%	0.88 [0.71, 1.09]
Tam 2005Low	1	20	1	19	0.0%	0.95 [0.06, 14.13]
VEAPS 2002Low	2	177	1	176	0.0%	1.99 [0.18, 21.73]
VECAT 2004Low	20	595	11	598	0.2%	1.83 [0.88, 3.78]
WACS 2007Low	502	4083	124	1022	2.5%	1.01 [0.84, 1.22]
WAVE 2002Low	16	212	6	211	0.1%	2.65 [1.06, 6.65]
White 2002Low	1	50	1	50	0.0%	1.00 [0.06, 15.55]
WHS 2005Low	681	19937	681	19939	7.6%	1.00 [0.90, 1.11]
Witte 2005Low	1	16	1	16	0.0%	1.00 [0.07, 14.64]
Wluka 2002Low	1	67	0	69	0.0%	3.09 [0.13, 74.50]
**Subtotal (95% CI)**		**90245**		**73721**	**85.8%**	**1.01 [0.98, 1.04]**
Total events	7690		6405			
Heterogeneity: Tau² = 0.00; Chi² = 36.80, df = 45 (P = 0.80); I² = 0%						
Test for overall effect: Z = 0.54 (P = 0.59)						
**3.2.2 High Bias Risk**						
H ADCS 1 1997	12	85	12	84	0.2%	0.99 [0.47, 2.07]
H ADCS 2 2005	5	257	5	259	0.1%	1.01 [0.30, 3.44]
H Bonelli 1998	1	147	0	157	0.0%	3.20 [0.13, 78.00]
H Chandra 1992	0	48	2	48	0.0%	0.20 [0.01, 4.06]
H CTNS 2008	77	510	81	510	1.0%	0.95 [0.71, 1.27]
H de la Maza 1995	5	37	4	37	0.1%	1.25 [0.36, 4.29]
H de Waart 2001	0	109	1	109	0.0%	0.33 [0.01, 8.09]
H Gillilan 1977	2	26	2	26	0.0%	1.00 [0.15, 6.57]
H Girodon 1997	12	41	7	20	0.1%	0.84 [0.39, 1.79]
H GISSI 1999	488	5660	529	5664	6.0%	0.92 [0.82, 1.04]
H Gissi_EO 1999	4	96	3	89	0.0%	1.24 [0.28, 5.37]
H McKeown-Eys 1988	1018	14792	280	3698	5.1%	0.91 [0.80, 1.03]
H NIT1 1993	1	15	0	15	0.0%	3.00 [0.13, 68.26]
H Penn 1991	72	2231	68	2264	0.8%	1.07 [0.78, 1.49]
H PPP 2001	38	1706	43	1705	0.4%	0.88 [0.57, 1.36]
H SIT 2001	3	16	6	12	0.1%	0.38 [0.12, 1.20]
H Stevic 2001	10	51	16	42	0.2%	0.51 [0.26, 1.01]
H Takagi 2003	1	74	0	73	0.0%	2.96 [0.12, 71.50]
H Takmatsu 1995	0	0	0	0		Not estimable
**Subtotal (95% CI)**		**25901**		**14812**	**14.2%**	**0.92 [0.85, 0.99]**
Total events	1749		1059			
Heterogeneity: Tau² = 0.00; Chi² = 9.65, df = 17 (P = 0.92); I² = 0%						
Test for overall effect: Z = 2.18 (P = 0.03)						
**Total (95% CI)**		**116146**		**88533**	**100.0%**	**1.00 [0.97, 1.02]**
Total events	9439		7464			
Heterogeneity: Tau² = 0.00; Chi² = 51.39, df = 63 (P = 0.85); I² = 0%						
Test for overall effect: Z = 0.32 (P = 0.75)						
Test for subgroup differences: Chi² = 4.94, df = 1 (P = 0.03), I² = 79.7%						

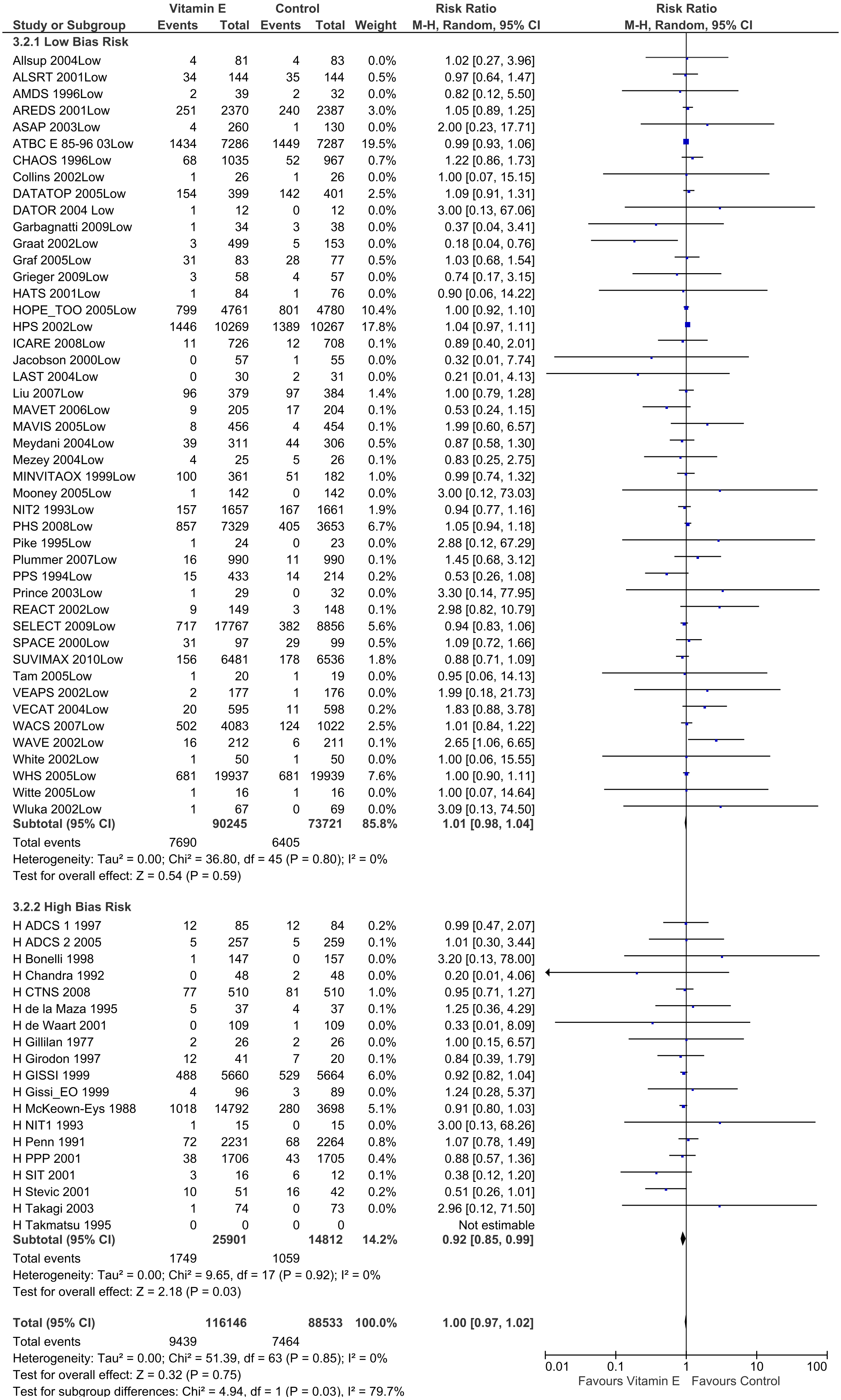

Figure S11 - A11 - ATBC 85-99 "Inside the Table" (2 cell)

Study or Subgroup	Vitamin E Events	Vitamin E Total	Control Events	Control Total	Weight	Risk Ratio M-H, Random, 95% CI
**3.3.1 Low Bias Risk**						
Allsup 2004Low	4	81	4	83	0.0%	1.02 [0.27, 3.96]
ALSRT 2001Low	34	144	35	144	0.4%	0.97 [0.64, 1.47]
AMDS 1996Low	2	39	2	32	0.0%	0.82 [0.12, 5.50]
AREDS 2001Low	251	2370	240	2387	2.6%	1.05 [0.89, 1.25]
ASAP 2003Low	4	260	1	130	0.0%	2.00 [0.23, 17.71]
ATBC E 85-99 03Low	2167	7286	2117	7287	29.0%	1.02 [0.97, 1.08]
CHAOS 1996Low	68	1035	52	967	0.6%	1.22 [0.86, 1.73]
Collins 2002Low	1	26	1	26	0.0%	1.00 [0.07, 15.15]
DATATOP 2005Low	154	399	142	401	2.2%	1.09 [0.91, 1.31]
DATOR 2004 Low	1	12	0	12	0.0%	3.00 [0.13, 67.06]
Garbagnatti 2009Low	1	34	3	38	0.0%	0.37 [0.04, 3.41]
Graat 2002Low	3	499	5	153	0.0%	0.18 [0.04, 0.76]
Graf 2005Low	31	83	28	77	0.4%	1.03 [0.68, 1.54]
Grieger 2009Low	3	58	4	57	0.0%	0.74 [0.17, 3.15]
HATS 2001Low	1	84	1	76	0.0%	0.90 [0.06, 14.22]
HOPE_TOO 2005Low	799	4761	801	4780	9.2%	1.00 [0.92, 1.10]
HPS 2002Low	1446	10269	1389	10267	15.7%	1.04 [0.97, 1.11]
ICARE 2008Low	11	726	12	708	0.1%	0.89 [0.40, 2.01]
Jacobson 2000Low	0	57	1	55	0.0%	0.32 [0.01, 7.74]
LAST 2004Low	0	30	2	31	0.0%	0.21 [0.01, 4.13]
Liu 2007Low	96	379	97	384	1.2%	1.00 [0.79, 1.28]
MAVET 2006Low	9	205	17	204	0.1%	0.53 [0.24, 1.15]
MAVIS 2005Low	8	456	4	454	0.1%	1.99 [0.60, 6.57]
Meydani 2004Low	39	311	44	306	0.5%	0.87 [0.58, 1.30]
Mezey 2004Low	4	25	5	26	0.1%	0.83 [0.25, 2.75]
MINVITAOX 1999Low	100	361	51	182	0.9%	0.99 [0.74, 1.32]
Mooney 2005Low	1	142	0	142	0.0%	3.00 [0.12, 73.03]
NIT2 1993Low	157	1657	167	1661	1.7%	0.94 [0.77, 1.16]
PHS 2008Low	857	7329	405	3653	5.9%	1.05 [0.94, 1.18]
Pike 1995Low	1	24	0	23	0.0%	2.88 [0.12, 67.29]
Plummer 2007Low	16	990	11	990	0.1%	1.45 [0.68, 3.12]
PPS 1994Low	15	433	14	214	0.1%	0.53 [0.26, 1.08]
Prince 2003Low	1	29	0	32	0.0%	3.30 [0.14, 77.95]
REACT 2002Low	9	149	3	148	0.0%	2.98 [0.82, 10.79]
SELECT 2009Low	717	17767	382	8856	5.0%	0.94 [0.83, 1.06]
SPACE 2000Low	31	97	29	99	0.4%	1.09 [0.72, 1.66]
SUVIMAX 2010Low	156	6481	178	6536	1.6%	0.88 [0.71, 1.09]
Tam 2005Low	1	20	1	19	0.0%	0.95 [0.06, 14.13]
VEAPS 2002Low	2	177	1	176	0.0%	1.99 [0.18, 21.73]
VECAT 2004Low	20	595	11	598	0.1%	1.83 [0.88, 3.78]
WACS 2007Low	502	4083	124	1022	2.2%	1.01 [0.84, 1.22]
WAVE 2002Low	16	212	6	211	0.1%	2.65 [1.06, 6.65]
White 2002Low	1	50	1	50	0.0%	1.00 [0.06, 15.55]
WHS 2005Low	681	19937	681	19939	6.7%	1.00 [0.90, 1.11]
Witte 2005Low	1	16	1	16	0.0%	1.00 [0.07, 14.64]
Wluka 2002Low	1	67	0	69	0.0%	3.09 [0.13, 74.50]
**Subtotal (95% CI)**		**90245**		**73721**	**87.5%**	**1.02 [0.99, 1.05]**
Total events	8423		7073			
Heterogeneity: Tau² = 0.00; Chi² = 36.49, df = 45 (P = 0.81); I² = 0%						
Test for overall effect: Z = 1.16 (P = 0.24)						
**3.3.2 High Bias Risk**						
H ADCS 1 1997	12	85	12	84	0.1%	0.99 [0.47, 2.07]
H ADCS 2 2005	5	257	5	259	0.0%	1.01 [0.30, 3.44]
H Bonelli 1998	1	147	0	157	0.0%	3.20 [0.13, 78.00]
H Chandra 1992	0	48	2	48	0.0%	0.20 [0.01, 4.06]
H CTNS 2008	77	510	81	510	0.9%	0.95 [0.71, 1.27]
H de la Maza 1995	5	37	4	37	0.0%	1.25 [0.36, 4.29]
H de Waart 2001	0	109	1	109	0.0%	0.33 [0.01, 8.09]
H Gillilan 1977	2	26	2	26	0.0%	1.00 [0.15, 6.57]
H Girodon 1997	12	41	7	20	0.1%	0.84 [0.39, 1.79]
H GISSI 1999	488	5660	529	5664	5.3%	0.92 [0.82, 1.04]
H McKeown-Eys 1988	4	96	3	89	0.0%	1.24 [0.28, 5.37]
H NIT1 1993	1018	14792	280	3698	4.5%	0.91 [0.80, 1.03]
H Penn 1991	1	15	0	15	0.0%	3.00 [0.13, 68.26]
H PPP 2001	72	2231	68	2264	0.7%	1.07 [0.78, 1.49]
H SIT 2001	38	1706	43	1705	0.4%	0.88 [0.57, 1.36]
H Stevic 2001	3	16	6	12	0.1%	0.38 [0.12, 1.20]
H Takagi 2003	10	51	16	42	0.2%	0.51 [0.26, 1.01]
H Takmatsu 1995	1	74	0	73	0.0%	2.96 [0.12, 71.50]
**Subtotal (95% CI)**		**25901**		**14812**	**12.5%**	**0.92 [0.85, 0.99]**
Total events	1749		1059			
Heterogeneity: Tau² = 0.00; Chi² = 9.65, df = 17 (P = 0.92); I² = 0%						
Test for overall effect: Z = 2.18 (P = 0.03)						
**Total (95% CI)**		**116146**		**88533**	**100.0%**	**1.00 [0.98, 1.03]**
Total events	10172		8132			
Heterogeneity: Tau² = 0.00; Chi² = 52.15, df = 63 (P = 0.83); I² = 0%						
Test for overall effect: Z = 0.32 (P = 0.75)						
Test for subgroup differences: Chi² = 6.00, df = 1 (P = 0.01), I² = 83.3%						

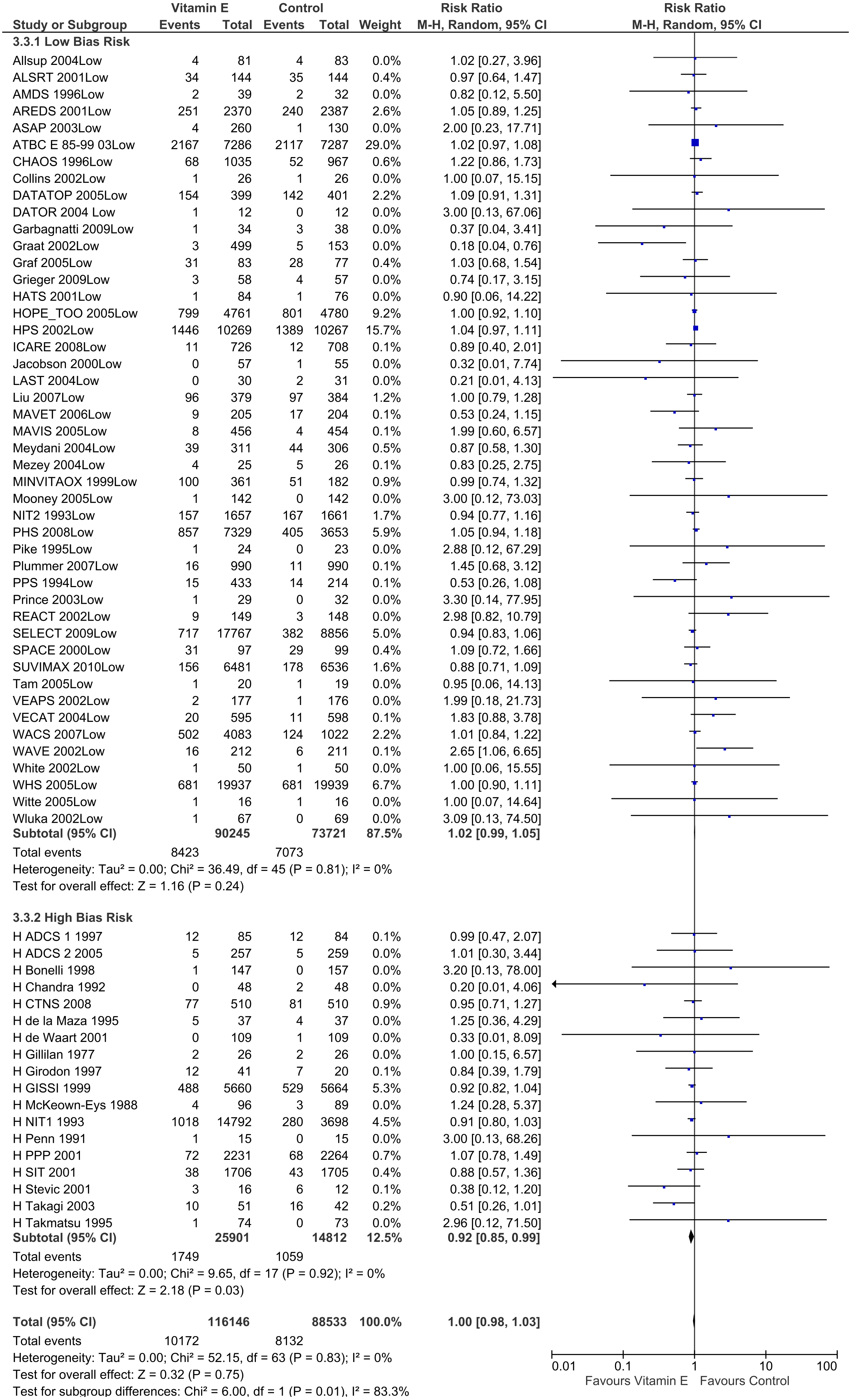

Figure S12 - A11 - ATBC 85-01 "Inside the Table" (2 cell)

	Vitamin E		Control			Risk Ratio
**Study or Subgroup**	**Events**	**Total**	**Events**	**Total**	**Weight**	**M-H, Random, 95% CI**
**3.4.1 Low Bias Risk**						
Allsup 2004Low	4	81	4	83	0.0%	1.02 [0.27, 3.96]
ALSRT 2001Low	34	144	35	144	0.4%	0.97 [0.64, 1.47]
AMDS 1996Low	2	39	2	32	0.0%	0.82 [0.12, 5.50]
AREDS 2001Low	251	2370	240	2387	2.4%	1.05 [0.89, 1.25]
ASAP 2003Low	4	260	1	130	0.0%	2.00 [0.23, 17.71]
ATBC E 85-01 03Low	2671	7286	2605	7287	35.7%	1.03 [0.98, 1.07]
CHAOS 1996Low	68	1035	52	967	0.5%	1.22 [0.86, 1.73]
Collins 2002Low	1	26	1	26	0.0%	1.00 [0.07, 15.15]
DATATOP 2005Low	154	399	142	401	2.0%	1.09 [0.91, 1.31]
DATOR 2004 Low	1	12	0	12	0.0%	3.00 [0.13, 67.06]
Garbagnatti 2009Low	1	34	3	38	0.0%	0.37 [0.04, 3.41]
Graat 2002Low	3	499	5	153	0.0%	0.18 [0.04, 0.76]
Graf 2005Low	31	83	28	77	0.4%	1.03 [0.68, 1.54]
Grieger 2009Low	3	58	4	57	0.0%	0.74 [0.17, 3.15]
HATS 2001Low	1	84	1	76	0.0%	0.90 [0.06, 14.22]
HOPE_TOO 2005Low	799	4761	801	4780	8.3%	1.00 [0.92, 1.10]
HPS 2002Low	1446	10269	1389	10267	14.2%	1.04 [0.97, 1.11]
ICARE 2008Low	11	726	12	708	0.1%	0.89 [0.40, 2.01]
Jacobson 2000Low	0	57	1	55	0.0%	0.32 [0.01, 7.74]
LAST 2004Low	0	30	2	31	0.0%	0.21 [0.01, 4.13]
Liu 2007Low	96	379	97	384	1.1%	1.00 [0.79, 1.28]
MAVET 2006Low	9	205	17	204	0.1%	0.53 [0.24, 1.15]
MAVIS 2005Low	8	456	4	454	0.0%	1.99 [0.60, 6.57]
Meydani 2004Low	39	311	44	306	0.4%	0.87 [0.58, 1.30]
Mezey 2004Low	4	25	5	26	0.0%	0.83 [0.25, 2.75]
MINVITAOX 1999Low	100	361	51	182	0.8%	0.99 [0.74, 1.32]
Mooney 2005Low	1	142	0	142	0.0%	3.00 [0.12, 73.03]
NIT2 1993Low	157	1657	167	1661	1.6%	0.94 [0.77, 1.16]
PHS 2008Low	857	7329	405	3653	5.4%	1.05 [0.94, 1.18]
Pike 1995Low	1	24	0	23	0.0%	2.88 [0.12, 67.29]
Plummer 2007Low	16	990	11	990	0.1%	1.45 [0.68, 3.12]
PPS 1994Low	15	433	14	214	0.1%	0.53 [0.26, 1.08]
Prince 2003Low	1	29	0	32	0.0%	3.30 [0.14, 77.95]
REACT 2002Low	9	149	3	148	0.0%	2.98 [0.82, 10.79]
SELECT 2009Low	717	17767	382	8856	4.5%	0.94 [0.83, 1.06]
SPACE 2000Low	31	97	29	99	0.4%	1.09 [0.72, 1.66]
SUVIMAX 2010Low	156	6481	178	6536	1.5%	0.88 [0.71, 1.09]
Tam 2005Low	1	20	1	19	0.0%	0.95 [0.06, 14.13]
VEAPS 2002Low	2	177	1	176	0.0%	1.99 [0.18, 21.73]
VECAT 2004Low	20	595	11	598	0.1%	1.83 [0.88, 3.78]
WACS 2007Low	502	4083	124	1022	2.0%	1.01 [0.84, 1.22]
WAVE 2002Low	16	212	6	211	0.1%	2.65 [1.06, 6.65]
White 2002Low	1	50	1	50	0.0%	1.00 [0.06, 15.55]
WHS 2005Low	681	19937	681	19939	6.1%	1.00 [0.90, 1.11]
Witte 2005Low	1	16	1	16	0.0%	1.00 [0.07, 14.64]
Wluka 2002Low	1	67	0	69	0.0%	3.09 [0.13, 74.50]
**Subtotal (95% CI)**		**90245**		**73721**	**88.7%**	**1.02 [0.99, 1.05]**
Total events	8927		7561			
Heterogeneity: Tau² = 0.00; Chi² = 36.55, df = 45 (P = 0.81); I² = 0%						
Test for overall effect: Z = 1.33 (P = 0.18)						
**3.4.2 High Bias Risk**						
H ADCS 1 1997	12	85	12	84	0.1%	0.99 [0.47, 2.07]
H ADCS 2 2005	5	257	5	259	0.0%	1.01 [0.30, 3.44]
H Bonelli 1998	1	147	0	157	0.0%	3.20 [0.13, 78.00]
H Chandra 1992	0	48	2	48	0.0%	0.20 [0.01, 4.06]
H CTNS 2008	77	510	81	510	0.8%	0.95 [0.71, 1.27]
H de la Maza 1995	5	37	4	37	0.0%	1.25 [0.36, 4.29]
H de Waart 2001	0	109	1	109	0.0%	0.33 [0.01, 8.09]
H Gillilan 1977	2	26	2	26	0.0%	1.00 [0.15, 6.57]
H Girodon 1997	12	41	7	20	0.1%	0.84 [0.39, 1.79]
H GISSI 1999	488	5660	529	5664	4.8%	0.92 [0.82, 1.04]
H McKeown-Eys 1988	4	96	3	89	0.0%	1.24 [0.28, 5.37]
H NIT1 1993	1018	14792	280	3698	4.1%	0.91 [0.80, 1.03]
H Penn 1991	1	15	0	15	0.0%	3.00 [0.13, 68.26]
H PPP 2001	72	2231	68	2264	0.6%	1.07 [0.78, 1.49]
H SIT 2001	38	1706	43	1705	0.4%	0.88 [0.57, 1.36]
H Stevic 2001	3	16	6	12	0.0%	0.38 [0.12, 1.20]
H Takagi 2003	10	51	16	42	0.1%	0.51 [0.26, 1.01]
H Takmatsu 1995	1	74	0	73	0.0%	2.96 [0.12, 71.50]
**Subtotal (95% CI)**		**25901**		**14812**	**11.3%**	**0.92 [0.85, 0.99]**
Total events	1749		1059			
Heterogeneity: Tau² = 0.00; Chi² = 9.65, df = 17 (P = 0.92); I² = 0%						
Test for overall effect: Z = 2.18 (P = 0.03)						
**Total (95% CI)**		**116146**		**88533**	**100.0%**	**1.01 [0.98, 1.03]**
Total events	10676		8620			
Heterogeneity: Tau² = 0.00; Chi² = 52.47, df = 63 (P = 0.83); I² = 0%						
Test for overall effect: Z = 0.52 (P = 0.60)						
Test for subgroup differences: Chi² = 6.25, df = 1 (P = 0.01), I² = 84.0%						

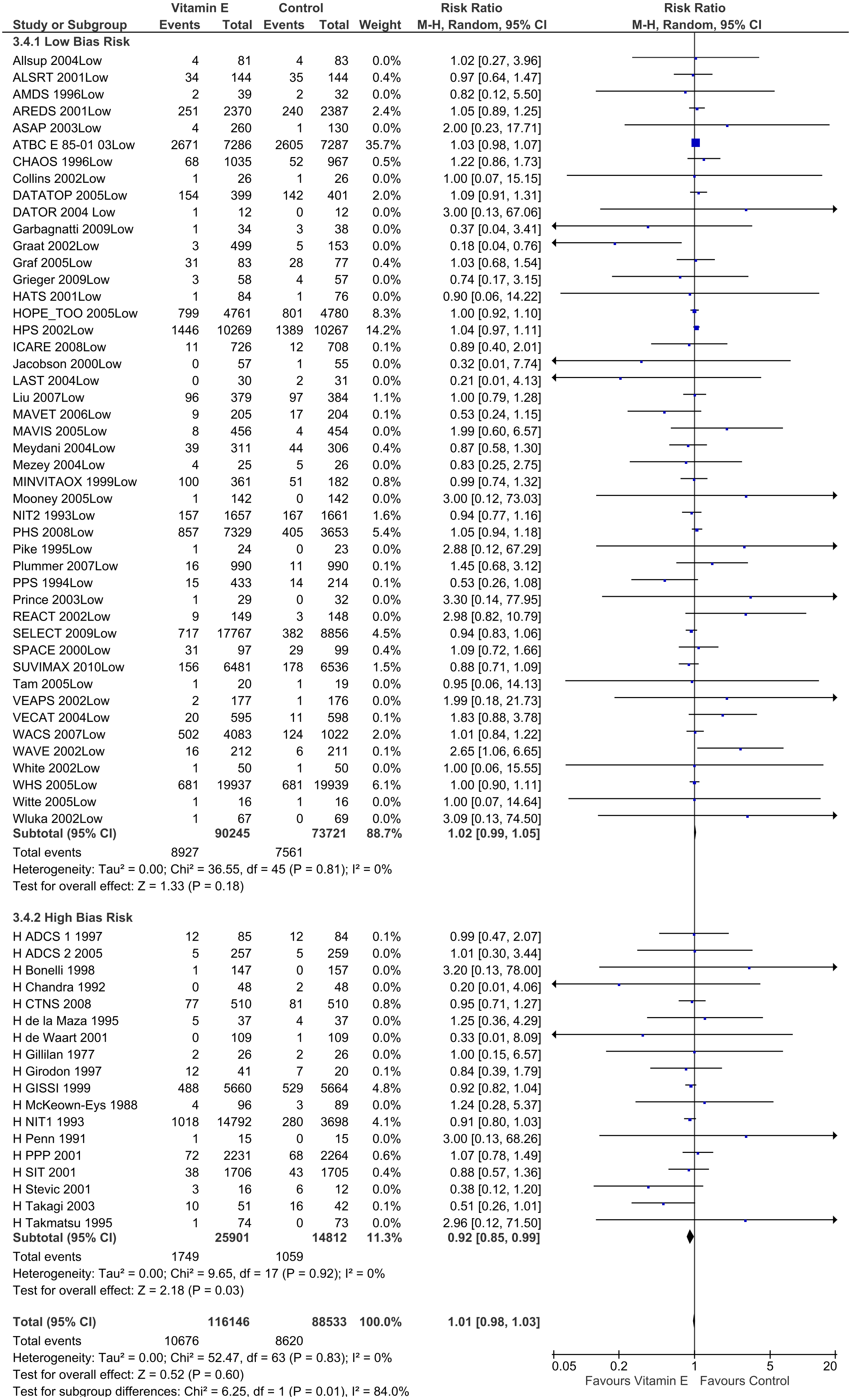

Figure S13 - A11 - ATBC 85-11 "Inside the Table" (2 cell)

Study or Subgroup	Vitamin E Events	Vitamin E Total	Control Events	Control Total	Weight	Risk Ratio M-H, Random, 95% CI
**3.12.1 Low Bias Risk**						
Allsup 2004Low	4	81	4	83	0.0%	1.02 [0.27, 3.96]
ALSRT 2001Low	34	144	35	144	0.2%	0.97 [0.64, 1.47]
AMDS 1996Low	2	39	2	32	0.0%	0.82 [0.12, 5.50]
AREDS 2001Low	251	2370	240	2387	1.1%	1.05 [0.89, 1.25]
ASAP 2003Low	4	260	1	130	0.0%	2.00 [0.23, 17.71]
ATBC E 85-11 IT	5065	7286	5022	7287	68.8%	1.01 [0.99, 1.03]
CHAOS 1996Low	68	1035	52	967	0.3%	1.22 [0.86, 1.73]
Collins 2002Low	1	26	1	26	0.0%	1.00 [0.07, 15.15]
DATATOP 2005Low	154	399	142	401	1.0%	1.09 [0.91, 1.31]
DATOR 2004 Low	1	12	0	12	0.0%	3.00 [0.13, 67.06]
Garbagnatti 2009Low	1	34	3	38	0.0%	0.37 [0.04, 3.41]
Graat 2002Low	3	499	5	153	0.0%	0.18 [0.04, 0.76]
Graf 2005Low	31	83	28	77	0.2%	1.03 [0.68, 1.54]
Grieger 2009Low	3	58	4	57	0.0%	0.74 [0.17, 3.15]
HATS 2001Low	1	84	1	76	0.0%	0.90 [0.06, 14.22]
HOPE_TOO 2005Low	799	4761	801	4780	4.0%	1.00 [0.92, 1.10]
HPS 2002Low	1446	10269	1389	10267	6.9%	1.04 [0.97, 1.11]
ICARE 2008Low	11	726	12	708	0.0%	0.89 [0.40, 2.01]
Jacobson 2000Low	0	57	1	55	0.0%	0.32 [0.01, 7.74]
LAST 2004Low	0	30	2	31	0.0%	0.21 [0.01, 4.13]
Liu 2007Low	96	379	97	384	0.5%	1.00 [0.79, 1.28]
MAVET 2006Low	9	205	17	204	0.1%	0.53 [0.24, 1.15]
MAVIS 2005Low	8	456	4	454	0.0%	1.99 [0.60, 6.57]
Meydani 2004Low	39	311	44	306	0.2%	0.87 [0.58, 1.30]
Mezey 2004Low	4	25	5	26	0.0%	0.83 [0.25, 2.75]
MINVITAOX 1999Low	100	361	51	182	0.4%	0.99 [0.74, 1.32]
Mooney 2005Low	1	142	0	142	0.0%	3.00 [0.12, 73.03]
NIT2 1993Low	157	1657	167	1661	0.8%	0.94 [0.77, 1.16]
PHS 2008Low	857	7329	405	3653	2.6%	1.05 [0.94, 1.18]
Pike 1995Low	1	24	0	23	0.0%	2.88 [0.12, 67.29]
Plummer 2007Low	16	990	11	990	0.1%	1.45 [0.68, 3.12]
PPS 1994Low	15	433	14	214	0.1%	0.53 [0.26, 1.08]
Prince 2003Low	1	29	0	32	0.0%	3.30 [0.14, 77.95]
REACT 2002Low	9	149	3	148	0.0%	2.98 [0.82, 10.79]
SELECT 2009Low	717	17767	382	8856	2.2%	0.94 [0.83, 1.06]
SPACE 2000Low	31	97	29	99	0.2%	1.09 [0.72, 1.66]
SUVIMAX 2010Low	156	6481	178	6536	0.7%	0.88 [0.71, 1.09]
Tam 2005Low	1	20	1	19	0.0%	0.95 [0.06, 14.13]
VEAPS 2002Low	2	177	1	176	0.0%	1.99 [0.18, 21.73]
VECAT 2004Low	20	595	11	598	0.1%	1.83 [0.88, 3.78]
WACS 2007Low	502	4083	124	1022	1.0%	1.01 [0.84, 1.22]
WAVE 2002Low	16	212	6	211	0.0%	2.65 [1.06, 6.65]
White 2002Low	1	50	1	50	0.0%	1.00 [0.06, 15.55]
WHS 2005Low	681	19937	681	19939	3.0%	1.00 [0.90, 1.11]
Witte 2005Low	1	16	1	16	0.0%	1.00 [0.07, 14.64]
Wluka 2002Low	1	67	0	69	0.0%	3.09 [0.13, 74.50]
**Subtotal (95% CI)**		**90245**		**73721**	**94.5%**	**1.01 [0.99, 1.03]**
Total events	11321		9978			
Heterogeneity: Tau² = 0.00; Chi² = 36.47, df = 45 (P = 0.81); I² = 0%						
Test for overall effect: Z = 1.08 (P = 0.28)						
**3.12.2 High Bias Risk**						
H ADCS 1 1997	12	85	12	84	0.1%	0.99 [0.47, 2.07]
H ADCS 2 2005	5	257	5	259	0.0%	1.01 [0.30, 3.44]
H Bonelli 1998	1	147	0	157	0.0%	3.20 [0.13, 78.00]
H Chandra 1992	0	48	2	48	0.0%	0.20 [0.01, 4.06]
H CTNS 2008	77	510	81	510	0.4%	0.95 [0.71, 1.27]
H de la Maza 1995	5	37	4	37	0.0%	1.25 [0.36, 4.29]
H de Waart 2001	0	109	1	109	0.0%	0.33 [0.01, 8.09]
H Gillilan 1977	2	26	2	26	0.0%	1.00 [0.15, 6.57]
H Girodon 1997	12	41	7	20	0.1%	0.84 [0.39, 1.79]
H GISSI 1999	488	5660	529	5664	2.3%	0.92 [0.82, 1.04]
H McKeown-Eys 1988	4	96	3	89	0.0%	1.24 [0.28, 5.37]
H NIT1 1993	1018	14792	280	3698	2.0%	0.91 [0.80, 1.03]
H Penn 1991	1	15	0	15	0.0%	3.00 [0.13, 68.26]
H PPP 2001	72	2231	68	2264	0.3%	1.07 [0.78, 1.49]
H SIT 2001	38	1706	43	1705	0.2%	0.88 [0.57, 1.36]
H Stevic 2001	3	16	6	12	0.0%	0.38 [0.12, 1.20]
H Takagi 2003	10	51	16	42	0.1%	0.51 [0.26, 1.01]
H Takmatsu 1995	1	74	0	73	0.0%	2.96 [0.12, 71.50]
**Subtotal (95% CI)**		**25901**		**14812**	**5.5%**	**0.92 [0.85, 0.99]**
Total events	1749		1059			
Heterogeneity: Tau² = 0.00; Chi² = 9.65, df = 17 (P = 0.92); I² = 0%						
Test for overall effect: Z = 2.18 (P = 0.03)						
**Total (95% CI)**		**116146**		**88533**	**100.0%**	**1.00 [0.99, 1.02]**
Total events	13070		11037			
Heterogeneity: Tau² = 0.00; Chi² = 51.87, df = 63 (P = 0.84); I² = 0%						
Test for overall effect: Z = 0.53 (P = 0.59)						
Test for subgroup differences: Chi² = 5.62, df = 1 (P = 0.02), I² = 82.2%						

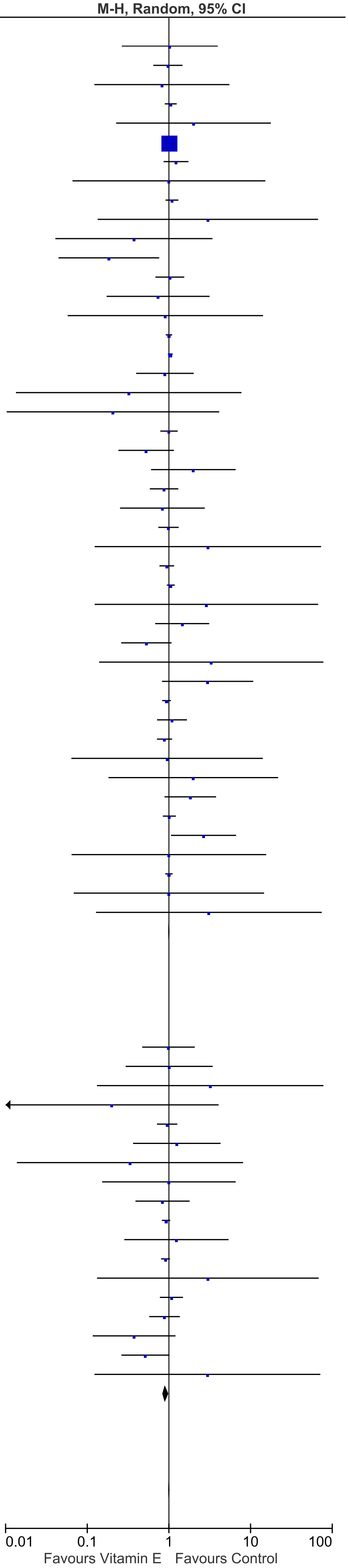

Figure S14 - A11 - ATBC 85-93 Bjelakovic (3 cell)

Study or Subgroup	Vitamin E Events	Vitamin E Total	Control Events	Control Total	Weight	Risk Ratio M-H, Random, 95% CI
**4.4.1 Low Bias Risk**						
Allsup 2004Low	4	81	4	83	0.0%	1.02 [0.27, 3.96]
ALSRT 2001Low	34	144	35	144	0.5%	0.97 [0.64, 1.47]
AMDS 1996Low	2	39	2	32	0.0%	0.82 [0.12, 5.50]
AREDS 2001Low	251	2370	240	2387	3.1%	1.05 [0.89, 1.25]
ASAP 2003Low	4	260	1	130	0.0%	2.00 [0.23, 17.71]
ATBC EB 85-93 03Low	1800	14564	851	7287	15.0%	1.06 [0.98, 1.14]
CHAOS 1996Low	68	1035	52	967	0.7%	1.22 [0.86, 1.73]
Collins 2002Low	1	26	1	26	0.0%	1.00 [0.07, 15.15]
DATATOP 2005Low	154	399	142	401	2.7%	1.09 [0.91, 1.31]
DATOR 2004 Low	1	12	0	12	0.0%	3.00 [0.13, 67.06]
Garbagnatti 2009Low	1	34	3	38	0.0%	0.37 [0.04, 3.41]
Graat 2002Low	3	499	5	153	0.0%	0.18 [0.04, 0.76]
Graf 2005Low	31	83	28	77	0.5%	1.03 [0.68, 1.54]
Grieger 2009Low	3	58	4	57	0.0%	0.74 [0.17, 3.15]
HATS 2001Low	1	84	1	76	0.0%	0.90 [0.06, 14.22]
HOPE_TOO 2005Low	799	4761	801	4780	11.0%	1.00 [0.92, 1.10]
HPS 2002Low	1446	10269	1389	10267	18.8%	1.04 [0.97, 1.11]
ICARE 2008Low	11	726	12	708	0.1%	0.89 [0.40, 2.01]
Jacobson 2000Low	0	57	1	55	0.0%	0.32 [0.01, 7.74]
LAST 2004Low	0	30	2	31	0.0%	0.21 [0.01, 4.13]
Liu 2007Low	96	379	97	384	1.5%	1.00 [0.79, 1.28]
MAVET 2006Low	9	205	17	204	0.1%	0.53 [0.24, 1.15]
MAVIS 2005Low	8	456	4	454	0.1%	1.99 [0.60, 6.57]
Meydani 2004Low	39	311	44	306	0.5%	0.87 [0.58, 1.30]
Mezey 2004Low	4	25	5	26	0.1%	0.83 [0.25, 2.75]
MINVITAOX 1999Low	100	361	51	182	1.1%	0.99 [0.74, 1.32]
Mooney 2005Low	1	142	0	142	0.0%	3.00 [0.12, 73.03]
NIT2 1993Low	157	1657	167	1661	2.1%	0.94 [0.77, 1.16]
PHS 2008Low	857	7329	405	3653	7.1%	1.05 [0.94, 1.18]
Pike 1995Low	1	24	0	23	0.0%	2.88 [0.12, 67.29]
Plummer 2007Low	16	990	11	990	0.2%	1.45 [0.68, 3.12]
PPS 1994Low	15	433	14	214	0.2%	0.53 [0.26, 1.08]
Prince 2003Low	1	29	0	32	0.0%	3.30 [0.14, 77.95]
REACT 2002Low	9	149	3	148	0.1%	2.98 [0.82, 10.79]
SELECT 2009Low	717	17767	382	8856	6.0%	0.94 [0.83, 1.06]
SPACE 2000Low	31	97	29	99	0.5%	1.09 [0.72, 1.66]
SUVIMAX 2010Low	156	6481	178	6536	2.0%	0.88 [0.71, 1.09]
Tam 2005Low	1	20	1	19	0.0%	0.95 [0.06, 14.13]
VEAPS 2002Low	2	177	1	176	0.0%	1.99 [0.18, 21.73]
VECAT 2004Low	20	595	11	598	0.2%	1.83 [0.88, 3.78]
WACS 2007Low	502	4083	124	1022	2.6%	1.01 [0.84, 1.22]
WAVE 2002Low	16	212	6	211	0.1%	2.65 [1.06, 6.65]
White 2002Low	1	50	1	50	0.0%	1.00 [0.06, 15.55]
WHS 2005Low	681	19937	681	19939	8.1%	1.00 [0.90, 1.11]
Witte 2005Low	1	16	1	16	0.0%	1.00 [0.07, 14.64]
Wluka 2002Low	1	67	0	69	0.0%	3.09 [0.13, 74.50]
**Subtotal (95% CI)**		**97523**		**73721**	**85.0%**	**1.02 [0.99, 1.06]**
Total events	8056		5807			
Heterogeneity: Tau² = 0.00; Chi² = 37.37, df = 45 (P = 0.78); I² = 0%						
Test for overall effect: Z = 1.32 (P = 0.19)						
**4.4.2 High Bias Risk**						
H ADCS 1 1997	12	85	12	84	0.2%	0.99 [0.47, 2.07]
H ADCS 2 2005	5	257	5	259	0.1%	1.01 [0.30, 3.44]
H Bonelli 1998	1	147	0	157	0.0%	3.20 [0.13, 78.00]
H Chandra 1992	0	48	2	48	0.0%	0.20 [0.01, 4.06]
H CTNS 2008	77	510	81	510	1.1%	0.95 [0.71, 1.27]
H de la Maza 1995	5	37	4	37	0.1%	1.25 [0.36, 4.29]
H de Waart 2001	0	109	1	109	0.0%	0.33 [0.01, 8.09]
H Gillilan 1977	2	26	2	26	0.0%	1.00 [0.15, 6.57]
H Girodon 1997	12	41	7	20	0.2%	0.84 [0.39, 1.79]
H GISSI 1999	488	5660	529	5664	6.4%	0.92 [0.82, 1.04]
H McKeown-Eys 1988	4	96	3	89	0.0%	1.24 [0.28, 5.37]
H NIT1 1993	1018	14792	280	3698	5.4%	0.91 [0.80, 1.03]
H Penn 1991	1	15	0	15	0.0%	3.00 [0.13, 68.26]
H PPP 2001	72	2231	68	2264	0.8%	1.07 [0.78, 1.49]
H SIT 2001	38	1706	43	1705	0.5%	0.88 [0.57, 1.36]
H Stevic 2001	3	16	6	12	0.1%	0.38 [0.12, 1.20]
H Takagi 2003	10	51	16	42	0.2%	0.51 [0.26, 1.01]
H Takmatsu 1995	1	74	0	73	0.0%	2.96 [0.12, 71.50]
**Subtotal (95% CI)**		**25901**		**14812**	**15.0%**	**0.92 [0.85, 0.99]**
Total events	1749		1059			
Heterogeneity: Tau² = 0.00; Chi² = 9.65, df = 17 (P = 0.92); I² = 0%						
Test for overall effect: Z = 2.18 (P = 0.03)						
**Total (95% CI)**		**123424**		**88533**	**100.0%**	**1.01 [0.98, 1.04]**
Total events	9805		6866			
Heterogeneity: Tau² = 0.00; Chi² = 53.36, df = 63 (P = 0.80); I² = 0%						
Test for overall effect: Z = 0.37 (P = 0.71)						
Test for subgroup differences: Chi² = 6.35, df = 1 (P = 0.01), I² = 84.2%						

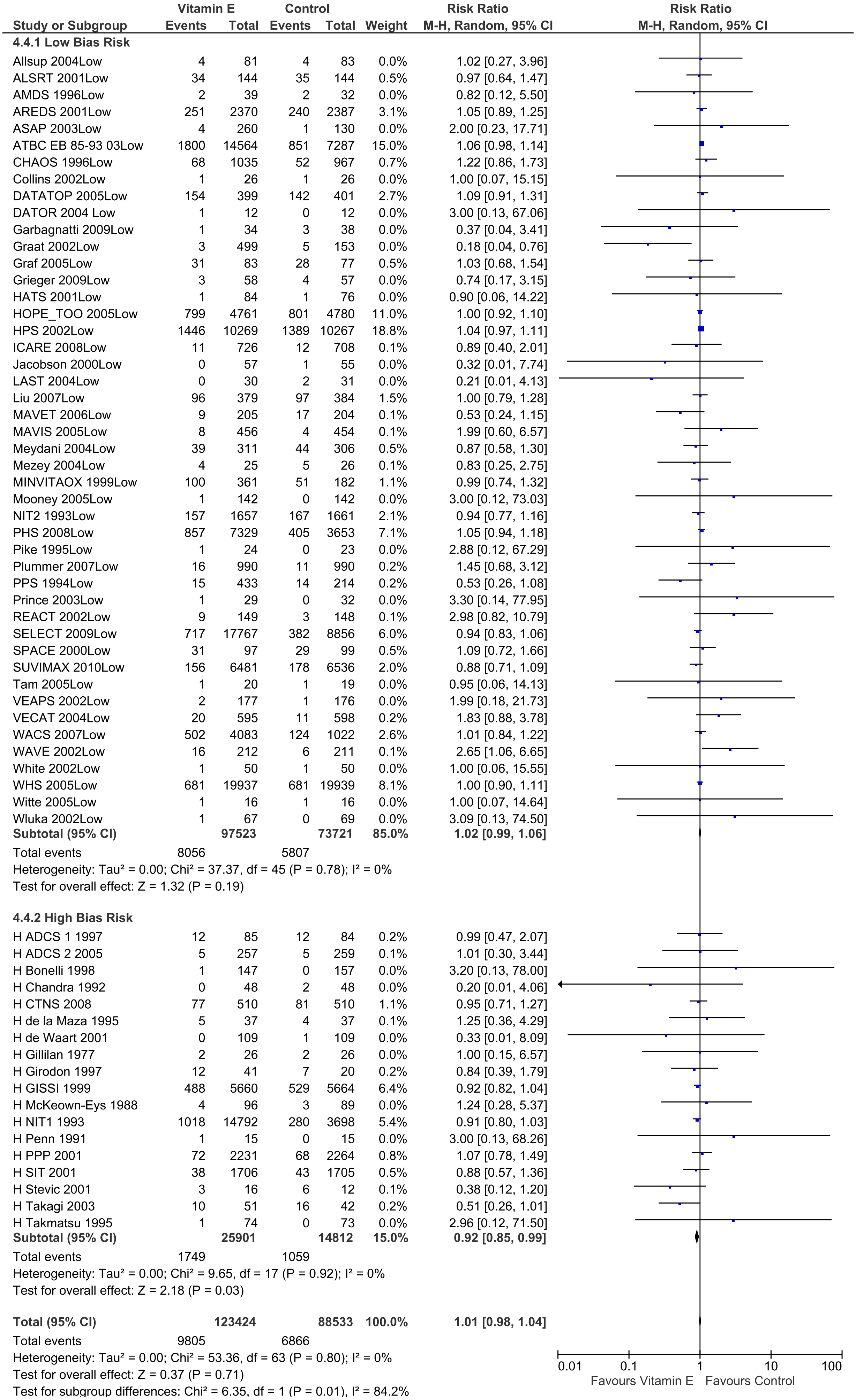

Figure S15 - A11 - ATBC 85-96 Bjelakovic (3 cell)

Study or Subgroup	Vitamin E Events	Vitamin E Total	Control Events	Control Total	Weight	Risk Ratio M-H, Random, 95% CI
**4.5.1 Low Bias Risk**						
Allsup 2004Low	4	81	4	83	0.0%	1.02 [0.27, 3.96]
ALSRT 2001Low	34	144	35	144	0.5%	0.97 [0.64, 1.47]
AMDS 1996Low	2	39	2	32	0.0%	0.82 [0.12, 5.50]
AREDS 2001Low	251	2370	240	2387	2.8%	1.05 [0.89, 1.25]
ASAP 2003Low	4	260	1	130	0.0%	2.00 [0.23, 17.71]
ATBC EB 85-96 03Low	2993	14564	1449	7287	24.7%	1.03 [0.98, 1.09]
CHAOS 1996Low	68	1035	52	967	0.6%	1.22 [0.86, 1.73]
Collins 2002Low	1	26	1	26	0.0%	1.00 [0.07, 15.15]
DATATOP 2005Low	154	399	142	401	2.4%	1.09 [0.91, 1.31]
DATOR 2004 Low	1	12	0	12	0.0%	3.00 [0.13, 67.06]
Garbagnatti 2009Low	1	34	3	38	0.0%	0.37 [0.04, 3.41]
Graat 2002Low	3	499	5	153	0.0%	0.18 [0.04, 0.76]
Graf 2005Low	31	83	28	77	0.5%	1.03 [0.68, 1.54]
Grieger 2009Low	3	58	4	57	0.0%	0.74 [0.17, 3.15]
HATS 2001Low	1	84	1	76	0.0%	0.90 [0.06, 14.22]
HOPE_TOO 2005Low	799	4761	801	4780	9.7%	1.00 [0.92, 1.10]
HPS 2002Low	1446	10269	1389	10267	16.6%	1.04 [0.97, 1.11]
ICARE 2008Low	11	726	12	708	0.1%	0.89 [0.40, 2.01]
Jacobson 2000Low	0	57	1	55	0.0%	0.32 [0.01, 7.74]
LAST 2004Low	0	30	2	31	0.0%	0.21 [0.01, 4.13]
Liu 2007Low	96	379	97	384	1.3%	1.00 [0.79, 1.28]
MAVET 2006Low	9	205	17	204	0.1%	0.53 [0.24, 1.15]
MAVIS 2005Low	8	456	4	454	0.1%	1.99 [0.60, 6.57]
Meydani 2004Low	39	311	44	306	0.5%	0.87 [0.58, 1.30]
Mezey 2004Low	4	25	5	26	0.1%	0.83 [0.25, 2.75]
MINVITAOX 1999Low	100	361	51	182	0.9%	0.99 [0.74, 1.32]
Mooney 2005Low	1	142	0	142	0.0%	3.00 [0.12, 73.03]
NIT2 1993Low	157	1657	167	1661	1.8%	0.94 [0.77, 1.16]
PHS 2008Low	857	7329	405	3653	6.3%	1.05 [0.94, 1.18]
Pike 1995Low	1	24	0	23	0.0%	2.88 [0.12, 67.29]
Plummer 2007Low	16	990	11	990	0.1%	1.45 [0.68, 3.12]
PPS 1994Low	15	433	14	214	0.2%	0.53 [0.26, 1.08]
Prince 2003Low	1	29	0	32	0.0%	3.30 [0.14, 77.95]
REACT 2002Low	9	149	3	148	0.0%	2.98 [0.82, 10.79]
SELECT 2009Low	717	17767	382	8856	5.3%	0.94 [0.83, 1.06]
SPACE 2000Low	31	97	29	99	0.4%	1.09 [0.72, 1.66]
SUVIMAX 2010Low	156	6481	178	6536	1.7%	0.88 [0.71, 1.09]
Tam 2005Low	1	20	1	19	0.0%	0.95 [0.06, 14.13]
VEAPS 2002Low	2	177	1	176	0.0%	1.99 [0.18, 21.73]
VECAT 2004Low	20	595	11	598	0.1%	1.83 [0.88, 3.78]
WACS 2007Low	502	4083	124	1022	2.3%	1.01 [0.84, 1.22]
WAVE 2002Low	16	212	6	211	0.1%	2.65 [1.06, 6.65]
White 2002Low	1	50	1	50	0.0%	1.00 [0.06, 15.55]
WHS 2005Low	681	19937	681	19939	7.1%	1.00 [0.90, 1.11]
Witte 2005Low	1	16	1	16	0.0%	1.00 [0.07, 14.64]
Wluka 2002Low	1	67	0	69	0.0%	3.09 [0.13, 74.50]
**Subtotal (95% CI)**		**97523**		**73721**	**86.8%**	**1.02 [0.99, 1.05]**
Total events	9249		6405			
Heterogeneity: Tau² = 0.00; Chi² = 36.71, df = 45 (P = 0.81); I² = 0%						
Test for overall effect: Z = 1.27 (P = 0.20)						
**4.5.2 High Bias Risk**						
H ADCS 1 1997	12	85	12	84	0.1%	0.99 [0.47, 2.07]
H ADCS 2 2005	5	257	5	259	0.1%	1.01 [0.30, 3.44]
H Bonelli 1998	1	147	0	157	0.0%	3.20 [0.13, 78.00]
H Chandra 1992	0	48	2	48	0.0%	0.20 [0.01, 4.06]
H CTNS 2008	77	510	81	510	0.9%	0.95 [0.71, 1.27]
H de la Maza 1995	5	37	4	37	0.1%	1.25 [0.36, 4.29]
H de Waart 2001	0	109	1	109	0.0%	0.33 [0.01, 8.09]
H Gillilan 1977	2	26	2	26	0.0%	1.00 [0.15, 6.57]
H Girodon 1997	12	41	7	20	0.1%	0.84 [0.39, 1.79]
H GISSI 1999	488	5660	529	5664	5.6%	0.92 [0.82, 1.04]
H McKeown-Eys 1988	4	96	3	89	0.0%	1.24 [0.28, 5.37]
H NIT1 1993	1018	14792	280	3698	4.8%	0.91 [0.80, 1.03]
H Penn 1991	1	15	0	15	0.0%	3.00 [0.13, 68.26]
H PPP 2001	72	2231	68	2264	0.7%	1.07 [0.78, 1.49]
H SIT 2001	38	1706	43	1705	0.4%	0.88 [0.57, 1.36]
H Stevic 2001	3	16	6	12	0.1%	0.38 [0.12, 1.20]
H Takagi 2003	10	51	16	42	0.2%	0.51 [0.26, 1.01]
H Takmatsu 1995	1	74	0	73	0.0%	2.96 [0.12, 71.50]
**Subtotal (95% CI)**		**25901**		**14812**	**13.2%**	**0.92 [0.85, 0.99]**
Total events	1749		1059			
Heterogeneity: Tau² = 0.00; Chi² = 9.65, df = 17 (P = 0.92); I² = 0%						
Test for overall effect: Z = 2.18 (P = 0.03)						
**Total (95% CI)**		**123424**		**88533**	**100.0%**	**1.01 [0.98, 1.03]**
Total events	10998		7464			
Heterogeneity: Tau² = 0.00; Chi² = 52.57, df = 63 (P = 0.82); I² = 0%						
Test for overall effect: Z = 0.39 (P = 0.69)						
Test for subgroup differences: Chi² = 6.22, df = 1 (P = 0.01), I² = 83.9%						

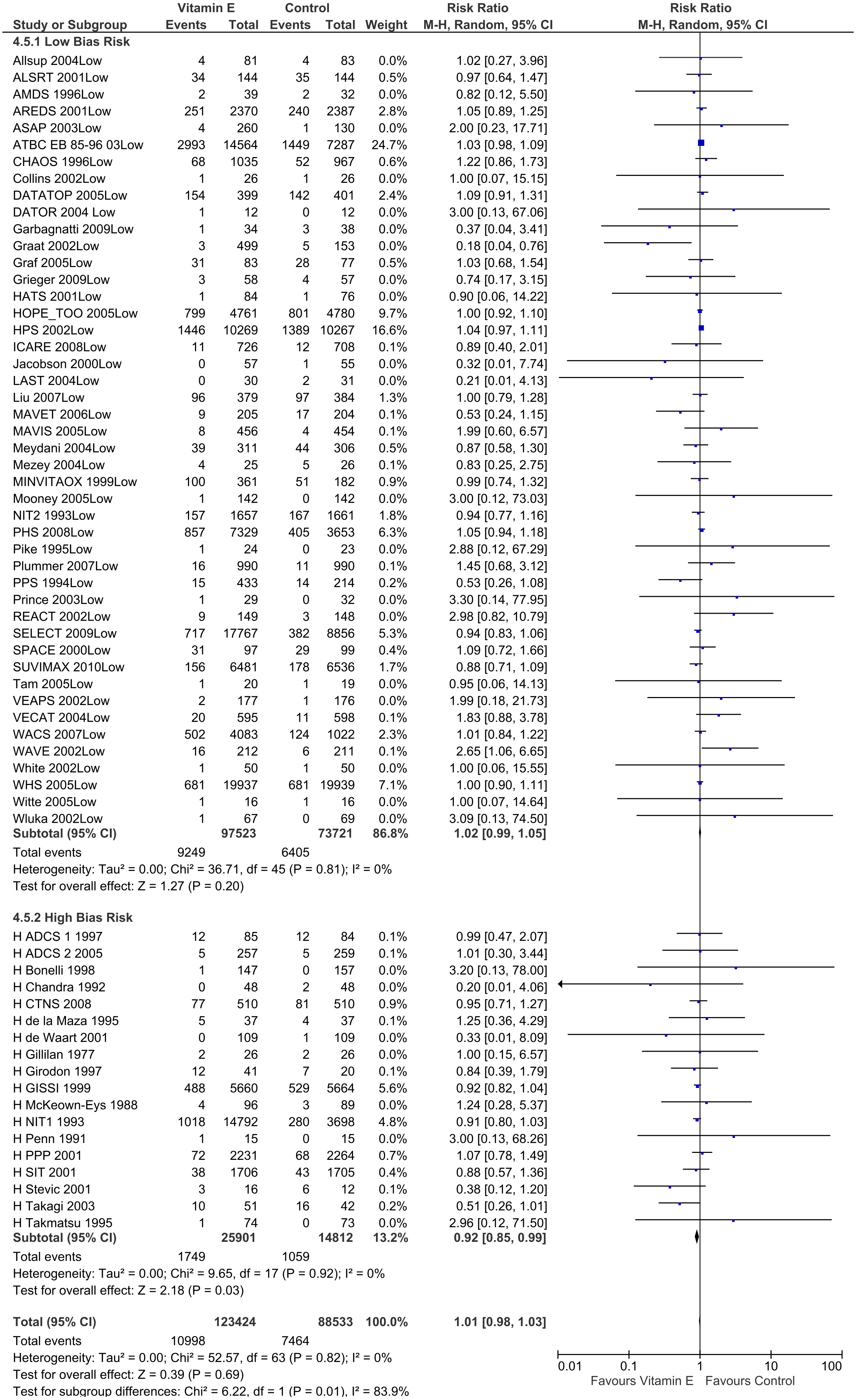

Figure S16 - A11 - ATBC 85-99 Bjelakovic (3 cell)

Study or Subgroup	Vitamin E Events	Vitamin E Total	Control Events	Control Total	Weight	Risk Ratio M-H, Random, 95% CI
**4.6.1 Low Bias Risk**						
Allsup 2004Low	4	81	4	83	0.0%	1.02 [0.27, 3.96]
ALSRT 2001Low	34	144	35	144	0.4%	0.97 [0.64, 1.47]
AMDS 1996Low	2	39	2	32	0.0%	0.82 [0.12, 5.50]
AREDS 2001Low	251	2370	240	2387	2.4%	1.05 [0.89, 1.25]
ASAP 2003Low	4	260	1	130	0.0%	2.00 [0.23, 17.71]
ATBC EB 85-99 03Low	4453	14564	2117	7287	35.4%	1.05 [1.01, 1.10]
CHAOS 1996Low	68	1035	52	967	0.5%	1.22 [0.86, 1.73]
Collins 2002Low	1	26	1	26	0.0%	1.00 [0.07, 15.15]
DATATOP 2005Low	154	399	142	401	2.0%	1.09 [0.91, 1.31]
DATOR 2004 Low	1	12	0	12	0.0%	3.00 [0.13, 67.06]
Garbagnatti 2009Low	1	34	3	38	0.0%	0.37 [0.04, 3.41]
Graat 2002Low	3	499	5	153	0.0%	0.18 [0.04, 0.76]
Graat_EO 2002Low	31	83	28	77	0.4%	1.03 [0.68, 1.54]
Graf 2005Low	3	58	4	57	0.0%	0.74 [0.17, 3.15]
Grieger 2009Low	1	84	1	76	0.0%	0.90 [0.06, 14.22]
HATS 2001Low	799	4761	801	4780	8.4%	1.00 [0.92, 1.10]
HOPE_TOO 2005Low	1446	10269	1389	10267	14.3%	1.04 [0.97, 1.11]
HPS 2002Low	11	726	12	708	0.1%	0.89 [0.40, 2.01]
ICARE 2008Low	0	57	1	55	0.0%	0.32 [0.01, 7.74]
Jacobson 2000Low	0	30	2	31	0.0%	0.21 [0.01, 4.13]
LAST 2004Low	96	379	97	384	1.1%	1.00 [0.79, 1.28]
Liu 2007Low	9	205	17	204	0.1%	0.53 [0.24, 1.15]
MAVET 2006Low	8	456	4	454	0.0%	1.99 [0.60, 6.57]
MAVIS 2005Low	39	311	44	306	0.4%	0.87 [0.58, 1.30]
Meydani 2004Low	4	25	5	26	0.0%	0.83 [0.25, 2.75]
Mezey 2004Low	100	361	51	182	0.8%	0.99 [0.74, 1.32]
MINVITAOX 1999Low	1	142	0	142	0.0%	3.00 [0.12, 73.03]
Mooney 2005Low	157	1657	167	1661	1.6%	0.94 [0.77, 1.16]
NIT2 1993Low	857	7329	405	3653	5.4%	1.05 [0.94, 1.18]
PHS 2008Low	1	24	0	23	0.0%	2.88 [0.12, 67.29]
Pike 1995Low	16	990	11	990	0.1%	1.45 [0.68, 3.12]
Plummer 2007Low	15	433	14	214	0.1%	0.53 [0.26, 1.08]
PPS 1994Low	1	29	0	32	0.0%	3.30 [0.14, 77.95]
Prince 2003Low	9	149	3	148	0.0%	2.98 [0.82, 10.79]
REACT 2002Low	717	17767	382	8856	4.5%	0.94 [0.83, 1.06]
SELECT 2009Low	31	97	29	99	0.4%	1.09 [0.72, 1.66]
SPACE 2000Low	156	6481	178	6536	1.5%	0.88 [0.71, 1.09]
SUVIMAX 2010Low	1	20	1	19	0.0%	0.95 [0.06, 14.13]
Tam 2005Low	2	177	1	176	0.0%	1.99 [0.18, 21.73]
VEAPS 2002Low	20	595	11	598	0.1%	1.83 [0.88, 3.78]
VECAT 2004Low	502	4083	124	1022	2.0%	1.01 [0.84, 1.22]
WACS 2007Low	16	212	6	211	0.1%	2.65 [1.06, 6.65]
WAVE 2002Low	1	50	1	50	0.0%	1.00 [0.06, 15.55]
White 2002Low	681	19937	681	19939	6.1%	1.00 [0.90, 1.11]
WHS 2005Low	1	16	1	16	0.0%	1.00 [0.07, 14.64]
Witte 2005Low	1	67	0	69	0.0%	3.09 [0.13, 74.50]
Wluka 2002Low	1	67	0	69	0.0%	3.09 [0.13, 74.50]
**Subtotal (95% CI)**		**97590**		**73790**	**88.6%**	**1.03 [1.00, 1.06]**
Total events	10710		7073			
Heterogeneity: Tau² = 0.00; Chi² = 38.56, df = 46 (P = 0.77); I² = 0%						
Test for overall effect: Z = 2.07 (P = 0.04)						
**4.6.2 High Bias Risk**						
H ADCS 1 1997	12	85	12	84	0.1%	0.99 [0.47, 2.07]
H ADCS 2 2005	5	257	5	259	0.0%	1.01 [0.30, 3.44]
H Bonelli 1998	1	147	0	157	0.0%	3.20 [0.13, 78.00]
H Chandra 1992	0	48	2	48	0.0%	0.20 [0.01, 4.06]
H CTNS 2008	77	510	81	510	0.8%	0.95 [0.71, 1.27]
H de la Maza 1995	5	37	4	37	0.0%	1.25 [0.36, 4.29]
H de Waart 2001	0	109	1	109	0.0%	0.33 [0.01, 8.09]
H Gillilan 1977	2	26	2	26	0.0%	1.00 [0.15, 6.57]
H Girodon 1997	12	41	7	20	0.1%	0.84 [0.39, 1.79]
H GISSI 1999	488	5660	529	5664	4.8%	0.92 [0.82, 1.04]
H McKeown-Eys 1988	4	96	3	89	0.0%	1.24 [0.28, 5.37]
H NIT1 1993	1018	14792	280	3698	4.1%	0.91 [0.80, 1.03]
H Penn 1991	1	15	0	15	0.0%	3.00 [0.13, 68.26]
H PPP 2001	72	2231	68	2264	0.6%	1.07 [0.78, 1.49]
H SIT 2001	38	1706	43	1705	0.4%	0.88 [0.57, 1.36]
H Stevic 2001	3	16	6	12	0.0%	0.38 [0.12, 1.20]
H Takagi 2003	10	51	16	42	0.1%	0.51 [0.26, 1.01]
H Takmatsu 1995	1	74	0	73	0.0%	2.96 [0.12, 71.50]
**Subtotal (95% CI)**		**25901**		**14812**	**11.4%**	**0.92 [0.85, 0.99]**
Total events	1749		1059			
Heterogeneity: Tau² = 0.00; Chi² = 9.65, df = 17 (P = 0.92); I² = 0%						
Test for overall effect: Z = 2.18 (P = 0.03)						
**Total (95% CI)**		**123491**		**88602**	**100.0%**	**1.02 [0.99, 1.04]**
Total events	12459		8132			
Heterogeneity: Tau² = 0.00; Chi² = 55.78, df = 64 (P = 0.76); I² = 0%						
Test for overall effect: Z = 1.21 (P = 0.23)						
Test for subgroup differences: Chi² = 7.56, df = 1 (P = 0.006), I² = 86.8%						

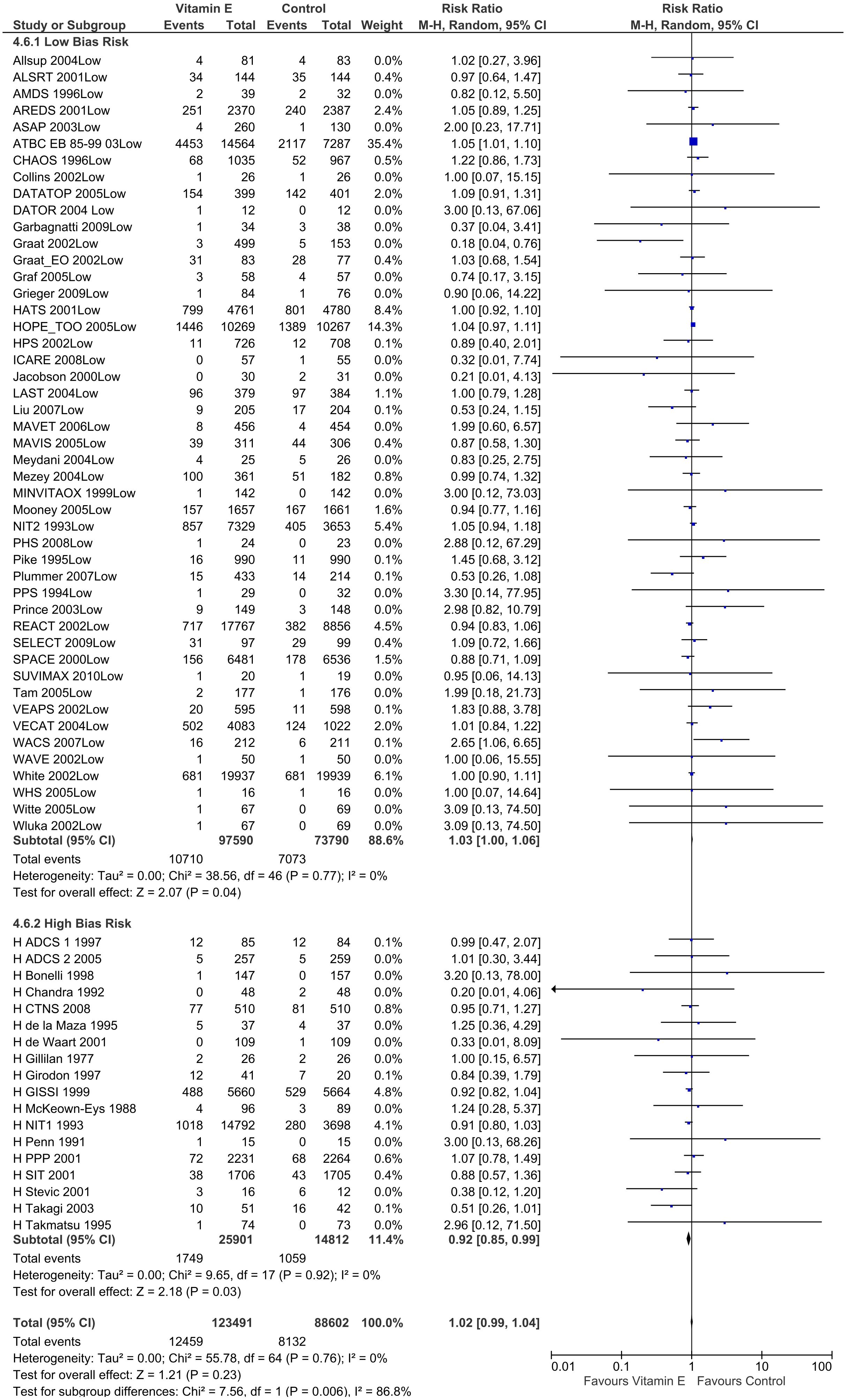

Figure S17 - A11 - ATBC 85-11 Bjelakovic (3 cell)

Study or Subgroup	Vitamin E Events	Vitamin E Total	Control Events	Control Total	Weight	Risk Ratio M-H, Random, 95% CI
**4.7.1 Low Risk Bias**						
Allsup 2004Low	4	81	4	83	0.0%	1.02 [0.27, 3.96]
ALSRT 2001Low	34	144	35	144	0.2%	0.97 [0.64, 1.47]
AMDS 1996Low	2	39	2	32	0.0%	0.82 [0.12, 5.50]
AREDS 2001Low	251	2370	240	2387	0.9%	1.05 [0.89, 1.25]
ASAP 2003Low	4	260	1	130	0.0%	2.00 [0.23, 17.71]
ATBC EB 85-11 3 cell	10182	14564	5022	7287	74.6%	1.01 [1.00, 1.03]
CHAOS 1996Low	68	1035	52	967	0.2%	1.22 [0.86, 1.73]
Collins 2002Low	1	26	1	26	0.0%	1.00 [0.07, 15.15]
DATATOP 2005Low	154	399	142	401	0.8%	1.09 [0.91, 1.31]
DATOR 2004 Low	1	12	0	12	0.0%	3.00 [0.13, 67.06]
Garbagnatti 2009Low	1	34	3	38	0.0%	0.37 [0.04, 3.41]
Graat 2002Low	3	499	5	153	0.0%	0.18 [0.04, 0.76]
Graf 2005Low	31	83	28	77	0.2%	1.03 [0.68, 1.54]
Grieger 2009Low	3	58	4	57	0.0%	0.74 [0.17, 3.15]
HATS 2001Low	1	84	1	76	0.0%	0.90 [0.06, 14.22]
HOPE_TOO 2005Low	799	4761	801	4780	3.3%	1.00 [0.92, 1.10]
HPS 2002Low	1446	10269	1389	10267	5.6%	1.04 [0.97, 1.11]
ICARE 2008Low	11	726	12	708	0.0%	0.89 [0.40, 2.01]
Jacobson 2000Low	0	57	1	55	0.0%	0.32 [0.01, 7.74]
LAST 2004Low	0	30	2	31	0.0%	0.21 [0.01, 4.13]
Liu 2007Low	96	379	97	384	0.4%	1.00 [0.79, 1.28]
MAVET 2006Low	9	205	17	204	0.0%	0.53 [0.24, 1.15]
MAVIS 2005Low	8	456	4	454	0.0%	1.99 [0.60, 6.57]
Meydani 2004Low	39	311	44	306	0.2%	0.87 [0.58, 1.30]
Mezey 2004Low	4	25	5	26	0.0%	0.83 [0.25, 2.75]
MINVITAOX 1999Low	100	361	51	182	0.3%	0.99 [0.74, 1.32]
Mooney 2005Low	1	142	0	142	0.0%	3.00 [0.12, 73.03]
NIT2 1993Low	157	1657	167	1661	0.6%	0.94 [0.77, 1.16]
PHS 2008Low	857	7329	405	3653	2.1%	1.05 [0.94, 1.18]
Pike 1995Low	1	24	0	23	0.0%	2.88 [0.12, 67.29]
Plummer 2007Low	16	990	11	990	0.0%	1.45 [0.68, 3.12]
PPS 1994Low	15	433	14	214	0.1%	0.53 [0.26, 1.08]
Prince 2003Low	1	29	0	32	0.0%	3.30 [0.14, 77.95]
REACT 2002Low	9	149	3	148	0.0%	2.98 [0.82, 10.79]
SELECT 2009Low	717	17767	382	8856	1.8%	0.94 [0.83, 1.06]
SPACE 2000Low	31	97	29	99	0.1%	1.09 [0.72, 1.66]
SUVIMAX 2010Low	156	6481	178	6536	0.6%	0.88 [0.71, 1.09]
Tam 2005Low	1	20	1	19	0.0%	0.95 [0.06, 14.13]
VEAPS 2002Low	2	177	1	176	0.0%	1.99 [0.18, 21.73]
VECAT 2004Low	20	595	11	598	0.0%	1.83 [0.88, 3.78]
WACS 2007Low	502	4083	124	1022	0.8%	1.01 [0.84, 1.22]
WAVE 2002Low	16	212	6	211	0.0%	2.65 [1.06, 6.65]
White 2002Low	1	50	1	50	0.0%	1.00 [0.06, 15.55]
WHS 2005Low	681	19937	681	19939	2.4%	1.00 [0.90, 1.11]
Witte 2005Low	1	16	1	16	0.0%	1.00 [0.07, 14.64]
Wluka 2002Low	1	67	0	69	0.0%	3.09 [0.13, 74.50]
**Subtotal (95% CI)**		**97523**		**73721**	**95.5%**	**1.01 [1.00, 1.03]**
Total events	16438		9978			
Heterogeneity: Tau² = 0.00; Chi² = 36.40, df = 45 (P = 0.82); I² = 0%						
Test for overall effect: Z = 1.69 (P = 0.09)						
**4.7.2 High Risk Bias**						
H ADCS 1 1997	12	85	12	84	0.0%	0.99 [0.47, 2.07]
H ADCS 2 2005	5	257	5	259	0.0%	1.01 [0.30, 3.44]
H Bonelli 1998	1	147	0	157	0.0%	3.20 [0.13, 78.00]
H Chandra 1992	0	48	2	48	0.0%	0.20 [0.01, 4.06]
H CTNS 2008	77	510	81	510	0.3%	0.95 [0.71, 1.27]
H de la Maza 1995	5	37	4	37	0.0%	1.25 [0.36, 4.29]
H de Waart 2001	0	109	1	109	0.0%	0.33 [0.01, 8.09]
H Gillilan 1977	2	26	2	26	0.0%	1.00 [0.15, 6.57]
H Girodon 1997	12	41	7	20	0.0%	0.84 [0.39, 1.79]
H GISSI 1999	488	5660	529	5664	1.9%	0.92 [0.82, 1.04]
H McKeown-Eys 1988	4	96	3	89	0.0%	1.24 [0.28, 5.37]
H NIT1 1993	1018	14792	280	3698	1.6%	0.91 [0.80, 1.03]
H Penn 1991	1	15	0	15	0.0%	3.00 [0.13, 68.26]
H PPP 2001	72	2231	68	2264	0.2%	1.07 [0.78, 1.49]
H SIT 2001	38	1706	43	1705	0.1%	0.88 [0.57, 1.36]
H Stevic 2001	3	16	6	12	0.0%	0.38 [0.12, 1.20]
H Takagi 2003	10	51	16	42	0.1%	0.51 [0.26, 1.01]
H Takmatsu 1995	1	74	0	73	0.0%	2.96 [0.12, 71.50]
**Subtotal (95% CI)**		**25901**		**14812**	**4.5%**	**0.92 [0.85, 0.99]**
Total events	1749		1059			
Heterogeneity: Tau² = 0.00; Chi² = 9.65, df = 17 (P = 0.92); I² = 0%						
Test for overall effect: Z = 2.18 (P = 0.03)						
**Total (95% CI)**		**123424**		**88533**	**100.0%**	**1.01 [0.99, 1.03]**
Total events	18187		11037			
Heterogeneity: Tau² = 0.00; Chi² = 52.54, df = 63 (P = 0.82); I² = 0%						
Test for overall effect: Z = 1.19 (P = 0.23)						
Test for subgroup differences: Chi² = 6.19, df = 1 (P = 0.01), I² = 83.8%						

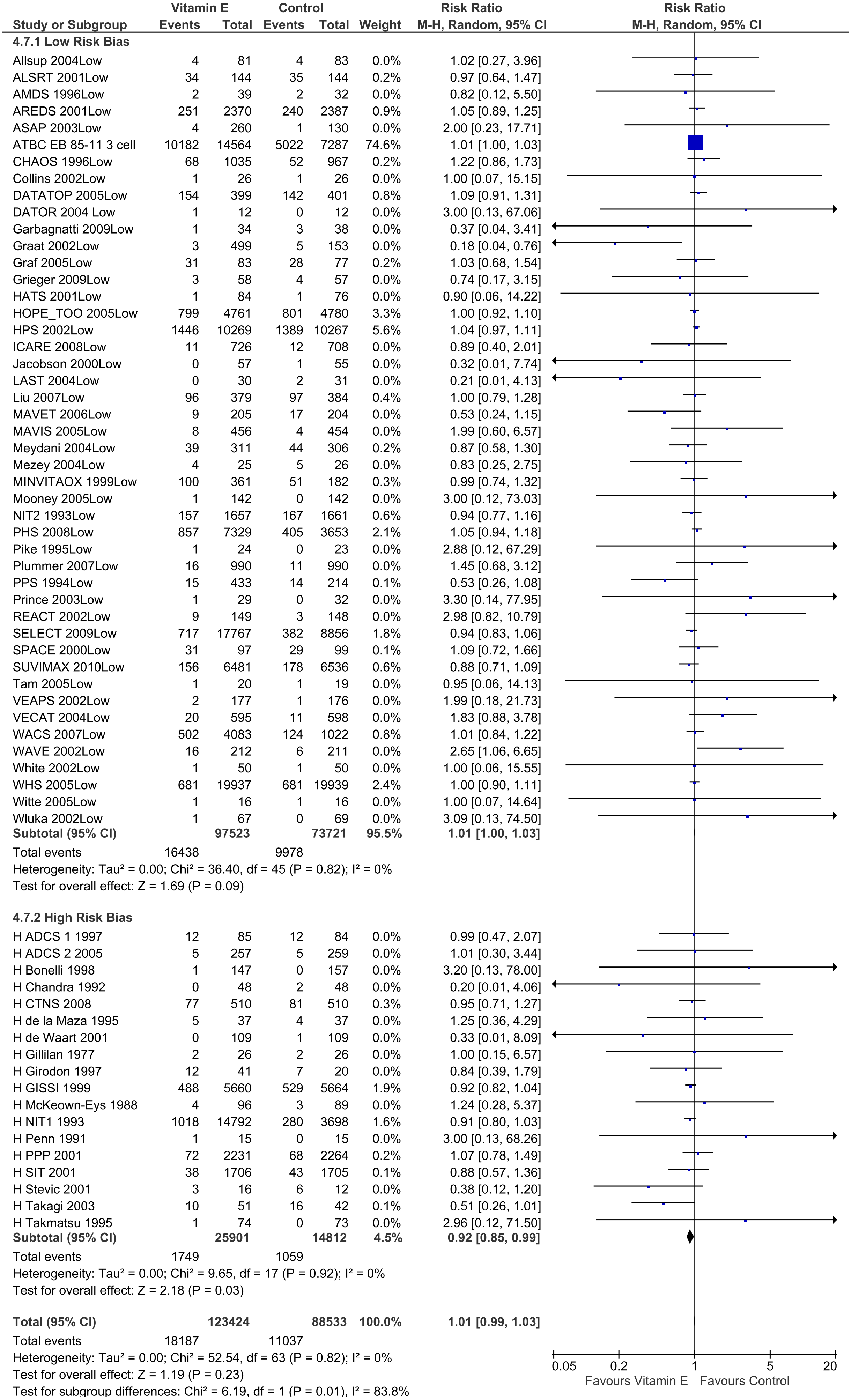

Supplement: Additional file 1 — Fig. S1. A11 - ATBC 85–01 as per Bjelakovic 2012. Fig. S2. A11 - Minus ATBC 85–01. Fig. S3. A11 - Plus Fictitious Trial. Fig. S4. A11 - ATBC 85–93 - At the Margins (4 cell) Analysis. Fig. S5. A11 - ATBC 85–96 - At the Margins (4 cell) Analysis. Fig. S6. A11 - ATBC 85–99 - At the Margins (4 cell) Analysis. Fig. S7. A11 - ATBC 85–01 - At the Margins (4 cell) Analysis. Fig. S8. A11 - ATBC 85–11 - At the Margins (4 cell) Analysis. Fig. S9. A11 - ATBC 85–93 - Inside the Table (2 cell) Analysis. Fig. S10. A11 - ATBC 85–96 - Inside the Table (2 cell) Analysis. Fig. S11. A11 - ATBC 85–99 - Inside the Table (2 cell) Analysis. Fig. S12. A11 - ATBC 85–01 - Inside the Table (2 cell) Analysis. Fig. S13. A11 - ATBC 85–11 - Inside the Table (2 cell) Analysis. Fig. S14. A11 - ATBC 85–93 - Bjelakovic (3 cell) Analysis. Fig. S15. A11 - ATBC 85–96 - Bjelakovic (3 cell) Analysis. Fig. S16. A11 - ATBC 85–99 - Bjelakovic (3 cell) Analysis. Fig. S17. A11 - ATBC 85–11 - Bjelakovic (3 cell) Analysis. (PDF 12335 kb) [file 12906_2017_1906_MOESM1_ESM.pdf]
