# Supplementary material for: Validity of a Cochrane Systematic Review and meta-analysis for determining the safety of vitamin E
Source: BMC Complement Altern Med. 2017 Aug 16;17:408. doi: 10.1186/s12906-017-1906-x (PMC5565069; doi:10.1186/s12906-017-1906-x)
Supplement: Related texts

# Reply: Vitamin E supplementation for prevention of mortality in adults

**Authors:** Goran Bjelakovic, Dimitrinka Nikolova, Christian Gluud

**Affiliations:** 4 0000 0004 0646 7373 grid.4973.9 The Cochrane Hepato-Biliary Group, Copenhagen Trial Unit, Centre for Clinical Intervention Research, Department 7812, Rigshospitalet, Copenhagen University Hospital, Copenhagen, Denmark; 5 0000 0001 0942 1176 grid.11374.30 Department of Internal Medicine - Gastroenterology and Hepatology, Medical Faculty, University of Nis, Nis, Serbia

We read with interest the article by Oliver and Myers questioning the validity of the results of vitamin E on all-cause mortality in our systematic review [2, 17]. The authors reanalysed some of the meta-analyses in our systematic review, using several scenarios which led them to questioning our findings of increased mortality in people supplemented with vitamin E [2, 17].

For all analyses in our review, we extracted data using the same methods [2]. In the trials with parallel group design with more than two intervention groups and additional therapy, we compared the groups with only antioxidant intervention versus placebo or no intervention [2]. For trials with factorial design, we compared all groups that received antioxidant supplements versus placebo or no intervention group [2]. Similarly, in factorial trials that tested vitamin E, we compared all groups supplemented with vitamin E versus placebo or no intervention group. Among the 46 trials with a low risk of bias included in our Analysis 1.11, ‘Mortality in vitamin E trials with low risk of bias’ there are 8 factorial trials (6 trials with 2 by 2 factorial design, and 2 trials with 2 by 2 by 2 factorial design). Some of these factorial trials tested vitamin E singly or combined with beta-carotene (found to have a potential detrimental effect on mortality), while the remaining factorial trials tested vitamin E singly or combined with selenium (found to have a potential beneficial effect on mortality), or vitamin E singly or combined with vitamin C (found to have a neutral effect on mortality) [2] In some of the included parallel group trials, vitamin E was tested combined with multivitamins or multiminerals. The most commonly used dietary supplements in adults are multivitamins or multiminerals [18]. Therefore, there is a possibility of synergistic or antagonistic interactions between tested antioxidants. We believe we used the best available approach to analyse beneficial and harmful effects of vitamin E. Furthermore, we assessed whether the different doses of beta-carotene, vitamin A, and vitamin E affect mortality in primary and secondary prevention randomised clinical trials at low risk of bias [19]. We found that beta-carotene and vitamin E in doses higher than the RDA seem to significantly increase mortality [19].

Our results are in accordance with the results of Miller and colleagues who used ‘at margins’ or ‘2-cell’ analysis, who also found increased mortality in people supplemented with high doses of vitamin E [4].

In future updates of our review, we shall consider also to employ the ‘at margins’ methodology suggested by Oliver and Myers. At the same time, we notice that in none of the scenario analyses conducted by Oliver and Myers, vitamin E showed any significant benefit [17]. Moreover, as our own analysis 1.16 show antioxidants without potential influence from confounders in factorial trials versus placebo or no intervention causes a significant increase in all-cause mortality of 10% (relative risk 1.10; 95% CI 1.05 to 1.15; *P* = 0.0002; I^2^ = 0%).

As we mentioned in our Cochrane review, we performed our review according to the recommendations of the Cochrane Handbook for Systematic Reviews of Interventions [20]. Accordingly, we assessed outcome data at the end of the trial follow-up period for each included trial, making it uniform for the whole review. Based on our meta-regression analyses stratified by the intervention regimen, the duration of follow-up was not a predictor of the estimated intervention effect [2].

We have extensively discussed the issue of different forms of vitamin E used for supplementation. We could not include trials with tocotrienols, gamma tocopherol, or any other form of vitamin E because such randomised clinical trials were not published at that time. The majority of the trials conducted tested alpha-tocopherol.

During recent years, several major randomised clinical trials on vitamins and supplements have produced disappointing results. One recent example is the Selenium and Vitamin E Cancer Prevention Trial (SELECT), originally scheduled to end in 2011 [21]. SELECT was terminated in October, 2008 over a disproportionally high incidence of prostate cancer in trial participants supplemented with vitamin E [21]. The chance that vitamin E may provide benefit seems low [22–24].

We have dealt with trials with zero mortality extensively in our review. Trials without deaths were excluded. First, they provide very little information. Second, most of these trials were not really ‘preventive trials’, but rather explanatory trials assessing the impact of antioxidant supplements on potential surrogate outcomes. Third, we assessed the influence of trials with zero events in the treatment or control group by re-calculating the random-effects meta-analyses with 0.5, 0.05, and 0.005 continuity corrections. Here our results were robust. Fourth, our results were also robust to exploratory analyses adding an imagined trial with 20 000 participants and one death in each intervention group. Accordingly, the increased mortality does not seem to be an artifact created by exclusion of trials with zero events in both trial groups.

We are aware that pharmaceutical companies are influenced by our findings. Antioxidants together with other food supplements have turned into multi-billion $ markets without much evidence supporting their use. ‘Vitamins, Inc.’ was the most elaborate, longest lasting, and most harmful of all the international cartels ever discovered [25]. Pharmaceutical companies have been cheating us into buying vitamin supplements at too high prices [25]. If harmful effects of antioxidant supplements stand to the test of science and time, then we may end up realising that we have also been fooled into believing ‘the more antioxidant supplements, the merrier’.
